# A mega-phylogeny of the Annonaceae: taxonomic placement of five enigmatic genera and support for a new tribe, Phoenicantheae

**DOI:** 10.1038/s41598-017-07252-2

**Published:** 2017-08-04

**Authors:** Xing Guo, Chin Cheung Tang, Daniel C. Thomas, Thomas L. P. Couvreur, Richard M. K. Saunders

**Affiliations:** 1 School of Biological Sciences, The University of Hong Kong, Hong Kong, China; 2 Singapore Botanic Gardens, 1 Cluny Road, Singapore, 259569 Singapore; 3 Institut de Recherche pour le Développement (IRD), UMR-DIADE, BP 64501, F-34394 Montpellier, cedex 5 France; 4 School of Science and Technology, The Open University of Hong Kong, Ho Man Tin, Kowloon, Hong Kong, China

## Abstract

The Annonaceae, the largest family in the early-divergent order Magnoliales, comprises 107 genera and c. 2,400 species. Previous molecular phylogenetic studies targeting different taxa have generated large quantities of partially overlapping DNA sequence data for many species, although a large-scale phylogeny based on the maximum number of representatives has never been reconstructed. We use a supermatrix of eight chloroplast markers (*rbcL*, *matK*, *ndhF*, *psbA-trnH*, *trnL-F*, *atpB-rbcL*, *trnS-G* and *ycf1*) to reconstruct the most comprehensive tree to date, including 705 species (29%) from 105 genera (98%). This provides novel insights into the relationships of five enigmatic genera (*Bocagea*, *Boutiquea*, *Cardiopetalum*, *Duckeanthus* and *Phoenicanthus*). Fifteen main clades are retrieved in subfamilies Annonoideae and Malmeoideae collectively, 14 of which correspond with currently recognised tribes. *Phoenicanthus* cannot be accommodated in any existing tribe, however: it is retrieved as sister to a clade comprising the tribes Dendrokingstonieae, Monocarpieae and Miliuseae, and we therefore validate a new tribe, Phoenicantheae. Our results provide strong support for many previously recognised groups, but also indicate non-monophyly of several genera (*Desmopsis*, *Friesodielsia*, *Klarobelia*, *Oxandra*, *Piptostigma* and *Stenanona*). The relationships of these non-monophyletic genera—and two other genera (*Froesiodendron* and *Melodorum*) not yet sampled—are discussed, with recommendations for future research.

## Introduction

The Annonaceae are a pantropical family of flowering trees, shrubs and lianas, predominantly growing in tropical and subtropical lowland forests. It is the most species-rich family amongst the early-divergent magnoliids^1^, with 107 genera and c. 2,400 species currently recognised (Table 1). Previous molecular phylogenetic analyses^2–5^ have clarified backbone relationships within the family and hence contributed towards a framework for addressing higher-level infrafamilial taxonomy. A recently published phylogenetic study based on eight plastid markers and representatives of 94 genera^2^

**Table 1:** List of currently recognised genera of Annonaceae.

**Subfam. Anaxagoreoideae**	Tribe Monodoreae (86 spp.)	* Malmea* (7 spp.)
* Anaxagorea* (30 spp.)	* Asteranthe* (3 spp.)	* Mosannona* (14 spp.)
	* Hexalobus* (5 spp.)	* Onychopetalum* (2 spp.)
**Subfam. Ambavioideae** (56 spp.)	* Isolona* (20 spp.)	* Oxandra* (27 spp.)^67^
* Ambavia* (2 spp.)	* Mischogyne* (2 spp.)	* Pseudephedranthus* (1 sp.)
* Cananga* (2 spp.)	* Monocyclanthus* (1 sp.)	* Pseudomalmea* (4 spp.)
* Cleistopholis* (4 spp.)	* Monodora* (14 spp.)	* Pseudoxandra* (24 spp.)
* Cyathocalyx* (9 spp.)	* Ophrypetalum* (1 sp.)	* Ruizodendron* (1 sp.)
* Drepananthus* (26 spp.)	* Sanrafaelia* (1 sp.)	* Unonopsis* (48 spp.)
* Lettowianthus* (1 sp.)	* Uvariastrum* (5 spp.)^65^	Tribe Maasieae
* Meiocarpidium* (1 sp.)	* Uvariodendron* (15 spp.)	* Maasia* (6 spp.)
* Mezzettia* (4 spp.)	* Uvariopsis* (19 spp.)^66^	Tribe Fenerivieae
* Tetrameranthus* (7 spp.)	Tribe Uvarieae (474 spp.)	* Fenerivia* (10 spp.)
	* Afroguatteria* (3 spp.)^7^	Tribe Phoenicantheae
**Subfam. Annonoideae** (1,515 spp.)	* Cleistochlamys* (1 sp.)	* Phoenicanthus* (2 spp.)
Tribe Bocageeae (62 spp.)	* Dasymaschalon* (27 spp.)^7^	Tribe Dendrokingstonieae
* Bocagea* (2 spp.)	* Desmos* (22 spp.)^7^	* Dendrokingstonia* (3 spp.)
* Cardiopetalum* (3 spp.)	* Dielsiothamnus* (1 sp.)	Tribe Monocarpieae
* Cymbopetalum* (27 spp.)	* Fissistigma* (59 spp.)	* Monocarpia* (4 spp.)
* Froesiodendron* (3 spp.)*	* Friesodielsia* (38 spp.)^7^	Tribe Miliuseae (556 spp.)
* Hornschuchia* (10 spp.)	* Melodorum* (11 spp.)*	* Alphonsea* (29 spp.)
* Mkilua* (1 sp.)	* Mitrella* (9 spp.)	* Desmopsis* (14 spp.)
* Porcelia* (8 spp.)	* Monanthotaxis* (94 spp.)^7^ ^,a^	* Huberantha* (27 spp.)^9^
* Trigynaea* (8 spp.)	* Pyramidanthe* (1 sp.)	* Marsypopetalum* (6 spp.)
Tribe Guatterieae	* Schefferomitra* (1 sp.)	* Meiogyne* (26 spp.)^10, 68^ ^,b^
* Guatteria* (177 spp.)	* Sphaerocoryne* (4 spp.)^7^	* Miliusa* (60 spp.)^69^
Tribe Xylopieae (269 spp.)	* Toussaintia* (4 spp.)	* Mitrephora* (49 spp.)
* Artabotrys* (105 spp.)	* Uvaria* (199 spp.)	* Monoon* (60 spp.)^12^ ^,c^
* Xylopia* (164 spp.)		* Neo-uvaria* (7 spp.)^70^
Tribe Duguetieae (101 spp.)	**Subfam. Malmeoideae** (783 spp.)	* Orophea* (57 spp.)
* Duckeanthus* (1 sp.)	Tribe Piptostigmateae (35 spp.)	* Phaeanthus* (8 spp.)
* Duguetia* (94 spp.)	* Annickia* (8 spp.)	* Platymitra* (2 spp.)
* Fusaea* (2 spp.)	* Greenwayodendron* (2 spp.)	* Polyalthia* (86 spp.)^12,*d*^
* Letestudoxa* (3 spp.)	* Mwasumbia* (1 sp.)	* Popowia* (29 spp.)
* Pseudartabotrys* (1 sp.)	* Piptostigma* (13 spp.)^39^	* Pseuduvaria* (54 spp.)
Tribe Annoneae (345 spp.)	* Brieya* (2 sp.)^39^	* Sageraea* (9 spp.)
* Annona* (170 spp.)	* Polyceratocarpus* (8 spp.)	* Sapranthus* (6 spp.)
* Anonidium* (5 spp.)	* Sirdavidia* (1 sp.)^11^	* telechocarpus* (2 spp.)
* Asimina* (17 spp.)	Tribe Malmeeae (180 spp.)	* Stenanona* (14 spp.)
* Diclinanona* (3 spp.)	* Bocageopsis* (4 spp.)	* Tridimeris* (2 spp.)^71^
* Disepalum* (9 spp.)	* Cremastosperma* (29 spp.)	* Trivalvaria* (5 spp.)
* Goniothalamus* (134 spp.)	* Ephedranthus* (7 spp.)	* Wangia* (2 spp.)^72^
* Neostenanthera* (6 spp.)	* Klarobelia* (12 spp.)	* Winitia* (2 spp.)^14^

**Figure 1:**
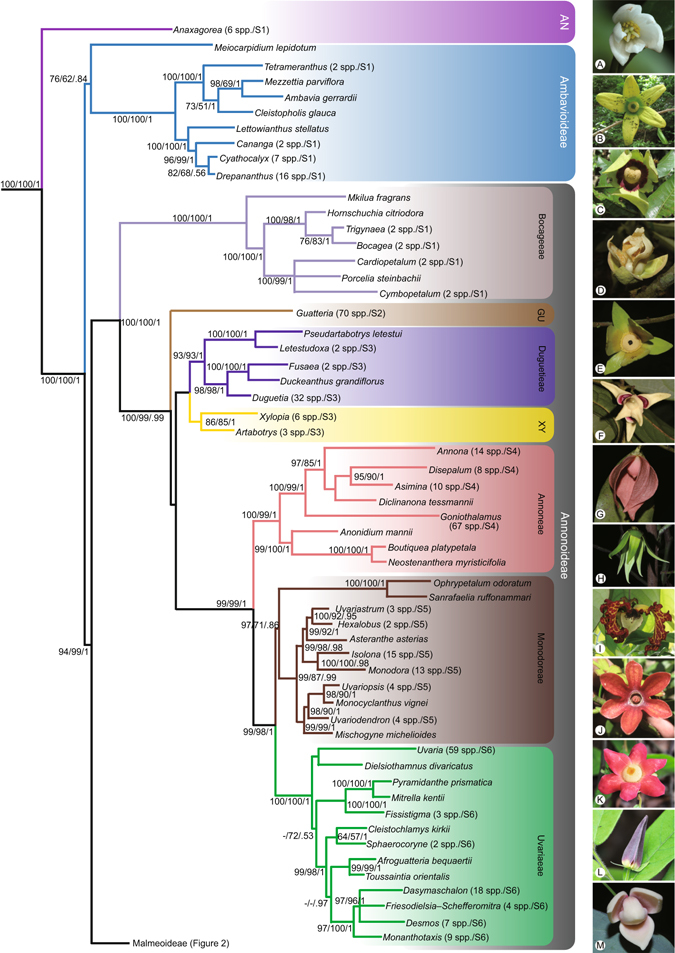
Skeletal representation of the best-scoring maximum likelihood tree inferred from a 754-accession dataset of eight chloroplast markers, showing topology of Anaxagoreoideae, Ambavioideae and Annonoideae. Tips represent genera or subdivisions when genera are not monophyletic. Higher taxon names appear to the right, marked with different background colours. ML bootstrap (BS) values ≥50, MP jackknife (JK) values ≥50 and Bayesian posterior probabilities (PP) values ≥0.5 are indicated at each node: BS /JK/ PP. -, represents clade support values <50%. Numbers in brackets show numbers of sampled taxon and supplementary figures of each lineage. Subfamily and tribe abbreviations: AN: Anaxagoreoideae; GU: Guatterieae; XY: Xylopieae. Flower morphology of the selected genera: (**A**) *Anaxagorea luzonensis*; (**B**) *Lettowianthus stellatus*; (**C**) *Mkilua fragrans*; (**D**) *Duguetia confinis*; (**E**) *Fusaea longifolia*; (**F**) *Artabotrys hongkongensis*; (**G**) *Goniothalamus repevensis*; (**H**) *Anonidium floribundum*; (**I**) *Monodora myristica*; (**J**) *Isolona hexaloba*; (**K**) *Uvaria grandiflora*; (**L**) *Dasymaschalon trichophorum*; (**M**) *Sphaerocoryne gracilipes*. — Photographs: A, Chun Chiu Pang; B–E, H–J, M, Thomas L. P. Couvreur; F, Junhao Chen; G, L, K, Xing Guo; Photos available at the World Annonaceae website^64^: http://annonaceae.myspecies.info/.

**Figure 2:**
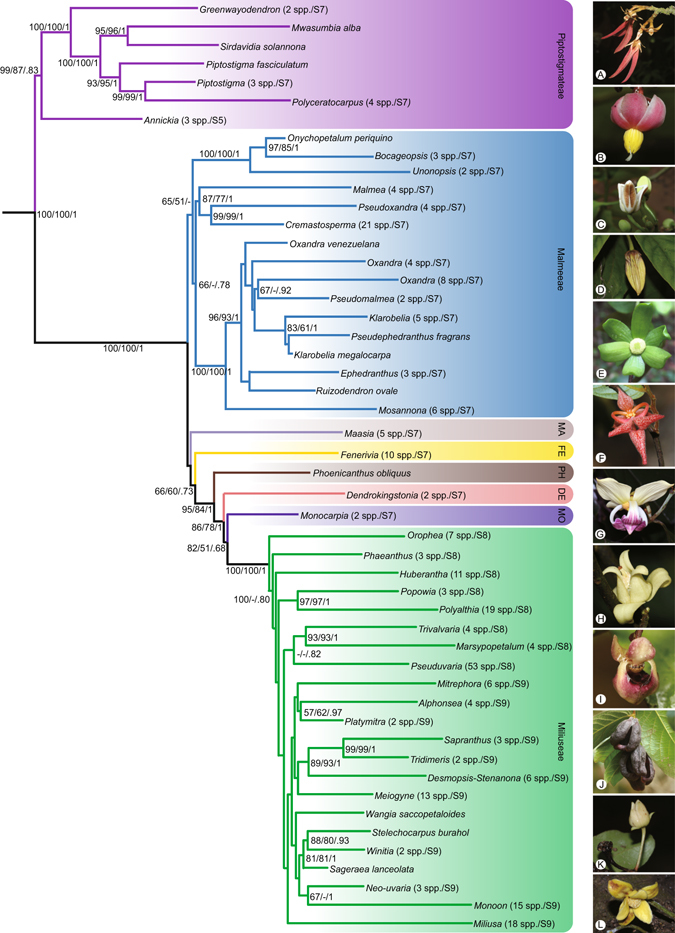
Skeletal representation of the best-scoring maximum likelihood tree inferred from a 754-accession dataset of eight chloroplast markers, showing topology of Malmeoideae. Tips represent genera or subdivisions when genera are not monophyletic. Higher taxon names appear at right, marked with different background colours. ML bootstrap (BS) values ≥50, MP jackknife (JK) values ≥50 and Bayesian posterior probabilities (PP) values ≥0.5 are indicated at each node: BS /JK/ PP. -, represents clade support values <50%. Numbers in brackets show numbers of sampled taxon and supplementary figures of each lineage. Tribe abbreviations: MA: Maasieae; FE: Fenerivieae; PH: Phoenicantheae; DE: Dendrokingstonieae; MO: Monocarpieae. Flower morphology of the selected genera: (**A**) *Piptostigma multinervium*; (**B**) *Sirdavidia solannona*; (**C**) *Onychopetalum periquino*; (**D**) *Phaeanthus ophthalmicus*; (**E**) *Huberantha tanganyikensis*; (**F**) *Orophea maculata*; (**G**) *Mitrephora winitii*; (**H**) *Alphosea javanica*; (**I**) *Pseuduvaria froggattii*; (**J**) *Meiogyne heteropetala*; (**K**) *Polyalthia suberosa*; (**L**) *Stelechocarpus burahol*. — Photographs: A–C, E, Thomas L. P. Couvreur; F, Junhao Chen; D, G, H, L, Xing Guo; I, J, Chun Chiu Pang; K, Daniel C. Thomas. Photos available at the World Annonaceae website^64^: http://annonaceae.myspecies.info/.

Unless indicated otherwise, number of recognised species is based on AnnonBase^64^. Subfamilies and tribes arranged according to the phylogenetic trees (Figs 1, 2); genera listed alphabetically within tribes. *Genera not included in the phylogenetic analyses presented here. ^a^Current delimitation includes *Exellia*, *Gilbertiella* and African *Friesodielsia* ^7^. ^b^Current delimitation includes *Fitzalania*, *Oncodostigma* and some *Polyalthia* species from Fiji^10^. ^c^Current delimitation includes *Enicosanthum* and *Woodiellantha* ^12^. ^d^Current delimitation includes *Haplostichanthus* ^12^.

was used to formally classify the Annonaceae into four subfamilies, Anaxagoreoideae, Ambavioideae, Annonoideae and Malmeoideae, and further subdivide these subfamilies into 14 tribes.

Since the publication of the family-wide phylogeny of Annonaceae^2^, several other molecular (and combined molecular-morphological) studies have made important contributions to our understanding of phylogenetic relationships and generic circumscriptions in various lineages, including: *Disepalum* ^6^, *Friesodielsia*-*Monanthotaxis* ^7^, *Goniothalamus* ^8^, *Huberantha* (as ‘*Hubera*’)^9^, *Meiogyne* ^10^, tribe Piptostigmateae^11^, *Polyalthia*-*Monoon* ^12^, *Wangia* ^13^ and *Winitia* ^14^. Despite these significant advances over the past decade, several genera (*Bocagea*, *Boutiquea*, *Cardiopetalum*, *Duckeanthus*, *Froesiodendron*, *Melodorum* and *Phoenicanthus*) remain unsampled due to difficulties in obtaining DNA of sufficient quality for phylogenetic reconstruction, and hence their systematic placements within the family remain unknown.

A robust phylogenetic framework based on a maximum number of Annonaceae representatives is invaluable for understanding the diversity, classification and evolution of the family. Numerous recently published phylogenetic studies have focused on specific genera and hence have incorporated differing taxon sampling; these studies have generated large quantities of partially overlapping sequence data, providing an opportunity for a wider family-level analysis addressing some gaps in our current knowledge of phylogenetic relationships.

In this study, we reconstruct the phylogeny of the Annonaceae based on a supermatrix of eight chloroplast loci and 749 accessions representing 705 species (29% of c. 2,400 currently recognised species) of 105 genera (98% of 107 currently accepted genera). The data matrix includes nearly four times as many species and representatives of 15 additional genera in comparison to the largest previous study (193 spp.)^2^. The aims of this study are: (i) to reconstruct the most comprehensive evolutionary tree of life for the Annonaceae available to date, providing a robust platform for future evolutionary studies; (ii) to determine the phylogenetic position of five genera (*Bocagea*, *Boutiquea*, *Cardiopetalum*, *Duckeanthus* and *Phoenicanthus*), which were not included in any previous molecular phylogenetic reconstructions; (iii) to assess the monophyletic status and phylogenetic relationships within each major clade, highlighting possible non-monophyly of genera and evaluating alternative resolutions to nomenclatural problems; (iv) to identify and discuss additional taxonomic problems that await resolution, including the phylogenetic placement and taxonomy of two genera, *Froesiodendron* and *Melodorum*, which have not been sampled yet; and (v) to provide an updated overview of currently recognised genera in the family (Table 1) with their species richness.

## Results

The concatenated alignment for the dataset with 754 terminals consisted of 10,782 positions. The characteristics and best-fitting nucleotide substitution model for each data matrix are presented in Table 2. A summary of the best-scoring maximum likelihood (ML) tree showing the phylogenetic backbone of the Annonaceae is presented in Figs 1 and 2, with tips representing genera (or subdivisions of genera when not monophyletic). The entire tree with all 754 terminals is presented as Supplementary Figs S1–S9. An updated list of currently recognised genera in the Annonaceae is given in Table 1, with 107 genera and c. 2,400 species.

**Table 2:** Descriptive statistics and best-fitting substitution model for each of the eight chloroplast regions and the concatenated datasets.

Matrix	Terminals	Characters analysed	Variable characters (%)	Parsimony-informative characters (%)	CI	RI	AIC model selection
*atpB-rbcL*	177	1161	495 (42.6%)	328 (28.3%)	0.63	0.92	GTR + Γ
*matK*	648	837	540 (64.5%)	495 (59.1%)	0.47	0.94	GTR + I + Γ
*ndhF*	282	2102	934 (44.4%)	1201 (57.1%)	0.39	0.88	GTR + I + Γ
*psbA-trnH*	591	444	208 (46.8%)	275 (61.9%)	0.41	0.93	GTR + I + Γ
*rbcL*	633	1346	334 (24.8%)	504 (37.4%)	0.35	0.91	GTR + Γ
*trnL-F*	722	1272	550 (43.2%)	724 (56.9%)	0.48	0.93	GTR + Γ
*trnS-G*	165	1470	384 (26.1%)	721 (49%)	0.65	0.89	GTR + Γ
*ycf1*	132	2150	876 (40.7%)	1170 (54.4%)	0.74	0.96	GTR + Γ
Combined data	754	10782	4030 (37.4%)	5631 (52.2%)	0.47	0.91	GTR + I + Γ

CI = ensemble consistency index; RI = ensemble retention index.

For the Bayesian inference (BI) analysis, the partitioned analyses based on region identity provided distinctly better explanations of the data than both analyses using the non-partitioned model and analyses of the two-partitioned dataset: 2lnB (8-partitioned over non-partitioned) = 2,493; and 2lnB (8-partitioned over 2-partitioned) = 947, significantly above the threshold value of 10. The posterior probability (PP) values derived from the analyses using eight partitions were therefore selected as the results of the Bayesian analyses.

The maximum parsimony (MP), ML, and BI analyses yielded similar topologies, differing mainly in the relative jackknife (JK), bootstrap (BS) and PP values, respectively, for particular groups (Figs 1 and 2; Supplementary Figs S1–S9). Our inferred phylogeny is generally consistent with previous phylogenetic analyses of the family, although we clarify the relationships of five genera for the first time, viz.: (1) *Bocagea*, sister to *Trigynaea* (tribe Bocageeae) (BS = 76; JK = 83; PP = 1); (2) *Cardiopetalum*, located in the clade together with *Cymbopetalum* and *Porcelia* (tribe Bocageeae) (BS = 100; JK = 99; PP = 1); (3) *Boutiquea*, sister to *Neostenanthera* (tribe Annoneae) (BS = 100; JK = 100; PP = 1); (4) *Duckeanthus*, sister to *Fusaea* (tribe Duguetieae) (BS = 100; JK = 100; PP = 1); and (5) *Phoenicanthus*, which does not belong to any currently recognised tribe, as sister to a clade comprising the tribes Dendrokingstonieae, Monocarpieae and Miliuseae (BS = 95; JK = 83; PP = 1).

The monophyletic status of the majority of the 105 sampled genera is supported in all analyses (Figs 1 and 2; Supplementary Figs S1–S9). Six genera, however, are consistently retrieved as non-monophyletic: *Desmopsis* (Fig. S9), *Friesodielsia* (Fig. S6), *Klarobelia* (Fig. S7), *Oxandra* (Fig. S7), *Piptostigma* (Fig. S7) and *Stenanona* (Fig. S9).

## Discussion

Our phylogenetic reconstructions consistently retrieved 17 primary clades, which are grouped into four moderately to highly supported larger clades (Fig. 1; Supplementary Fig. S1). There is considerable congruence between these clades and the four subfamilies and 14 tribes currently recognised^2^. Since the phylogeny and taxonomy of these clades were discussed extensively by Chatrou *et al*.^2^ the relationships between them will not be discussed again here. We focus instead on the phylogenetic placement of the five newly sampled genera and the non-monophyletic status of several genera indicated by our large-scale phylogenetic reconstruction.

### Phylogenetic relationships of previously unplaced genera

The monotypic genus *Boutiquea*, essentially endemic to Cameroon, is resolved within the tribe Annoneae, unambiguously supported as sister to the genus *Neostenanthera*, which consists of five species from West and Central Africa^15^ (Fig. 1; Supplementary Fig. S4). *Boutiquea* was already included in the tribe Annoneae but only based on pollen and morphological characters^2^. This sister relationship is consistent with floral and pollen morphology: both genera have an elevated floral torus, very short sepals, elongated petals, three apically connivent inner petals that form a mitriform dome over the reproductive organs^16^, and pollen grains with a granular infratectum that are released as tetrads^17, 18^. In addition, both genera have septate anthers in which the sporogenous cells are partitioned by transverse or longitudinal walls comprising sterile tissue^19^. Interestingly, *Boutiquea platypetala* (Engl. & Diels) Le Thomas was previously included in *Neostenanthera*, although Le Thomas^20^ noted that it was distinguished from other species in the genus by its sessile monocarps, which are divided into a conical apical and a hemispheric basal region by a thickened transverse ledge at the widest part, hence differing from the clearly stipitate and fusiform or ellipsoid monocarps typical of *Neostenanthera* ^15^. Given the morphological similarities between *Boutiquea platypetala* and *Neostenanthera* and the sister-group relationship between the two genera, we suggest that there is little to be gained from their continued separation. Since the generic name *Neostenanthera* ^21^ antedates that of *Boutiquea* ^20^, we recommend that the latter name should be treated as a synonym of *Neostenanthera*, and that the existing combination *Neostenanthera platypetala* (Engl. & Diels) Pellegr. be adopted.

Our phylogenetic results suggest that the Brazilian monotypic genus *Duckeanthus* is sister to *Fusaea* (BS = 100; JK = 100; PP = 1, Fig. 1; Supplementary Fig. S3) within the tribe Duguetieae; this corroborates the results of previous cladistic analyses based on morphological characters^22^, which indicated that *Duckeanthus* and *Fusaea* have similar inflorescences, stamen anatomy and aril structure. The two genera are also palynologically similar, with large pollen tetrads with a minutely granular exine structure^18, 23, 24^. *Fusaea* differs from *Duckeanthus*, however, in its fused carpels, a conspicuous ring of staminodes, and the fused calyx^16^.

The Neotropical genera *Bocagea* and *Cardiopetalum* are confirmed as members of the tribe Bocageeae (Fig. 1; Supplementary Fig. S1). *Bocagea* is shown to be sister to *Trigynaea*, and *Cardiopetalum* is retrieved in a clade together with *Cymbopetalum* and *Porcelia*. These genera, together with *Hornschuchia* and *Mkilua*, are shown to be collectively monophyletic and morphologically easily distinguished from other genera in the family by reference to their solitary internodal ebracteate pedicels that are basally articulated, and pollen that is shed in polyads comprising eight or more grains^25^. Apart from the African genus *Mkilua*, which forms the basal lineage, all other genera of this tribe are Neotropical, forming two well supported subclades, *Cardiopetalum*-*Cymbopetalum*-*Porcelia* (BS = 100; JK = 99; PP = 1) and *Bocagea*-*Hornschuchia*-*Trigynaea* (BS = 100; JK = 98; PP = 1). These inferred relationships are consistent with the previous cladogram based on morphological characters^25^: the *Cardiopetalum*-*Cymbopetalum*-*Porcelia* clade is supported by outer petals with valvate to slightly imbricate aestivation, the presence of specialised tissues on petal margins that function as pollinator food rewards, and the absence of a seed caruncle; the *Bocagea*-*Hornschuchia*-*Trigynaea* clade, in contrast, is united by a suite of eight characters, including the narrow floral torus, sepals that are persistent in the fruit, inner petals with a trigonous apex, few stamens, elongate anther connectives, variably tectate pollen exine, and non-articulated stigmas.

*Phoenicanthus* is one of the most poorly known genera in the Annonaceae, with only two species currently recognised. The genus has been placed in tribe Miliuseae in previous classifications^2, 26, 27^ because of their ‘miliusoid’ stamens (in which the connective does not extend over the thecae) with an obtuse apex. Miliusoid stamens have been shown to be morphologically highly variable, however, and likely homoplasious in both the Miliuseae and the wider family^16, 28^. The phylogenetic results presented here, however, indicate that *Phoenicanthus* is the next-divergent branch subsequent to the tribe Fenerivieae, and strongly supported as sister to a clade comprising the tribes Dendrokingstonieae, Monocarpieae and Miliuseae (BS = 95; JK = 83; PP = 1, Fig. 2; Supplementary Fig. S7): the tribe Miliuseae is shown as sister to the Monocarpieae, with these two tribes collectively sister to the Dendrokingstonieae. The inferred relationships within this clade clearly contradict any association of *Phoenicanthus* with the tribe Miliuseae.

*Phoenicanthus* can easily be distinguished from *Fenerivia* by the lack of a prominent flange immediately below the perianth, which has been interpreted as the highly reduced calyx and synapomorphic for *Fenerivia* ^29, 30^. Similarities between *Phoenicanthus*, *Dendrokingstonia* and *Monocarpia* include their eucamptodromous leaf venation with percurrent tertiary veins (X. Guo, pers. observ.) and very limited number of carpels (only 1–3 per flower)^31^. *Phoenicanthus* differs from the other two genera, however, by a combination of macromorphological flower and fruit characters: *Dendrokingstonia* and *Monocarpia* have considerably enlarged peltate stigmas, whorled stamens, and relatively large monocarps (3–5 cm in diameter); *Phoenicanthus*, in contrast, has reduced stigmas, stamens arranged in a triangular floral meristem with solitary stamens at the corners, and small monocarps (c. 1 cm in diameter).

Our molecular phylogenetic analyses indicate that *Phoenicanthus* cannot be accommodated in any of the existing tribes. This is supported by the morphological data, which provide strong support for distinguishing *Phoenicanthus* from related taxa. A new tribe is accordingly warranted, and is described below as tribe Phoenicantheae:

**Phoenicantheae** X. Guo & R. M. K. Saunders, **tribus nov**. – TYPE GENUS: *Phoenicanthus* Alston in Trimen, Handb. Fl. Ceylon 6: 6 (1931).

Glabrous trees; pedicel bracts present; flowers bisexual, solitary or in few-flowered fascicles, terminal; sepals 3 per flower, slightly connate; petals 6 per flower, subequal, inner petals mitriform and concave at the base; stamens 6 or 9 per flower, ‘miliusoid’ stamens (connectives not extending over thecae) with obtuse apex, triangularly arranged; carpel(s) 1–3 per flower, stigmas reduced; ovule(s) 1–2 per carpel; monocarps globose, sessile.

Comprising a single genus, *Phoenicanthus*, with two species, *P. coriacea* (Thwaites) H. Huber and *P. obliquus* (Hook. f. & Thomson) Alston, endemic to Sri Lanka^32^.

### Polyphyletic and paraphyletic genera

Our phylogenetic reconstructions support the monophyletic status of the majority of genera, but six genera are not well supported as monophyletic, viz. *Desmopsis*, *Friesodielsia*, *Klarobelia*, *Oxandra*, *Piptostigma* and *Stenanona*. The large-scale phylogeny presented here provides an overview of all non-monophyletic genera, although relevant relationships have previously been reported in phylogenetic analyses targeting specific genera^2, 4, 7, 11, 33–35^.

*Klarobelia* (tribe Malmeeae: Fig. 2; Supplementary Fig. S7) is shown to be paraphyletic, with the monotypic genus *Pseudephedranthus* nested within it (BS = 83; JK = 61; PP = 1), corroborating the results of previous phylogenetic analyses^2, 4^. The problem is further complicated because the *Klarobelia-Pseudephedranthus* clade, together with *Pseudomalmea*, are deeply nested within *Oxandra*, which is itself paraphyletic (Fig. 2; Supplementary Fig. S7). The non-monophyletic status of *Oxandra* has been reported in previous phylogenetic studies^2, 5^, highlighting the problem in the current generic circumscription.

*Klarobelia* and *Pseudomalmea* species were originally classified in *Malmea* ^36^ but were subsequently removed and accommodated in two newly described genera^37^ on the basis of leaf, inflorescence and seed characters. In general appearance, *Klarobelia* and *Pseudomalmea* are very similar to *Oxandra*, although with relatively minor differences in the number of bracts per pedicel, petal shape, and monocarp stipe length^38^: *Oxandra* species have 3–6 bracts per pedicel, petals that are 4–8 mm long, and stipes shorter than 10 mm; whereas *Klarobelia* and *Pseudomalmea* species have only one or two bracts per pedicel, petals that are 7–70 mm long, and stipes longer than 10 mm. *Klarobelia* only differs from *Pseudomalmea* by its concave petals that cover the reproductive organs, which are outwardly spreading in *Pseudomalmea*. *Pseudephedranthus* also closely resembles *Oxandra*, with differences restricted to its longer petals and discoid stamen apices^16^.

One possible treatment to render *Oxandra* monophyletic might be to adopt a broad generic delimitation by merging *Klarobelia*, *Pseudomalmea* and *Pseudephedranthus* into *Oxandra*. Alternatively, *Oxandra* could be treated in a narrow sense including species located in the same clade with the type species *O*. *lanceolata*, with the remaining distantly related species transferred to other genera or segregated as a new genus. Most nodes within this clade are poorly supported (Fig. 2; Supplementary Fig. S7), however, probably due to the limited number of DNA regions sequenced (only *rbcL*, *psbA-trnH* and *trnL-F* are available for most species). Further studies with a more extensive taxon sampling and based on additional DNA regions are essential before validating nomenclatural changes for these genera.

The genus *Piptostigma* is shown to be paraphyletic, with *P. fasciculatum* (De Wild.) Boutique ex Fries sister to a well-supported clade (BS = 99; JK = 99; PP = 1) comprising three species of *Piptostigma* and four species of *Polyceratocarpus* (Fig. 2; Supplementary Fig. S7). These relationships are consistent with those of previous phylogenetic studies^11, 35, 39^. Morphological data also indicate that *Piptostigma* is heterogeneous, with the majority of species possessing tuberculate monocarps and sepaloid outer petals, whereas *P. fasciculatum* has relatively smooth monocarps and outer petals that are similar to the inner petals. Based on combined molecular and morphological data, Ghogue *et al*.^39^ have recently removed *P. fasciculatum* from *Piptostigma*, transferring it to the resurrected genus *Brieya* which now contains two species (*Brieya fasciculata* De Wild. and *Brieya latipetala* Exell).

The two species of *Stenanona* (tribe Miliuseae) sampled in the present study are retrieved in two separate lineages nested within *Desmopsis*, rendering the latter genus polyphyletic (Fig. 2; Supplementary Fig. S9). The relationships inferred here are partially congruent with those of a previous study^35^ based on two DNA regions (*rbcL* and *trnL-F*), in which two sampled species of *Desmopsis* formed a clade together with species of *Stenanona* and *Stelechocarpus*. Non-monophyly of *Desmopsis* and *Stenanona* was also confirmed in a recent phylogenetic study by Ortiz-Rodriguez *et al*.^34^, which was based on a more extensive taxon sampling with ten *Stenanona* spp. and eight *Desmopsis* spp. Despite the likely congeneric status of *Desmopsis* and *Stenanona*, Ortiz-Rodriguez *et al*. refrained from formalising any new combinations because relationships within the *Desmopsis-Stenanona* clade have not been fully resolved yet and several species of *Desmopsis* have not been formally described. Ortiz-Rodriguez *et al*.^34^ also proposed that the Neotropical clade within the Miliuseae be recognised as the subtribe Sapranthinae. Recognition of a single subtribe raises problems with the classification of the other genera in a complementary but potentially non-monophyletic subtribe: backbone relationships in the tribe are poorly resolved^2, 5, 12, 29^, and we believe that a subtribal classification is premature at best.

The monotypic genus *Schefferomitra* was recently shown to be nested within the Asian genus *Friesodielsia* (BS = 99; JK = 100; PP = 1), with African species that were previously placed under the latter name transferred to *Afroguatteria*, *Monanthotaxis* and *Sphaerocoryne* ^7^. Detailed examination of the morphological characters of the two lineages supported the phylogenetic relationship and suggested that there are no convincing criteria to support the continued recognition of *Friesodielsia* and *Schefferomitra* as distinct genera. Although the latter name has nomenclatural priority, Guo *et al*.^40^ proposed conservation of the name *Friesodielsia* in order to promote nomenclatural stability; no formal nomenclatural change has been made, however, pending the decision by the Nomenclature Committee for Vascular Plants. In addition to addressing the polyphyletic status of *Friesodielsia*, Guo *et al*.^7^ also amended the generic delimitation of *Monanthotaxis* by including the former genera *Exellia* and *Gilbertiella*.

### Intraspecific non-monophyly in *Guatteria*

*Guatteria* is a large Neotropical genus with 177 species currently recognised^41^. The phylogenetic relationships within the genus retrieved here (Fig. S2) are largely congruent with those published previously^42^. Multiple accessions of more than 10 species do not form a well-supported clade, however. The non-monophyly of some species (e.g. *G*. *amplifolia*, *G*. *hirsuta* and *G*. *punctata*) is strongly supported: two accessions of *G*. *amplifolia*, for example, are retrieved as sister to *G*. *latifolia* with strong Bayesian support, whilst a third accession is shown to be more closely related to *G*. *jefensis* (BS = 86; JK = 85; PP = 1). The non-monophyly of these species may be the result of misidentification as they belong to several species complexes with problematic species delimitations^41^.

Other conflicts lack statistical support, however. The few cpDNA regions used (only *matK*, *psbA-trnH*, *rbcL* and *trnL-F* have been sequenced for the majority of species) contain limited phylogenetic information, resulting in poor resolution in this part of the tree. Additional unlinked data from different genomes are necessary to improve resolution, identifying potential gene tree incongruence and differentiate likely underlying biological causes such as incomplete lineage sorting, introgression, and/or unrecognised paralogy.

### Genera not sampled

Although we initially planned to achieve a comprehensive sampling of all genera in the Annonaceae, PCR reactions of *Froesiodendron* were unsuccessful due to the poor quality of available leaf material. *Froesiodendron* comprises three species from tropical South America, and has been inferred to belong to the tribe Bocageeae based on its solitary internodal ebracteate pedicels, septate stamens and pollen shed as polyads^2, 16, 25^. Morphological cladistic analyses^25^ furthermore suggest that *Froesiodendron* is more closely related to *Cardiopetalum*, *Cymbopetalum* and *Porcelia* than to other genera in the tribe. These four genera are united by outer petals that show valvate to slightly imbricate aestivation and with specialised beetle-feeding tissues on the petal margins.

A molecular phylogenetic re-evaluation of the circumscription of *Uvaria* ^43^ led to several satellite genera being subsumed and many species transferred to an expanded *Uvaria*. Although all Australian representatives of *Melodorum* were transferred to *Uvaria* in this process, 11 species remained; the generic name *Melodorum* was not synonymised with *Uvaria* due to typification problems, with *Melodorum* long confused with *Sphaerocoryne*: the former name has been incorrectly applied to species belonging to the latter^27, 44^. It seems likely that the name of the type species, *M. fruticosum* Lour., has been widely misapplied in many published phylogenies and that the specimen used may represent a species of *Sphaerocoryne* ^43^. Further molecular and morphological studies with more extensive taxon sampling and a re-evaluation of nomenclatural type specimens are required to clarify the nomenclatural problems associated with the application of the name *Melodorum*.

### Supermatrix and large-scale phylogenetic reconstruction

Results from our supermatrix analyses are promising. Even though the concatenated matrix had c. 55% missing data, we found that the generic and tribal positions of most species were consistent with previous taxonomic research, and often very strongly supported. We compared the results of our tree searches with those from the next-largest available phylogeny for the family (based on a 193-species dataset)^2^, with nodes excluded within the poorly resolved tribe Miliuseae (Supplementary Table S1). Of the total 25 major nodes that differed between two analyses, all but three of the nodes of our analyses are better resolved and/or have better support values than in the previous 193-species phylogeny^2^. This improved phylogenetic performance suggests that the strategy employed in this study of maximising the number of loci and taxa has greater power for resolving relationships, particularly at deeper nodes in the phylogeny, than traditional approaches in which only one or two species of each genus are included as placeholders, despite highly incomplete alignment.

Supermatrix methods offer a variety of advantages, including the ability to reconstruct more inclusive phylogenies at broad scales with minimal investment in sequencing^45, 46^. These methods present their own challenges, however, including issues of sparse alignment, data integrity, computational power and time efficiency^47^. Our sampling criterion has largely overcome these problems, however, and greatly facilitated the integration of the different DNA regions used in this study. Specifically, we excluded accessions with data for fewer than three regions available. In the concatenated dataset, 96% accessions have *trnL-F* sequence data, 86% have *matK* data, and 84% have *rbcL* data. Thus, most species have comparable data for at least three regions, which may have greatly facilitated tree reconstruction despite lacking other regions. Importantly, our study also provides a framework to which additional sequences can readily be added in future research. We anticipate that this large-scale phylogeny will be of broad utility for many areas of Annonaceae research, including historical biogeography, diversification rate studies and ecology.

## Materials and Methods

### Taxon and DNA region sampling

We adopted a supermatrix approach, integrating available data for 858 Annonaceae accessions downloaded from the nucleotide database of National Center for Biotechnology Information (http://www.ncbi.nlm.nih.gov). *Drepananthus longiflorus*, *Hexalobus monopetalus*, *Melodorum fruticosum*, *Onychopetalum amazonicum*, *Unonopsis elegantissima*, *Unonopsis perrottetii* and *Unonopsis rufescens* were excluded due to ambiguous identifications, and *Monoon borneense*, *Pseudoxandra bahiensis* and *Pseudoxandra cuspidata* were excluded due to hard incongruence between DNA markers. An additional 107 accessions were excluded for one or a combination of the following reasons: (1) multiple accessions of a single species (except for *Guatteria* which shows problems of intraspecific non-monophyly); (2) specimen only identified to genus level; or (3) data for fewer than three DNA regions available. Exceptions were made regarding the latter criterion for certain accessions if genera were represented by fewer than three accessions in total. Additionally, data for eight accessions representing five genera (*Bocagea*, *Boutiquea*, *Cardiopetalum*, *Duckeanthus* and *Phoenicanthus*) were newly generated, with voucher information provided in Supplementary Appendix I. Species belonging to three other families in the Magnoliales, including the Myristicaceae (*Myristica fragrans* and *Coelocaryon preussii*), Magnoliaceae (*Magnolia kobus* and *Liriodendron chinense*) and Eupomatiaceae (*Eupomatia bennettii*), were selected as outgroups. The final matrix therefore comprised 749 ingroup and five outgroup accessions (see Appendix I for voucher information), representing c. 98% of generic diversity and c. 29% of species diversity in the Annonaceae.

DNA sequences of eight chloroplast regions (*rbcL*, *matK*, *ndhF*, *psbA-trnH*, *trnL-F*, *atpB-rbcL*, *trnS-G* and *ycf1*), which are commonly used in Annonaceae phylogenetics, were downloaded from the nucleotide database of the National Center for Biotechnology Information (http://www.ncbi.nlm.nih.gov) or generated for the newly added samples in this study. Attempts at sequencing two genera, *Melodorum* and *Froesiodendron*, were unsuccessful due to the poor quality of available leaf materials. GenBank accession number for all samples included in the analyses are given in Supplementary Appendix II.

### DNA extraction, amplification and sequencing

Total DNA was isolated from herbarium material using the innuPrep Plant DNA Kit (Analytik Jena, Jena, Germany) following the manufacturer’s instructions. Polymerase chain reaction (PCR) amplification and sequencing were performed using the same procedures as previously described^8, 48^.

### Sequence assembly, alignment and phylogenetic analyses

Sequence fragments were edited and assembled using GeneiousPro v.7.1.9 (Biomatters; http://www.geneious.com). Sequences of individual regions were subsequently aligned automatically using the MAFFT plugin^49^ in Geneious with default settings, and then manually edited and optimised. Characters in regions for which alignment was ambiguous or included inversions and short repetitive sequences were excluded from the analyses.

Phylogenetic reconstruction was performed using maximum parsimony (MP), maximum likelihood (ML), and Bayesian inference (BI) methods. DNA sequences for the individual DNA regions were concatenated rather than analysed independently as chloroplast DNA is inherited as a unit.

For the MP analyses, all characters were treated as independent and of equal weight, with gaps treated as missing data. A heuristic search was performed in PAUP* v.4.0b10^50^ with 2,000 random addition sequence replicates with TBR branch-swapping, saving 10 trees per replicate. The most parsimonious trees were summarised using a strict consensus tree. The robustness of the phylogenetic relationships was evaluated using the jackknife (JK) method^51^ with the removal probability set to approximately e^−1^ (36.7879%), and “jac” resampling emulated. 1,000 JK replicates were performed with 100 random addition tree bisection-reconnection searches (each with a maximum of 10 trees held) per replicate.

ML analyses were performed using RAxML v.8.2.6^52^ provided by the CIPRES Science Gateway^53^. The dataset was separated into eight partitions based on DNA region identity. 1,000 analyses were run from distinct random stepwise addition sequence MP starting trees under the general time-reversible nucleotide substitution model with among-site rate variation modelled with a gamma distribution (GTR + Γ). Bootstrap support (BS) values were used to estimate clade support, based on 1,000 non-parametric bootstrap replicates.

BI analysis was undertaken using MrBayes v.3.2.6^54^ with three distinct partitioning strategies: (1) non-partitioned; (2) 2-partitioned, distinguishing coding (*matK*, *ndhF*, *rbcL* and *ycf1*) and non-coding (*atpB-rbcL*, *psbA-trnH*, *trnL-F* and *trnS-G*) regions; and (3) 8-partitioned, according to DNA region identity. The appropriate DNA substitution model for each locus and concatenated matrix was determined using MrModeltest v.2.3^55^, applying the Akaike Information Criterion. For the 2-partitioned and 8-partitioned analyses, the parameter values (NST and gamma distributed rates) for each partition were allowed to evolve independently using the unlinked setting. Four Markov chain Monte Carlo (MCMC) chains were run, each beginning with a random tree and sampling one tree every 1,000 generations for 20 million generations. The mean branch length prior was reset from the default mean (0.1) to 0.01 (brlenspr = unconstrained: exponential (100.0)) to reduce the likelihood of stochastic entrapment in local tree length optima^56, 57^. Convergence was assessed using the standard deviation of split frequencies, with values <0.01 interpreted as indicating good convergence. The first 25% of samples (5,000 trees) were discarded as burn-in, and the post-burn-in samples summarised as a 50% majority-rule consensus tree.

Stationarity, convergence and a suitable effective sample size were assessed using Tracer v.1.5^58^ and were visually checked using the Cumulative and Compare functions in AWTY^59^. Inference of the analyses using the three partitioning schemes was assessed with Bayes factor comparison. The best-performing partitioning strategy was selected by applying the criterion of 2ln Bayes factor >10 as strong evidence in favour of a particular model^60, 61^.

Bootstrap/jackknife values of 50–74% were considered as weak support, 75–84% as moderate support, and 85–100% as strong support. For BI, the estimation of branch support accompanies the tree estimation and is reflected by posterior probabilities (PP)^62^; branches with PP values ≥0.95 are considered well supported, and <0.95 not supported^63^.

### Data availability

The data used in this study are available for download from the nucleotide database of the National Center for Biotechnology Information (http://www.ncbi.nlm.nih.gov). See Supplementary Appendix II for the GenBank accession numbers of all samples included in the analyses.

## Electronic supplementary material

Supplementary information 
