# Supplementary material for: A mega-phylogeny of the Annonaceae: taxonomic placement of five enigmatic genera and support for a new tribe, Phoenicantheae
Source: Sci Rep. 2017 Aug 4;7:7323. doi: 10.1038/s41598-017-07252-2 (PMC5544705; doi:10.1038/s41598-017-07252-2)
Supplement: Supplementary file 1 — Supplementary information [file 41598_2017_7252_MOESM1_ESM.pdf]

A mega-phylogeny of the Annonaceae: taxonomic placement of five enigmatic genera and support for a new tribe, Phoenicantheae

Xing Guo[1*], Chin Cheung Tang[1,2*], Daniel C. Thomas[1,3], Thomas L. P. Couvreur[4] & Richard M. K. Saunders[1]

[1]School of Biological Sciences, The University of Hong Kong, Hong Kong, China.
[2]Current address: School of Science and Technology, The Open University of Hong Kong, Ho Man Tin, Kowloon, Hong Kong, China.
[3]Singapore Botanic Gardens, 1 Cluny Road, Singapore 259569, Singapore.
[4]Institut de Recherche pour le Développement (IRD), UMR-DIADE, BP 64501, F-34394 Montpellier cedex 5, France.

[*]these authors contributed equally to this work.
Correspondence and requests for materials should be addressed to R.M.K.S. (email: saunders@hku.hk)

Keywords: Annonaceae; cpDNA, large-scale phylogeny; new tribe; taxonomy

Supplementary Table S1. Clade support of selected major nodes (above genus level) in the Annonaceae. Node numbers correlate with those shown in supplementary Figs S1–S9. *represents nodes in this study have higher support values than that of previous study[2]. Nodes within tribe Miliuseae are not compared due to the poor resolution in both analyses.

**Node**	**This study**	**193-species dataset[2]**
A: Subfam. Ambavioideae	76/62/0.84	two support values ≥ BS 85%/PP 0.95
B*: *Tetrameranthus*	93/88/1	two support values ≥ BS 85%/PP 0.95
C*: *Mezzettia-Ambavia*	98/69/1	not resolved
D*:	100/100/1	two support values ≥ BS 85%/PP 0.95
E*:	96/99/1	one support values ≥ BS 85%/PP 0.95
F*: *Asimina*	100/100/1	no support
G*: tribe Monodoreae	97/71/0.86	no support
H*:	99/98/0.98	two support values ≥ BS 85%/PP 0.95
I*:	99/92/1	one support values ≥ BS 85%/PP 0.95
J*: *Hexalobus-Uvariastrum*	100/92/0.95	two support values ≥ BS 85%/PP 0.95
K*: *Monocyclanthus-Uvariopsis*	98/90/1	one support values ≥ BS 85%/PP 0.95
L*: *Uvariopsis*	91/-/0.97	one support values ≥ BS 85%/PP 0.95
M*: *Uvariodendron*	98/93/1	no support
N*: *Fissistigma-Mitrella*	100/100/1	one support values ≥ BS 85%/PP 0.95
O*:	99/98/1	one support values ≥ BS 85%/PP 0.95
P*:	97/100/1	two support values ≥ BS 85%/PP 0.95
Q*: *Dasymaschalon*	100/100/1	two support values ≥ BS 85%/PP 0.95
R*: *Desmos*	96/97/1	one support values ≥ BS 85%/PP 0.95
S*: *Monanthotaxis*	96/98/1	one support values ≥ BS 85%/PP 0.95
T:	65/51/-	three support values ≥ BS 85%/PP 0.95
U*: *Onychopetalum-Bocageopsis*	97/85/1	one support values ≥ BS 85%/PP 0.95
V*: *Bocageopsis*	92/87/1	two support values ≥ BS 85%/PP 0.95
W*: *Unonopsis*	100/100/1	two support values ≥ BS 85%/PP 0.95
X: Monocarpieae-Miliuseae	82/51/0.68	three support values ≥ BS 85%/PP 0.95
Y*: tribe Miliuseae	100/100/1	two support values ≥ BS 85%/PP 0.95

Supplementary figure legend

Fig. S1. Full version of best-scoring maximum likelihood tree inferred from a 754-accession dataset of eight chloroplast markers, showing topology of Anaxagoreoideae, Ambavioideae and Bocageeae. ML bootstrap (BS) values ≥ 50, MP jackknife (JK) values ≥ 50 and Bayesian posterior probabilities (PP) values ≥ 0.5 are indicated at each node: BS /JK/ PP. -, represents clade support values < 50%. Node numbers correlate with those shown in supplementary Table S1.

Fig. S2. Full version of best-scoring maximum likelihood tree inferred from a 754-accession dataset of eight chloroplast markers, showing topology of Guatterieae, updated for the Taxonomy[67] in bold. ML bootstrap (BS) values ≥ 50, MP jackknife (JK) values ≥ 50 and Bayesian posterior probabilities (PP) values ≥ 0.5 are indicated at each node: BS /JK/ PP. -, represents clade support values < 50%.

Fig. S3. Full version of best-scoring maximum likelihood tree inferred from a 754-accession dataset of eight chloroplast markers, showing topology of Duguetieae and Xylopieae. ML bootstrap (BS) values ≥ 50, MP jackknife (JK) values ≥ 50 and Bayesian posterior probabilities (PP) values ≥ 0.5 are indicated at each node: BS /JK/ PP. -, represents clade support values < 50%. Species names within *Duguetia* are omitted since phylogenetic relationships within the genus have not been formally published by those contributing the sequences to GenBank.

Fig. S4. Full version of best-scoring maximum likelihood tree inferred from a 754-accession dataset of eight chloroplast markers, showing topology of Annoneae. ML bootstrap (BS) values ≥ 50, MP jackknife (JK) values ≥ 50 and Bayesian posterior probabilities (PP) values ≥ 0.5 are indicated at each node: BS /JK/ PP. -, represents clade support values < 50%. Node numbers correlate with those shown in supplementary Table S1.

Fig. S5. Full version of best-scoring maximum likelihood tree inferred from a 754-accession dataset of eight chloroplast markers, showing topology of Monodoreae. ML bootstrap (BS) values ≥ 50, MP jackknife (JK) values ≥ 50 and Bayesian posterior probabilities (PP) values ≥ 0.5 are indicated at each node: BS /JK/ PP. -, represents clade support values < 50%. Node numbers correlate with those shown in supplementary Table S1.

Fig. S6. Full version of best-scoring maximum likelihood tree inferred from a 754-accession dataset of eight chloroplast markers, showing topology of Uvarieae. ML bootstrap (BS) values ≥ 50, MP jackknife (JK) values ≥ 50 and Bayesian posterior probabilities (PP) values ≥ 0.5 are indicated at each node: BS /JK/ PP. -, represents clade support values < 50%. Node numbers correlate with those shown in supplementary Table S1.

Fig. S7. Full version of best-scoring maximum likelihood tree inferred from a 754-accession dataset of eight chloroplast markers, showing topology of Piptostigmateae, Malmeeae, Maasieae, Fenerivieae, Phoenicantheae, Dendrokingstonieae, Monocarpieae and Miliuseae. ML bootstrap (BS) values ≥ 50, MP jackknife (JK) values ≥ 50 and Bayesian posterior probabilities (PP) values ≥ 0.5 are indicated at

each node: BS /JK/ PP. -, represents clade support values < 50%. Node numbers correlate with those shown in supplementary Table S1.

Fig. S8. Full version of best-scoring maximum likelihood tree inferred from a 754-accession dataset of eight chloroplast markers, showing topology of *Miliuseae* (part 1). ML bootstrap (BS) values ≥ 50, MP jackknife (JK) values ≥ 50 and Bayesian posterior probabilities (PP) values ≥ 0.5 are indicated at each node: BS /JK/ PP. -, represents clade support values < 50%. Node numbers correlate with those shown in supplementary Table S1.

Fig. S9. Full version of best-scoring maximum likelihood tree inferred from a 754-accession dataset of eight chloroplast markers, showing topology of *Miliuseae* (part 2). ML bootstrap (BS) values ≥ 50, MP jackknife (JK) values ≥ 50 and Bayesian posterior probabilities (PP) values ≥ 0.5 are indicated at each node: BS /JK/ PP. -, represents clade support values < 50%.

Fig. S1

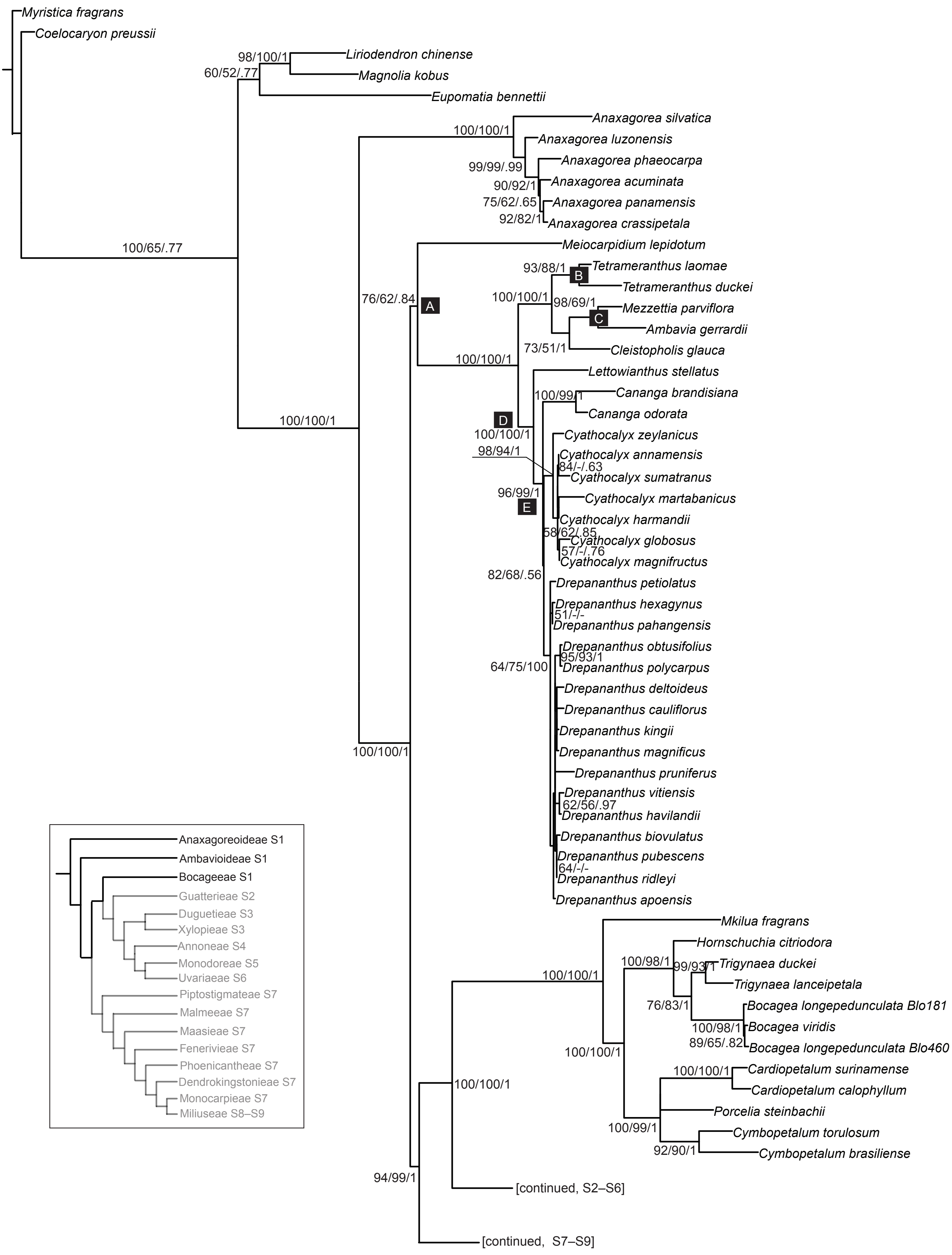
Myristica fragrans
Coelocaryon preussii
98/100/1
Liriodendron chinense
60/52/.77
Magnolia kobus
Eupomatia bennettii
Anaxagorea silvatica
100/100/1
Anaxagorea luzonensis
Anaxagorea phaeocarpa
99/99/.99
90/92/1
Anaxagorea acuminata
75/62/.65
Anaxagorea panamensis
92/82/1
Anaxagorea crassipetala
100/65/.77
Meiocarpidium lepidotum
93/88/1
B
Tetrameranthus laomae
Tetrameranthus duckei
76/62/.84
A
100/100/1
98/69/1
Mezzettia parviflora
C
Ambavia gerrardii
73/51/1
Cleistopholis glauca
100/100/1
Lettowianthus stellatus
100/99/1
Cananga brandisiana
Cananga odorata
D
100/100/1
100/100/1
Cyathocalyx zeylanicus
98/94/1
Cyathocalyx annamensis
84/-/.63
Cyathocalyx sumatranus
96/99/1
E
Cyathocalyx martabanicus
Cyathocalyx harmandii
58/62/.85
Cyathocalyx globosus
57/-/.76
Cyathocalyx magnifructus
82/68/.56
Drepananthus petiolatus
Drepananthus hexagynus
51/-/-
Drepananthus pahangensis
Drepananthus obtusifolius
95/93/1
64/75/100
Drepananthus polycarpus
Drepananthus deltoideus
Drepananthus cauliflorus
Drepananthus kingii
100/100/1
Drepananthus magnificus
Drepananthus pruniferus
Drepananthus vitiensis
62/56/.97
Drepananthus havilandii
Drepananthus biovulatus
Drepananthus pubescens
64/-/-
Drepananthus ridleyi
Drepananthus apoensis
Anaxagoreoideae S1
Ambavioideae S1
Bocageeae S1
Guatterieae S2
Duguetieae S3
Xylopieae S3
Annoneae S4
Monodoreae S5
Uvariaeae S6
Piptostigmateae S7
Malmeeae S7
Maasieae S7
Fenerivieae S7
Phoenicantheae S7
Dendrokingstonieae S7
Monocarpieae S7
Miliuseae S8–S9
Mkilua fragrans
Hornschuchia citriodora
100/98/1
99/93/1
Trigynaea duckei
100/100/1
Trigynaea lanceipetala
76/83/1
Bocagea longepedunculata Blo181
100/98/1
Bocagea viridis
89/65/.82
Bocagea longepedunculata Blo460
100/100/1
100/100/1
Cardiopetalum surinamense
100/100/1
Cardiopetalum calophyllum
Porcelia steinbachii
100/99/1
Cymbopetalum torulosum
92/90/1
Cymbopetalum brasiliense
94/99/1
[continued, S2–S6]
[continued, S7–S9]

Fig. S2

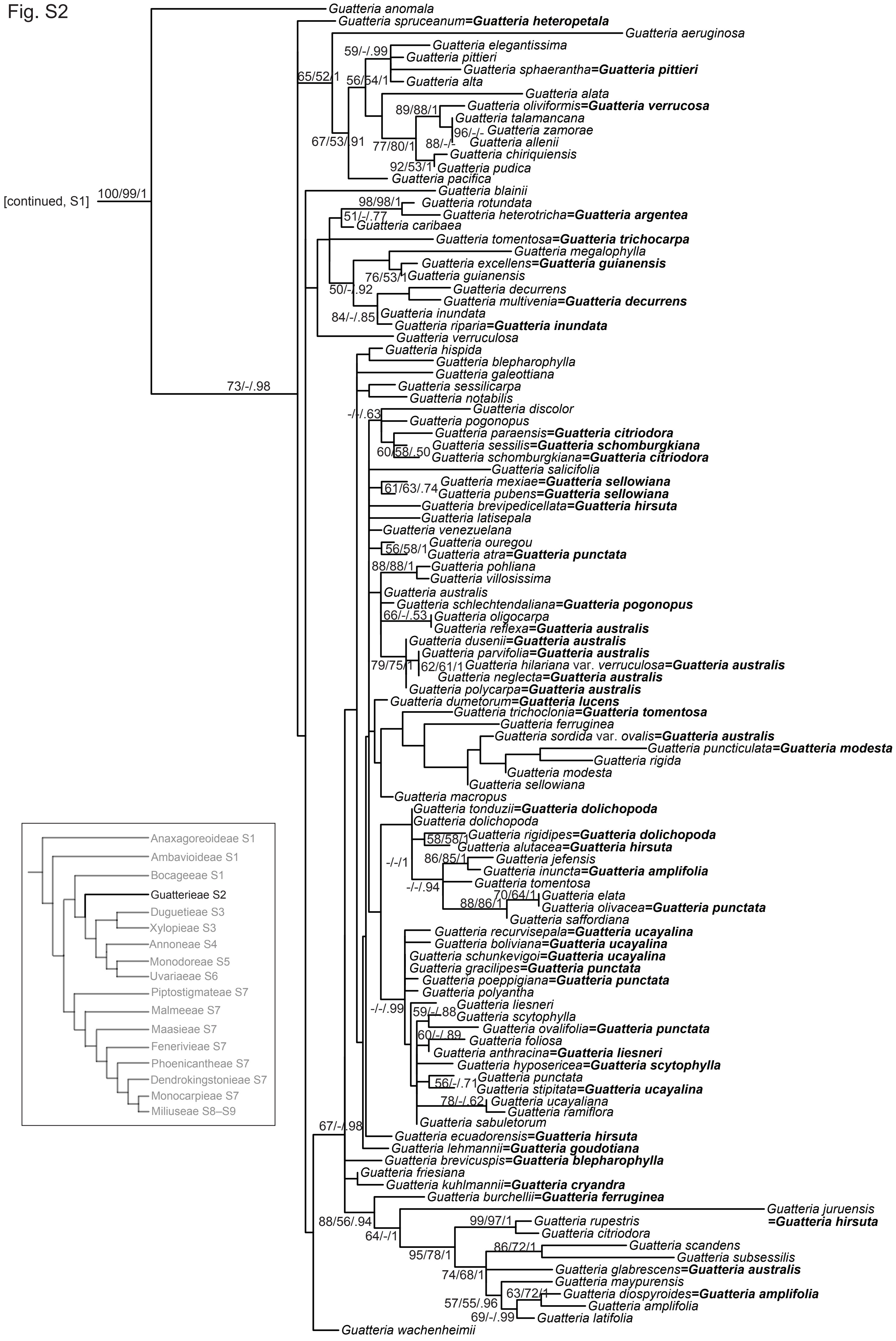
Guatteria anomala
Guatteria spruceanum=Guatteria heteropetala
Guatteria aeruginosa
Guatteria elegantissima
Guatteria pittieri
Guatteria sphaerantha=Guatteria pittieri
Guatteria alta
Guatteria alata
Guatteria oliviformis=Guatteria verrucosa
Guatteria talamancana
Guatteria zamorae
Guatteria allenii
Guatteria chiriquiensis
Guatteria pudica
Guatteria pacifica
[continued, S1]
100/99/1
65/52/1
59/-/.99
56/54/1
89/88/1
96/-/-
88/-/-
67/53/.91
77/80/1
92/53/1
Guatteria blainii
Guatteria rotundata
Guatteria heterotricha=Guatteria argentea
Guatteria caribaea
Guatteria tomentosa=Guatteria trichocarpa
Guatteria megalophylla
Guatteria excellens=Guatteria guianensis
Guatteria guianensis
Guatteria decurrens
Guatteria multivenia=Guatteria decurrens
Guatteria inundata
Guatteria riparia=Guatteria inundata
Guatteria verruculosa
98/98/1
51/-/.77
76/53/1
50/-/.92
84/-/.85
73/-/.98
Guatteria hispida
Guatteria blepharophylla
Guatteria galeottiana
Guatteria sessilicarpa
Guatteria notabilis
Guatteria discolor
Guatteria pogonopus
Guatteria paraensis=Guatteria citriodora
Guatteria sessilis=Guatteria schomburgkiana
Guatteria schomburgkiana=Guatteria citriodora
Guatteria salicifolia
Guatteria mexiae=Guatteria sellowiana
Guatteria pubens=Guatteria sellowiana
Guatteria brevipedicellata=Guatteria hirsuta
Guatteria latisepala
Guatteria venezuelana
Guatteria ouregou
Guatteria atra=Guatteria punctata
Guatteria pohliana
Guatteria villosissima
Guatteria australis
Guatteria schlechtendaliana=Guatteria pogonopus
Guatteria oligocarpa
Guatteria reflexa=Guatteria australis
Guatteria dusenii=Guatteria australis
Guatteria parvifolia=Guatteria australis
Guatteria hilariana var. verruculosa=Guatteria australis
Guatteria neglecta=Guatteria australis
Guatteria polycarpa=Guatteria australis
-/-/.63
60/58/.50
61/63/.74
56/58/1
88/88/1
66/-/.53
79/75/1
62/61/1
Guatteria dumetorum=Guatteria lucens
Guatteria trichoclonia=Guatteria tomentosa
Guatteria ferruginea
Guatteria sordida var. ovalis=Guatteria australis
Guatteria puncticulata=Guatteria modesta
Guatteria rigida
Guatteria modesta
Guatteria sellowiana
Guatteria macropus
Guatteria tonduzii=Guatteria dolichopoda
Guatteria dolichopoda
Guatteria rigidipes=Guatteria dolichopoda
Guatteria alutacea=Guatteria hirsuta
Guatteria jefensis
Guatteria inuncta=Guatteria amplifolia
Guatteria tomentosa
Guatteria elata
Guatteria olivacea=Guatteria punctata
Guatteria saffordiana
-/-/1
58/58/1
86/85/1
-/-/.94
70/64/1
88/86/1
Guatteria recurvisepala=Guatteria ucayalina
Guatteria boliviana=Guatteria ucayalina
Guatteria schunkevigoi=Guatteria ucayalina
Guatteria gracilipes=Guatteria punctata
Guatteria poeppigiana=Guatteria punctata
Guatteria polyantha
Guatteria liesneri
Guatteria scytophylla
Guatteria ovalifolia=Guatteria punctata
Guatteria foliosa
Guatteria anthracina=Guatteria liesneri
Guatteria hyposericea=Guatteria scytophylla
Guatteria punctata
Guatteria stipitata=Guatteria ucayalina
Guatteria ucayaliana
Guatteria ramiflora
Guatteria sabuletorum
-/-/.99
59/-/.88
60/-/.89
56/-/.71
78/-/.62
Guatteria ecuadorensis=Guatteria hirsuta
Guatteria lehmannii=Guatteria goudotiana
Guatteria brevicuspis=Guatteria blepharophylla
Guatteria friesiana
Guatteria kuhlmannii=Guatteria cryandra
Guatteria burchellii=Guatteria ferruginea
Guatteria juruensis
=Guatteria hirsuta
Guatteria rupestris
Guatteria citriodora
Guatteria scandens
Guatteria subsessilis
Guatteria glabrescens=Guatteria australis
Guatteria maypurensis
Guatteria diospyroides=Guatteria amplifolia
Guatteria amplifolia
Guatteria latifolia
Guatteria wachenheimii
67/-/.98
88/56/.94
64/-/1
99/97/1
95/78/1
86/72/1
74/68/1
63/72/1
57/55/.96
69/-/.99
Anaxagoreoideae S1
Ambavioideae S1
Bocageeae S1
Guatterieae S2
Duguetieae S3
Xylopieae S3
Annoneae S4
Monodoreae S5
Uvariaeae S6
Piptostigmateae S7
Malmeeae S7
Maasieae S7
Fenerivieae S7
Phoenicantheae S7
Dendrokingstonieae S7
Monocarpieae S7
Miliuseae S8–S9

Fig. S3

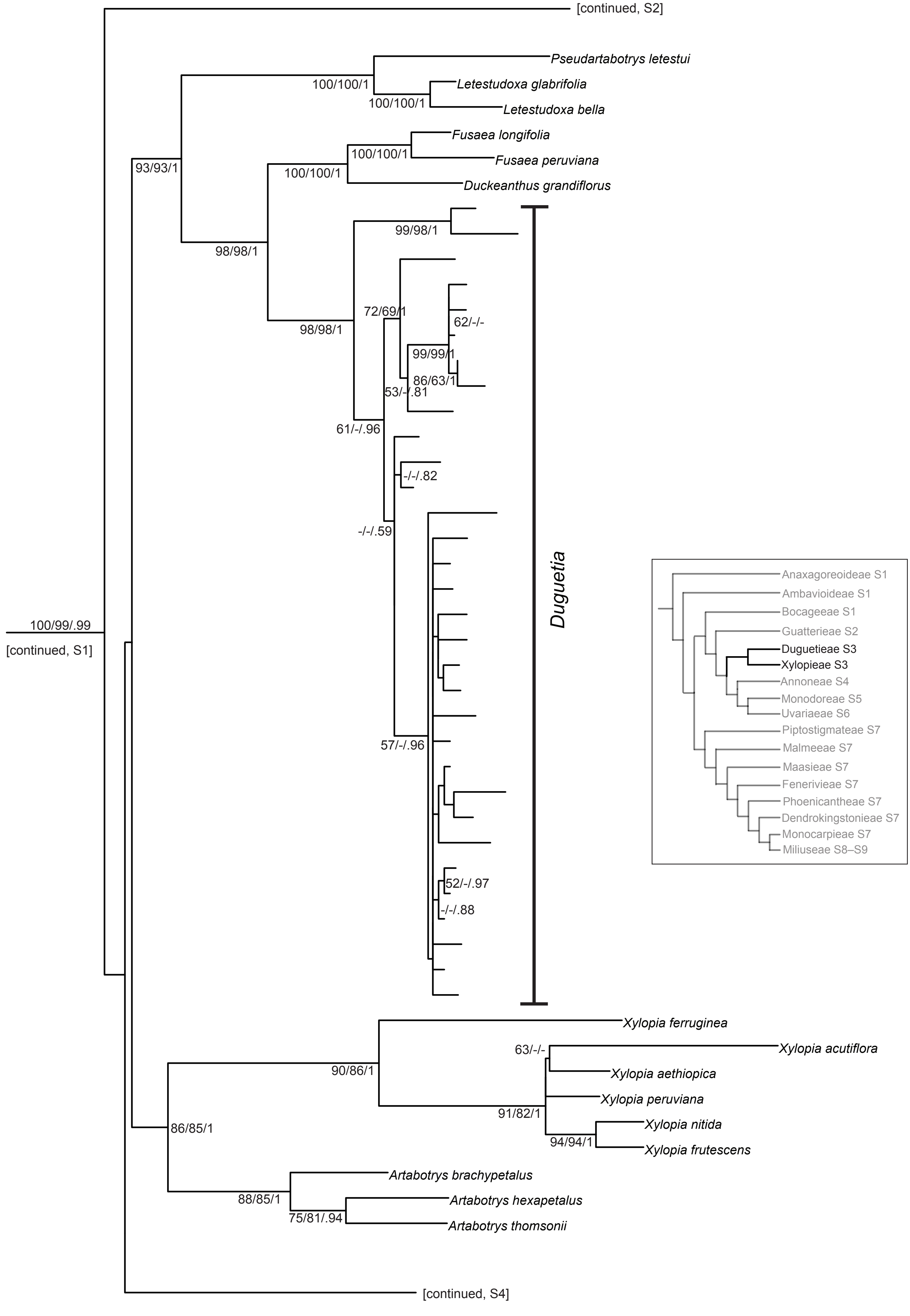

[continued, S2]
Pseudartabotrys letestui
100/100/1
Letestudoxa glabrifolia
100/100/1
Letestudoxa bella
Fusaea longifolia
100/100/1
Fusaea peruviana
93/93/1
100/100/1
Duckeanthus grandiflorus
99/98/1
98/98/1
72/69/1
62/-/-
98/98/1
99/99/1
86/63/1
53/-/.81
61/-/.96
-/-/.82
-/-/.59
Duguetia
100/99/.99
[continued, S1]
57/-/.96
52/-/.97
-/-/.88
Xylopia ferruginea
63/-/-
Xylopia acutiflora
90/86/1
Xylopia aethiopica
Xylopia peruviana
91/82/1
86/85/1
Xylopia nitida
94/94/1
Xylopia frutescens
Artabotrys brachypetalus
88/85/1
Artabotrys hexapetalus
75/81/.94
Artabotrys thomsonii
[continued, S4]
Anaxagoreoideae S1
Ambavioideae S1
Bocageeae S1
Guatterieeae S2
Duguetieae S3
Xylopieae S3
Annoneae S4
Monodoreae S5
Uvariaeae S6
Piptostigmateae S7
Malmeeae S7
Maasieae S7
Fenerivieae S7
Phoenicantheae S7
Dendrokingstonieae S7
Monocarpieae S7
Miliuseae S8–S9

Fig. S4

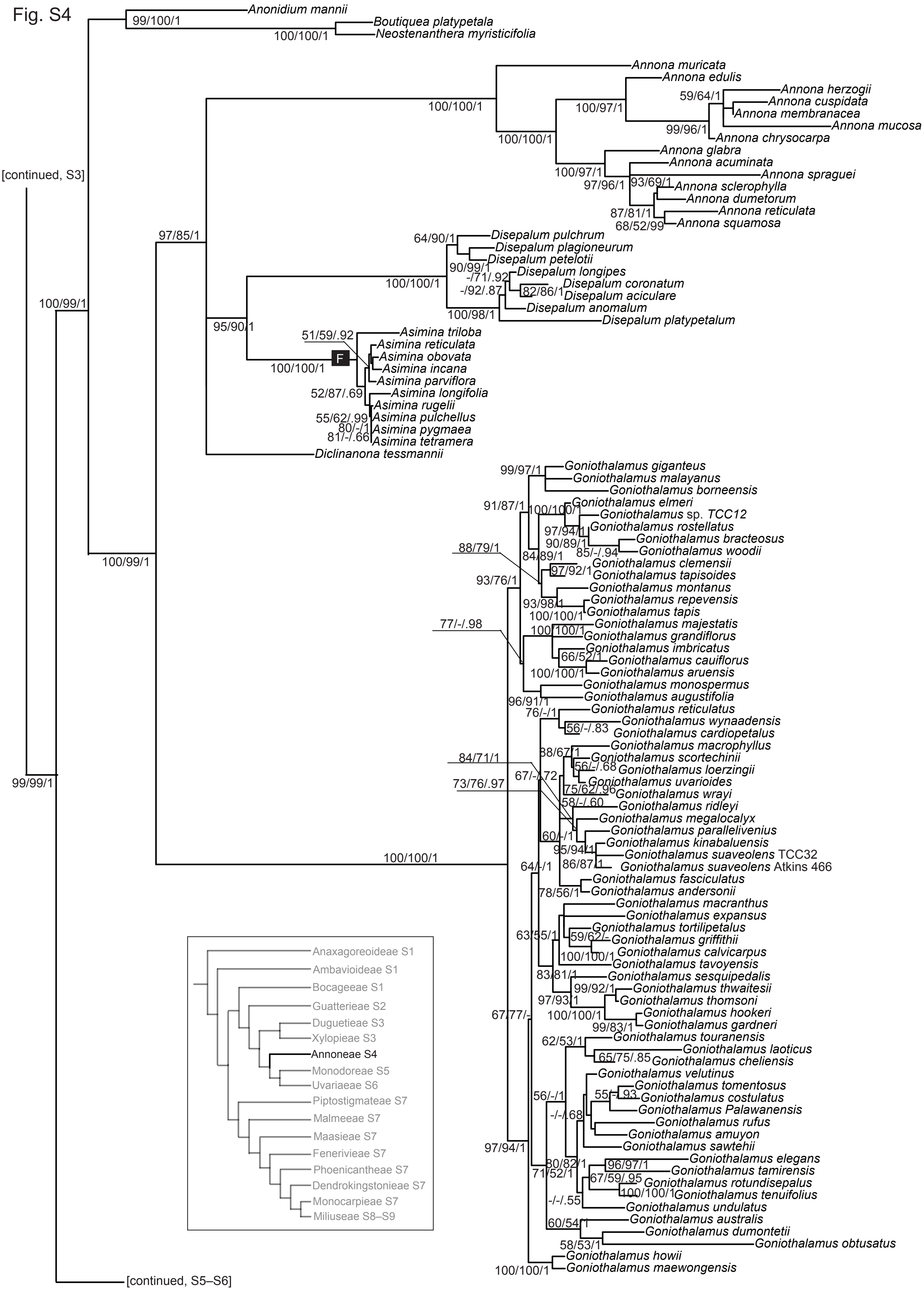

Anonidium mannii
99/100/1
Boutiquea platypetala
100/100/1
Neostenanthera myristicifolia
Annona muricata
Annona edulis
59/64/1
Annona herzogii
Annona cuspidata
100/97/1
Annona membranacea
100/100/1
Annona mucosa
99/96/1
Annona chrysocarpa
100/100/1
Annona glabra
Annona acuminata
[continued, S3]
100/97/1
Annona spraguei
97/96/1
93/69/1
Annona sclerophylla
Annona dumetorum
87/81/1
Annona reticulata
68/52/99
Annona squamosa
97/85/1
Disepalum pulchrum
64/90/1
Disepalum plagioneurum
90/99/1
Disepalum petelotii
-/71/.92
Disepalum longipes
100/100/1
Disepalum coronatum
-/92/.87
82/86/1
Disepalum aciculare
100/100/1
Disepalum anomalum
100/98/1
Disepalum platypetalum
100/99/1
95/90/1
Asimina triloba
51/59/.92
Asimina reticulata
F
Asimina obovata
100/100/1
Asimina incana
Asimina parviflora
52/87/.69
Asimina longifolia
Asimina rugelii
55/62/.99
Asimina pulchellus
80/-/1
Asimina pygmaea
81/-/.66
Asimina tetramera
Diclinanona tessmannii
99/97/1
Goniothalamus giganteus
Goniothalamus malayanus
Goniothalamus borneensis
91/87/1
Goniothalamus elmeri
100/100/1
Goniothalamus sp. TCC12
97/94/1
Goniothalamus rostellatus
90/89/1
Goniothalamus bracteosus
88/79/1
85/-/.94
Goniothalamus woodii
84/89/1
Goniothalamus clemensii
100/99/1
97/92/1
Goniothalamus tapisoides
93/76/1
Goniothalamus montanus
93/98/1
Goniothalamus repevensis
100/100/1
Goniothalamus tapis
77/-/.98
Goniothalamus majestatis
100/100/1
Goniothalamus grandiflorus
Goniothalamus imbricatus
66/52/1
Goniothalamus cauiflorus
100/100/1
Goniothalamus aruensis
Goniothalamus monospermus
96/91/1
Goniothalamus augustifolia
76/-/1
Goniothalamus reticulatus
Goniothalamus wynaadensis
56/-/.83
Goniothalamus cardiopetalus
Goniothalamus macrophyllus
88/67/1
84/71/1
Goniothalamus scortechinii
56/-/.68
Goniothalamus loerzingii
67/-/.72
Goniothalamus uvarioides
73/76/.97
75/62/.96
Goniothalamus wrayi
99/99/1
58/-/.60
Goniothalamus ridleyi
Goniothalamus megalocalyx
60/-/1
Goniothalamus parallelivenius
Goniothalamus kinabaluensis
95/94/1
Goniothalamus suaveolens TCC32
100/100/1
86/87/1
Goniothalamus suaveolens Atkins 466
64/-/1
Goniothalamus fasciculatus
78/56/1
Goniothalamus andersonii
Goniothalamus macranthus
Goniothalamus expansus
Goniothalamus tortilipetalus
63/55/1
59/62/-
Goniothalamus griffithii
Goniothalamus calvicarpus
100/100/1
Goniothalamus tavoyensis
83/81/1
Goniothalamus sesquipedalis
99/92/1
Goniothalamus thwaitesii
97/93/1
Goniothalamus thomsoni
100/100/1
Goniothalamus hookeri
67/77/-
99/83/1
Goniothalamus gardneri
62/53/1
Goniothalamus touranensis
Goniothalamus laoticus
65/75/.85
Goniothalamus cheliensis
Goniothalamus velutinus
Goniothalamus tomentosus
56/-/1
55/-/.93
Goniothalamus costulatus
Goniothalamus Palawanensis
-/-/.68
Goniothalamus rufus
Goniothalamus amuyon
97/94/1
Goniothalamus sawtehii
96/97/1
Goniothalamus elegans
80/82/1
Goniothalamus tamirensis
71/52/1
67/59/.95
Goniothalamus rotundisepalus
100/100/1
Goniothalamus tenuifolius
-/-/.55
Goniothalamus undulatus
60/54/1
Goniothalamus australis
Goniothalamus dumontetii
58/53/1
Goniothalamus obtusatus
Goniothalamus howii
100/100/1
Goniothalamus maewongensis
[continued, S5–S6]
Anaxagoreoideae S1
Ambavioideae S1
Bocageeae S1
Guatterieae S2
Duguetieae S3
Xylopieae S3
Annoneae S4
Monodoreae S5
Uvariaeae S6
Piptostigmateae S7
Malmeeae S7
Maasieae S7
Fenerivieae S7
Phoenicantheae S7
Dendrokingstonieae S7
Monocarpieae S7
Miliuseae S8–S9

Fig. S5

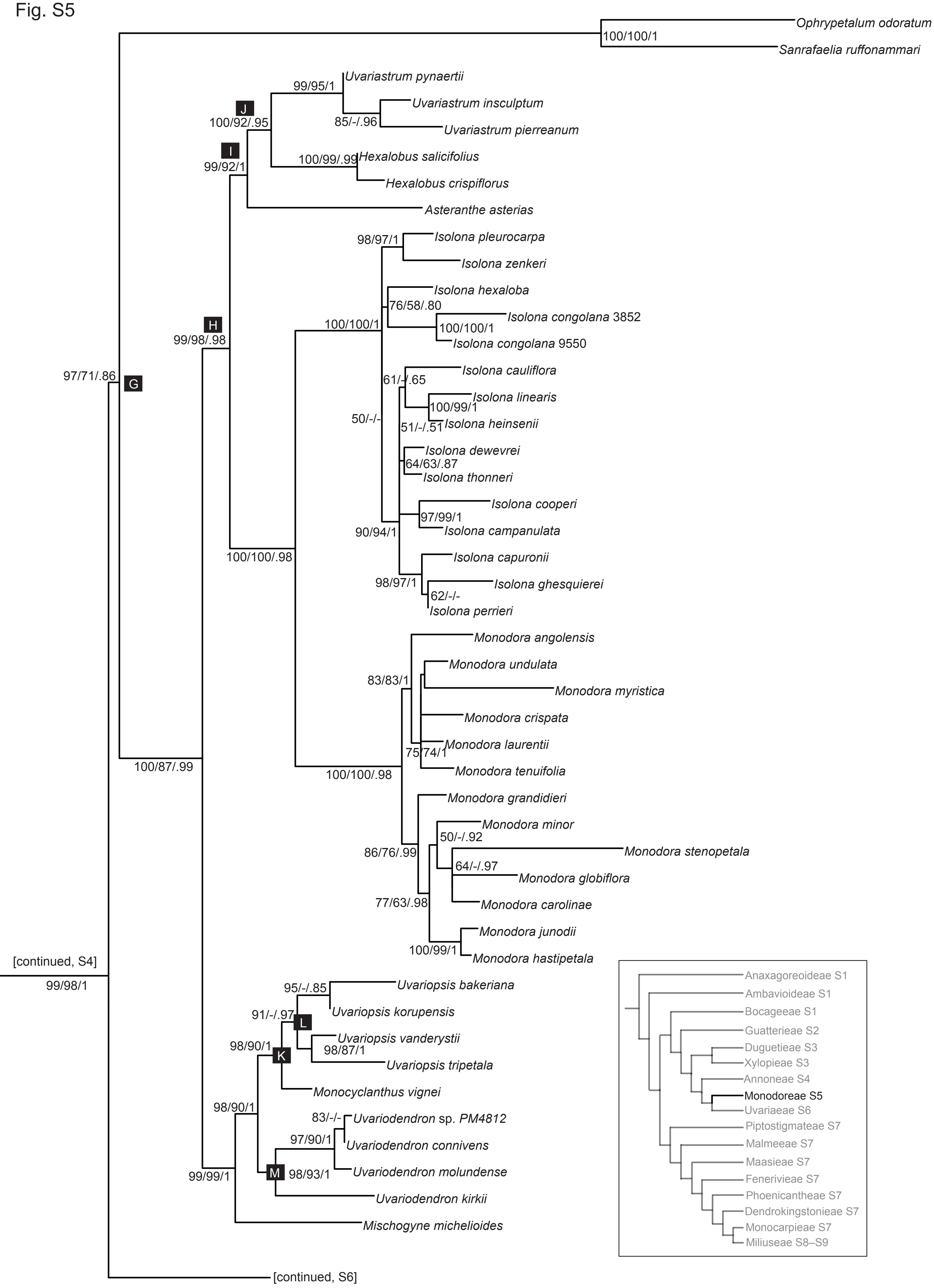
Ophrypetalum odoratum
Sanrafaelia ruffonammari
100/100/1
Uvariastrum pynaertii
99/95/1
Uvariastrum insculptum
Uvariastrum pierreanum
85/-/.96
J
100/92/.95
I
99/92/1
Hexalobus salicifolius
100/99/.99
Hexalobus crispiflorus
Asteranthe asterias
Isolona pleurocarpa
98/97/1
Isolona zenkeri
Isolona hexaloba
76/58/.80
Isolona congolana 3852
100/100/1
Isolona congolana 9550
100/100/1
H
99/98/.98
97/71/.86
G
Isolona cauliflora
61/-/.65
Isolona linearis
100/99/1
50/-/-
51/-/.51
Isolona heinsenii
Isolona dewevrei
64/63/.87
Isolona thonneri
Isolona cooperi
97/99/1
Isolona campanulata
90/94/1
100/100/.98
Isolona capuronii
98/97/1
Isolona ghesquierei
62/-/-
Isolona perrieri
Monodora angolensis
Monodora undulata
83/83/1
Monodora myristica
Monodora crispata
Monodora laurentii
75/74/1
Monodora tenuifolia
100/87/.99
100/100/.98
Monodora grandidieri
Monodora minor
50/-/.92
Monodora stenopetala
86/76/.99
64/-/.97
Monodora globiflora
77/63/.98
Monodora carolinae
Monodora junodii
100/99/1
Monodora hastipetala
[continued, S4]
99/98/1
95/-/.85
Uvariopsis bakeriana
91/-/.97
Uvariopsis korupensis
L
Uvariopsis vanderystii
98/90/1
98/87/1
K
Uvariopsis tripetala
Monocyclanthus vignei
98/90/1
83/-/-
Uvariodendron sp. PM4812
97/90/1
Uvariodendron connivens
M
98/93/1
Uvariodendron molundense
99/99/1
Uvariodendron kirkii
Mischogyne michelioides
[continued, S6]
Anaxagoreoideae S1
Ambavioideae S1
Bocageeae S1
Guatterieae S2
Duguetieae S3
Xylopieae S3
Annoneae S4
Monodoreae S5
Uvariaeae S6
Piptostigmateae S7
Malmeeae S7
Maasieae S7
Fenerivieae S7
Phoenicantheae S7
Dendrokingstonieae S7
Monocarpieae S7
Miliuseae S8–S9

Fig. S6

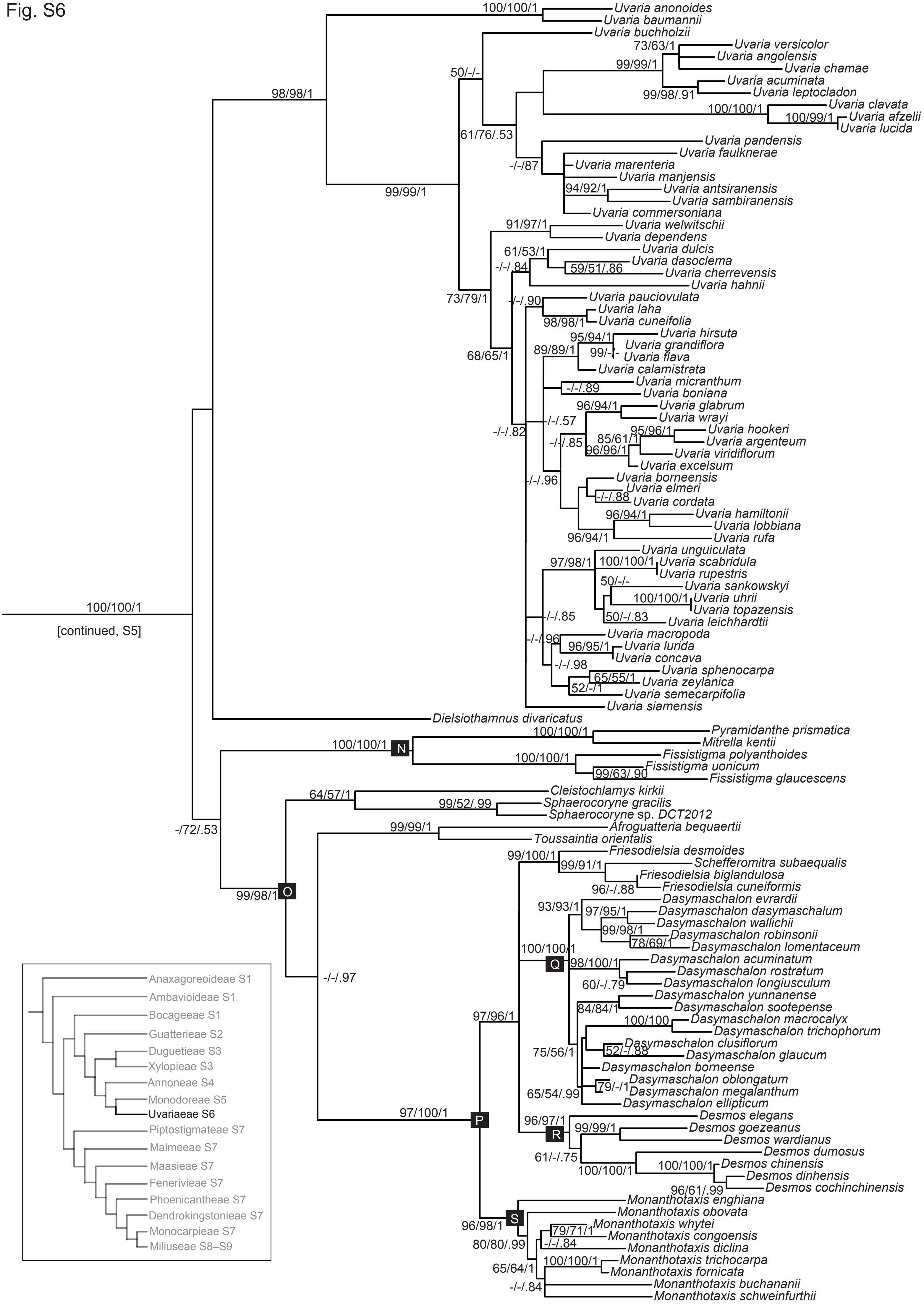
100/100/1
Uvaria anonoides
Uvaria baumannii
98/98/1
Uvaria buchholzii
73/63/1
Uvaria versicolor
Uvaria angolensis
99/99/1
Uvaria chamae
Uvaria acuminata
50/-/-
99/98/.91
Uvaria leptocladon
Uvaria clavata
100/100/1
100/99/1
Uvaria afzelii
Uvaria lucida
61/76/.53
Uvaria pandensis
Uvaria faulknerae
-/-/87
Uvaria marenteria
Uvaria manjensis
94/92/1
Uvaria antsiranensis
99/99/1
Uvaria sambiranensis
Uvaria commersoniana
91/97/1
Uvaria welwitschii
Uvaria dependens
61/53/1
Uvaria dulcis
-/-/.84
Uvaria dasoclema
59/51/.86
Uvaria cherrevensis
Uvaria hahnii
73/79/1
-/-/.90
Uvaria pauciovulata
Uvaria laha
98/98/1
Uvaria cuneifolia
95/94/1
Uvaria hirsuta
Uvaria grandiflora
89/89/1
99/-/-
Uvaria flava
68/65/1
Uvaria calamistrata
Uvaria micranthum
-/-/.89
Uvaria boniana
96/94/1
Uvaria glabrum
-/-/.57
Uvaria wrayi
95/96/1
Uvaria hookeri
-/-/.82
85/61/1
-/-/.85
Uvaria argenteum
96/96/1
Uvaria viridiflorum
Uvaria excelsum
Uvaria borneensis
-/-/.96
Uvaria elmeri
-/-/.88
Uvaria cordata
96/94/1
Uvaria hamiltonii
Uvaria lobbiana
96/94/1
Uvaria rufa
Uvaria unguiculata
97/98/1
100/100/1
Uvaria scabridula
Uvaria rupestris
50/-/-
Uvaria sankowskyi
100/100/1
Uvaria uhrii
100/100/1
Uvaria topazensis
-/-/.85
50/-/.83
Uvaria leichhardtii
[continued, S5]
Uvaria macropoda
-/-/.96
96/95/1
Uvaria lurida
Uvaria concava
-/-/.98
Uvaria sphenocarpa
65/55/1
Uvaria zeylanica
52/-/1
Uvaria semecarpifolia
Uvaria siamensis
Dielsiothamnus divaricatus
100/100/1
Pyramidanthe prismatica
100/100/1
N
Mitrella kentii
100/100/1
Fissistigma polyanthoides
Fissistigma uonicum
99/63/.90
Fissistigma glaucescens
64/57/1
Cleistochlamys kirkii
99/52/.99
Sphaerocoryne gracilis
-/72/.53
Sphaerocoryne sp. DCT2012
99/99/1
Afroguatteria bequaertii
Toussaintia orientalis
99/100/1
Friesodielsia desmoides
99/91/1
Schefferomitra subaequalis
Friesodielsia biglandulosa
99/98/1
O
96/-/.88
Friesodielsia cuneiformis
Dasymaschalon evrardii
93/93/1
97/95/1
Dasymaschalon dasymaschalum
Dasymaschalon wallichii
99/98/1
Dasymaschalon robinsonii
78/69/1
Dasymaschalon lomentaceum
100/100/1
Q
98/100/1
Dasymaschalon acuminatum
Dasymaschalon rostratum
60/-/.79
Dasymaschalon longiusculum
Dasymaschalon yunnanense
84/84/1
Dasymaschalon sootepense
97/96/1
100/100
Dasymaschalon macrocalyx
Dasymaschalon trichophorum
Dasymaschalon clusiflorum
75/56/1
52/-/.88
Dasymaschalon glaucum
Dasymaschalon borneense
79/-/1
Dasymaschalon oblongatum
Dasymaschalon megalanthum
65/54/.99
Dasymaschalon ellipticum
97/100/1
P
Desmos elegans
96/97/1
R
99/99/1
Desmos goezeanus
Desmos wardianus
Desmos dumosus
61/-/.75
100/100/1
Desmos chinensis
100/100/1
Desmos dinhensis
96/61/.99
Desmos cochinchinensis
Monanthotaxis enghiana
Monanthotaxis obovata
96/98/1
S
79/71/1
Monanthotaxis whytei
Monanthotaxis congoensis
80/80/.99
-/-/.84
Monanthotaxis diclina
100/100/1
Monanthotaxis trichocarpa
Monanthotaxis fornicata
65/64/1
Monanthotaxis buchananii
-/-/.84
Monanthotaxis schweinfurthii
Anaxagoreoideae S1
Ambavioideae S1
Bocageeae S1
Guatterieae S2
Duguetieae S3
Xylopieae S3
Annoneae S4
Monodoreae S5
Uvariaeae S6
Piptostigmateae S7
Malmeeae S7
Maasieae S7
Fenerivieae S7
Phoenicantheae S7
Dendrokingstonieae S7
Monocarpieae S7
Miliuseae S8–S9

Fig. S7

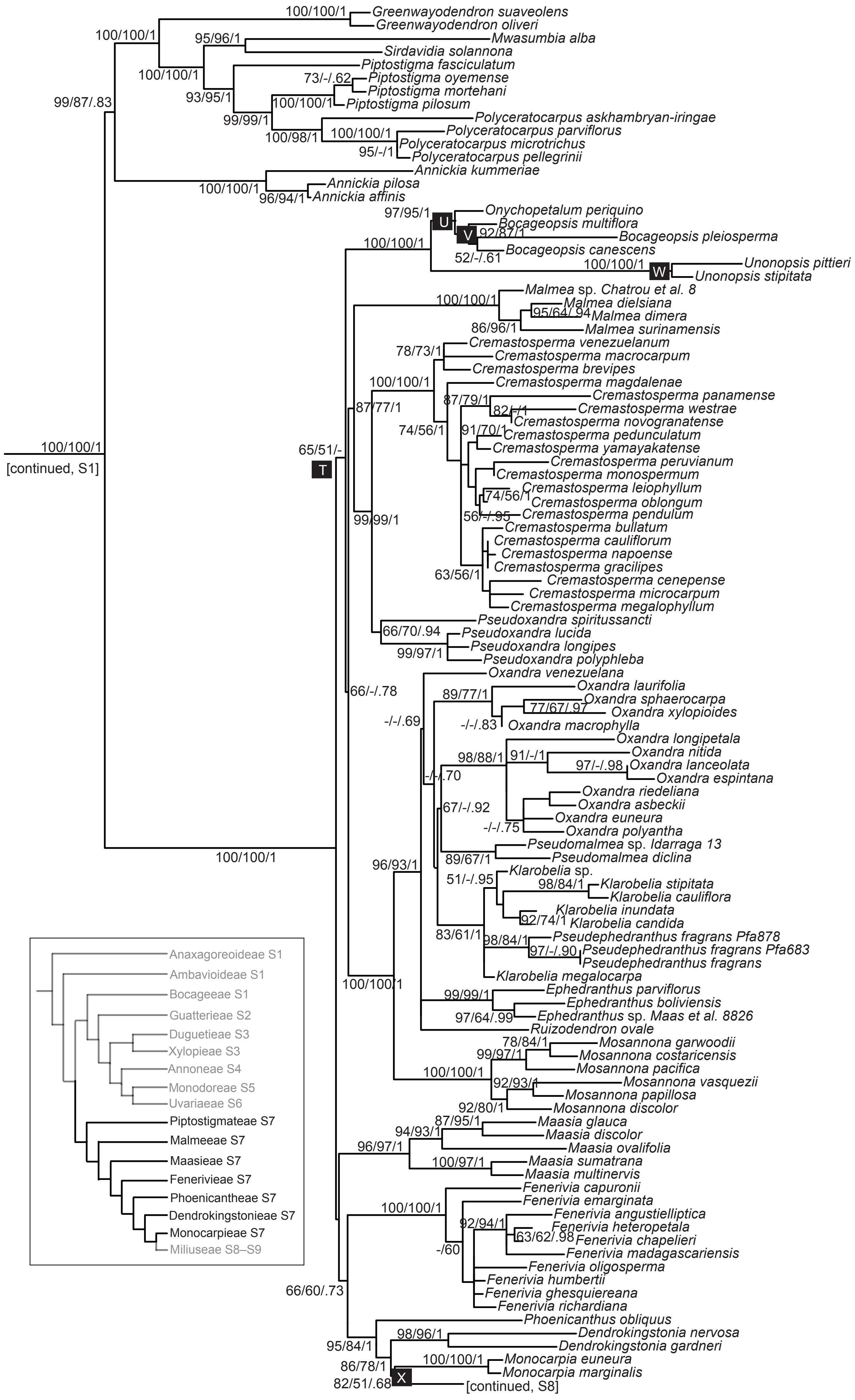

100/100/1
Greenwayodendron suaveolens
Greenwayodendron oliveri
100/100/1
95/96/1
Mwasumbia alba
Sirdavidia solannona
100/100/1
Piptostigma fasciculatum
73/-/.62
Piptostigma oyemense
Piptostigma mortehani
99/87/.83
93/95/1
100/100/1
Piptostigma pilosum
99/99/1
Polyceratocarpus askhambryan-iringae
100/100/1
Polyceratocarpus parviflorus
100/98/1
Polyceratocarpus microtrichus
95/-/1
Polyceratocarpus pellegrinii
Annickia kummeriae
100/100/1
Annickia pilosa
96/94/1
Annickia affinis
97/95/1
U
Onychopetalum periquino
Bocageopsis multiflora
V
92/87/1
Bocageopsis pleiosperma
100/100/1
Bocageopsis canescens
52/-/.61
100/100/1
W
Unonopsis pittieri
Unonopsis stipitata
Malmea sp. Chatrou et al. 8
100/100/1
Malmea dielsiana
95/64/.94
Malmea dimera
86/96/1
Malmea surinamensis
Cremastosperma venezuelanum
78/73/1
Cremastosperma macrocarpum
Cremastosperma brevipes
100/100/1
Cremastosperma magdalenae
Cremastosperma panamense
87/79/1
Cremastosperma westrae
87/77/1
82/-/1
Cremastosperma novogranatense
74/56/1
91/70/1
Cremastosperma pedunculatum
100/100/1
Cremastosperma yamayakatense
65/51/-
[continued, S1]
T
Cremastosperma peruvianum
Cremastosperma monospermum
Cremastosperma leiophyllum
74/56/1
Cremastosperma oblongum
99/99/1
56/-/.95
Cremastosperma pendulum
Cremastosperma bullatum
Cremastosperma cauliflorum
Cremastosperma napoense
Cremastosperma gracilipes
63/56/1
Cremastosperma cenepense
Cremastosperma microcarpum
Cremastosperma megalophyllum
Pseudoxandra spiritussancti
66/70/.94
Pseudoxandra lucida
Pseudoxandra longipes
99/97/1
Pseudoxandra polyphleba
Oxandra venezuelana
66/-/.78
89/77/1
Oxandra laurifolia
Oxandra sphaerocarpa
77/67/.97
Oxandra xylopioides
-/-/.69
-/-/.83
Oxandra macrophylla
Oxandra longipetala
98/88/1
91/-/1
Oxandra nitida
97/-/.98
Oxandra lanceolata
-/-/.70
Oxandra espintana
Oxandra riedeliana
67/-/.92
Oxandra asbeckii
Oxandra euneura
-/-/.75
Oxandra polyantha
Pseudomalmea sp. Idarraga 13
100/100/1
89/67/1
Pseudomalmea diclina
96/93/1
Klarobelia sp.
51/-/.95
98/84/1
Klarobelia stipitata
Klarobelia cauliflora
Klarobelia inundata
92/74/1
Klarobelia candida
83/61/1
98/84/1
Pseudephedranthus fragrans Pfa878
97/-/.90
Pseudephedranthus fragrans Pfa683
Pseudephedranthus fragrans
Klarobelia megalocarpa
100/100/1
99/99/1
Ephedranthus parviflorus
Ephedranthus boliviensis
97/64/.99
Ephedranthus sp. Maas et al. 8826
Ruizodendron ovale
78/84/1
Mosannona garwoodii
99/97/1
Mosannona costaricensis
Mosannona pacifica
100/100/1
92/93/1
Mosannona vasquezii
Mosannona papillosa
92/80/1
Mosannona discolor
87/95/1
Maasia glauca
94/93/1
Maasia discolor
96/97/1
Maasia ovalifolia
100/97/1
Maasia sumatrana
Maasia multinervis
Fenerivia capuronii
100/100/1
Fenerivia emarginata
Fenerivia angustielliptica
92/94/1
Fenerivia heteropetala
63/62/.98
Fenerivia chapelieri
-/60
Fenerivia madagascariensis
Fenerivia oligosperma
Fenerivia humbertii
66/60/.73
Fenerivia ghesquiereana
Fenerivia richardiana
Phoenicanthus obliquus
98/96/1
Dendrokingstonia nervosa
95/84/1
Dendrokingstonia gardneri
100/100/1
Monocarpia euneura
86/78/1
Monocarpia marginalis
82/51/.68
X
[continued, S8]
Anaxagoreoideae S1
Ambavioideae S1
Bocageeae S1
Guatterieae S2
Duguetieae S3
Xylopieae S3
Annoneae S4
Monodoreae S5
Uvariaeae S6
Piptostigmateae S7
Malmeeae S7
Maasieae S7
Fenerivieae S7
Phoenicantheae S7
Dendrokingstonieae S7
Monocarpieae S7
Miliuseae S8–S9

Fig. S8

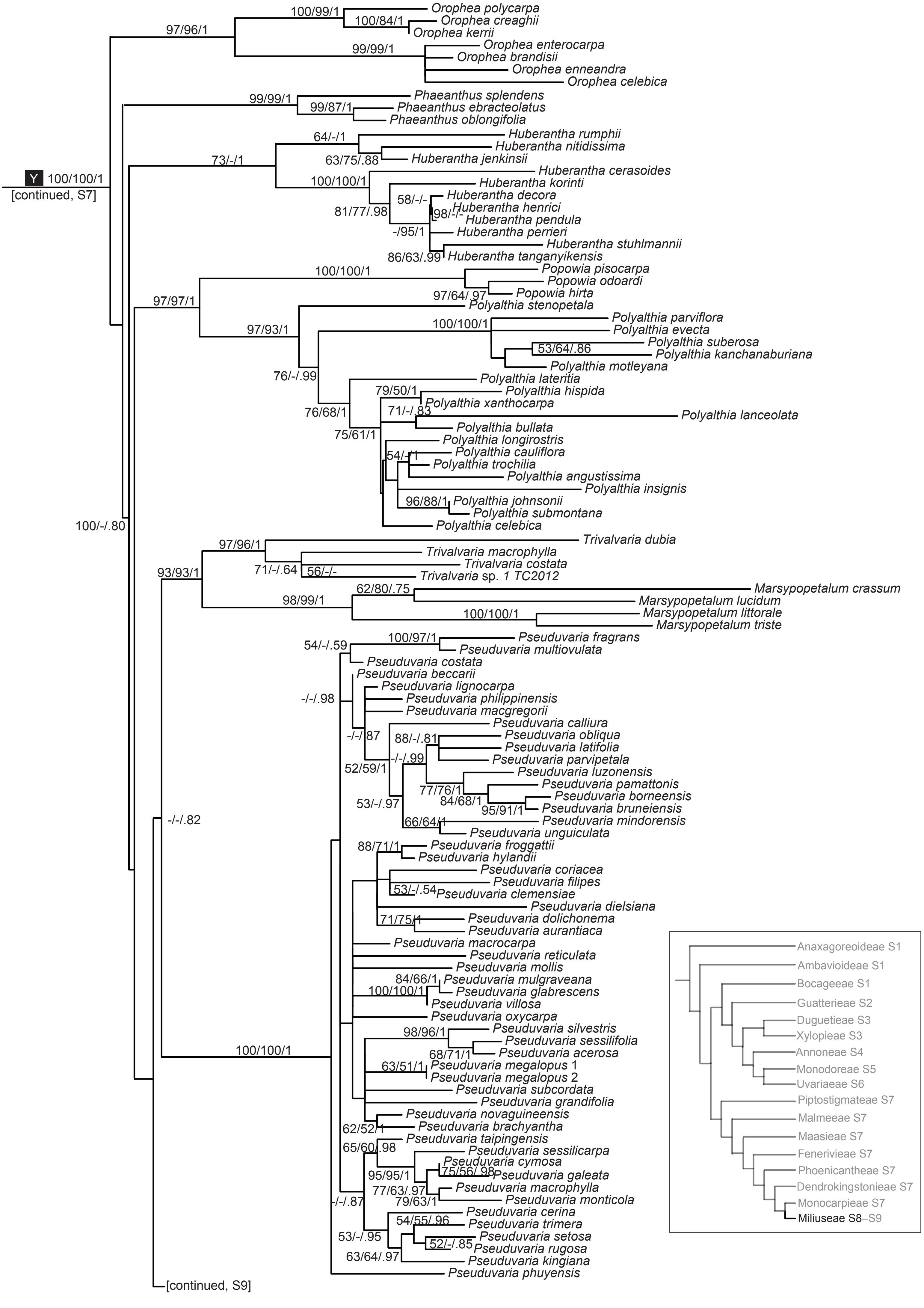

100/99/1
Orophea polycarpa
100/84/1
Orophea creaghii
97/96/1
Orophea kerrii
Orophea enterocarpa
99/99/1
Orophea brandisii
Orophea enneandra
Orophea celebica
99/99/1
Phaeanthus splendens
99/87/1
Phaeanthus ebracteolatus
Phaeanthus oblongifolia
64/-/1
Huberantha rumphii
Huberantha nitidissima
73/-/1
63/75/.88
Huberantha jenkinsii
Y
100/100/1
Huberantha cerasoides
[continued, S7]
100/100/1
Huberantha korinti
58/-/-
Huberantha decora
81/77/.98
98/-/-
Huberantha henrici
Huberantha pendula
-/95/1
Huberantha perrieri
Huberantha stuhlmannii
86/63/.99
Huberantha tanganyikensis
100/100/1
Popowia pisocarpa
Popowia odoardi
97/97/1
97/64/.97
Popowia hirta
Polyalthia stenopetala
100/100/1
Polyalthia parviflora
97/93/1
Polyalthia evecta
53/64/.86
Polyalthia suberosa
Polyalthia kanchanaburiana
76/-/.99
Polyalthia motleyana
Polyalthia lateritia
79/50/1
Polyalthia hispida
Polyalthia xanthocarpa
76/68/1
71/-/.83
Polyalthia lanceolata
Polyalthia bullata
75/61/1
Polyalthia longirostris
54/-/1
Polyalthia cauliflora
Polyalthia trochilia
Polyalthia angustissima
Polyalthia insignis
96/88/1
Polyalthia johnsonii
Polyalthia submontana
100/-/.80
Polyalthia celebica
97/96/1
Trivalvaria dubia
Trivalvaria macrophylla
93/93/1
71/-/.64
Trivalvaria costata
56/-/-
Trivalvaria sp. 1 TC2012
62/80/.75
Marsypopetalum crassum
98/99/1
Marsypopetalum lucidum
100/100/1
Marsypopetalum littorale
Marsypopetalum triste
100/97/1
Pseuduvaria fragrans
54/-/.59
Pseuduvaria multiovulata
Pseuduvaria costata
Pseuduvaria beccarii
Pseuduvaria lignocarpa
-/-/.98
Pseuduvaria philippinensis
Pseuduvaria macgregorii
Pseuduvaria calliura
-/-/.87
88/-/.81
Pseuduvaria obliqua
Pseuduvaria latifolia
-/-/.99
Pseuduvaria parvipetala
52/59/1
Pseuduvaria luzonensis
77/76/1
Pseuduvaria pamattonis
84/68/1
Pseuduvaria borneensis
53/-/.97
95/91/1
Pseuduvaria bruneiensis
-/-/.82
66/64/1
Pseuduvaria mindorensis
Pseuduvaria unguiculata
88/71/1
Pseuduvaria froggattii
Pseuduvaria hylandii
Pseuduvaria coriacea
Pseuduvaria filipes
53/-/.54
Pseuduvaria clemensiae
Pseuduvaria dielsiana
71/75/1
Pseuduvaria dolichonema
Pseuduvaria aurantiaca
Pseuduvaria macrocarpa
Pseuduvaria reticulata
Pseuduvaria mollis
84/66/1
Pseuduvaria mulgraveana
100/100/1
Pseuduvaria glabrescens
Pseuduvaria villosa
Pseuduvaria oxycarpa
98/96/1
Pseuduvaria silvestris
Pseuduvaria sessilifolia
68/71/1
Pseuduvaria acerosa
100/100/1
63/51/1
Pseuduvaria megalopus 1
Pseuduvaria megalopus 2
Pseuduvaria subcordata
Pseuduvaria grandifolia
Pseuduvaria novaguineensis
62/52/1
Pseuduvaria brachyantha
65/60/.98
Pseuduvaria taipingensis
Pseuduvaria sessilicarpa
Pseuduvaria cymosa
95/95/1
75/56/.98
Pseuduvaria galeata
77/63/.97
Pseuduvaria macrophylla
79/63/1
Pseuduvaria monticola
-/-/.87
Pseuduvaria cerina
54/55/.96
Pseuduvaria trimera
53/-/.95
Pseuduvaria setosa
52/-/.85
Pseuduvaria rugosa
63/64/.97
Pseuduvaria kingiana
Pseuduvaria phuyensis
[continued, S9]
Anaxagoreoideae S1
Ambavioideae S1
Bocageeae S1
Guatterieae S2
Duguetieae S3
Xylopieae S3
Annoneae S4
Monodoreae S5
Uvariaeae S6
Piptostigmateae S7
Malmeeae S7
Maasieae S7
Fenerivieae S7
Phoenicantheae S7
Dendrokingstonieae S7
Monocarpieae S7
Miliuseae S8–S9

Fig. S9

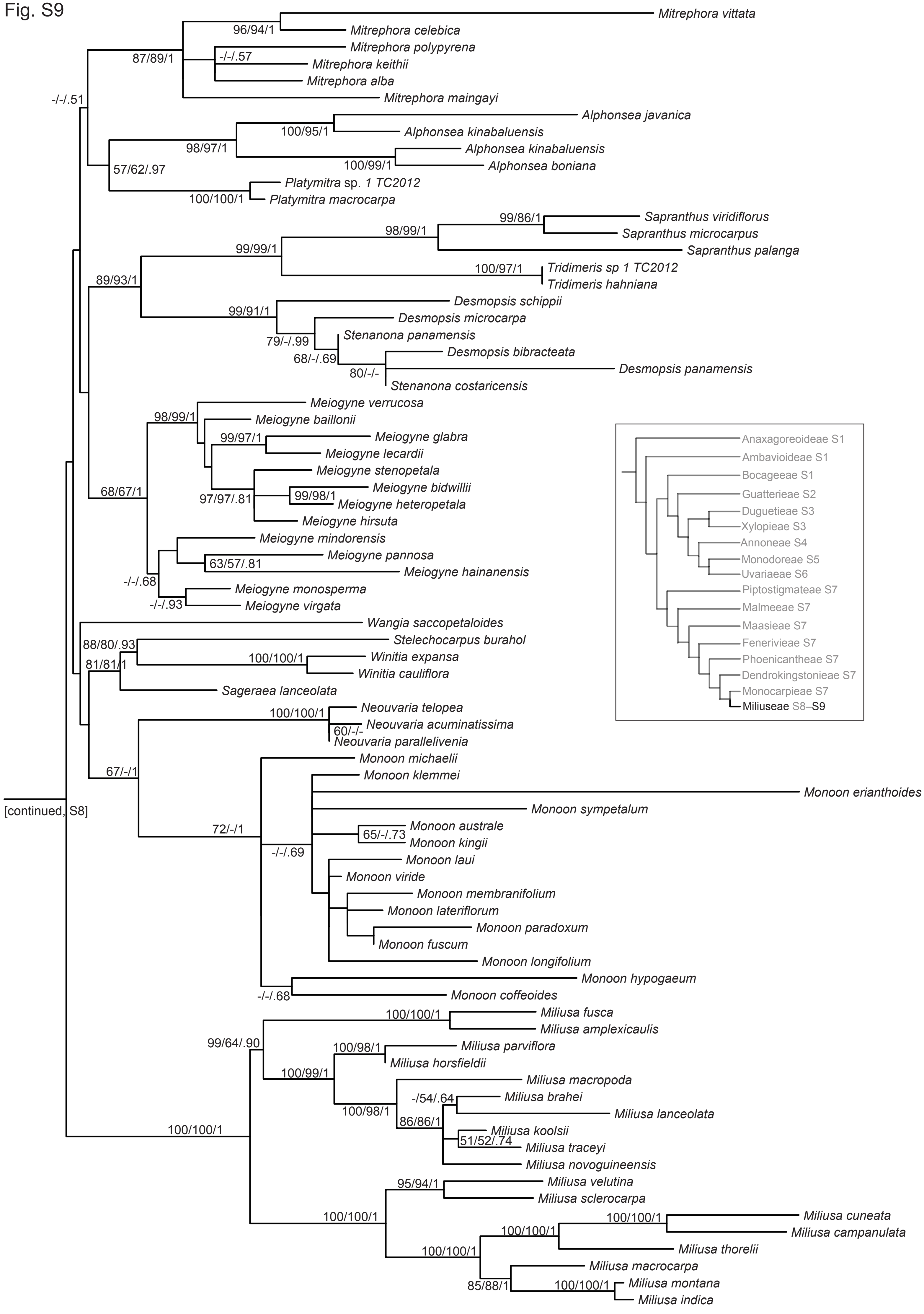
Mitrephora vittata
96/94/1
Mitrephora celebica
Mitrephora polypyrena
87/89/1
-/-/.57
Mitrephora keithii
Mitrephora alba
Mitrephora maingayi
-/-/.51
Alphonsea javanica
100/95/1
Alphonsea kinabaluensis
98/97/1
Alphonsea kinabaluensis
100/99/1
Alphonsea boniana
57/62/.97
Platymitra sp. 1 TC2012
100/100/1
Platymitra macrocarpa
99/86/1
Sapranthus viridiflorus
98/99/1
Sapranthus microcarpus
99/99/1
Sapranthus palanga
100/97/1
Tridimeris sp 1 TC2012
89/93/1
Tridimeris hahniana
Desmopsis schippii
99/91/1
Desmopsis microcarpa
79/-/.99
Stenanona panamensis
68/-/.69
Desmopsis bibracteata
80/-/-
Desmopsis panamensis
Stenanona costaricensis
Meiogyne verrucosa
98/99/1
Meiogyne baillonii
99/97/1
Meiogyne glabra
Meiogyne lecardii
Meiogyne stenopetala
68/67/1
Meiogyne bidwillii
99/98/1
97/97/.81
Meiogyne heteropetala
Meiogyne hirsuta
Meiogyne mindorensis
Meiogyne pannosa
63/57/.81
-/-/.68
Meiogyne hainanensis
Meiogyne monosperma
-/-/.93
Meiogyne virgata
Wangia saccopetaloides
88/80/.93
Stelechocarpus burahol
100/100/1
Winitia expansa
81/81/1
Winitia cauliflora
Sageraea lanceolata
100/100/1
Neouvaria telopea
60/-/-
Neouvaria acuminatissima
Neouvaria parallelivenia
Monoon michaelii
67/-/1
Monoon klemmei
Monoon erianthoides
[continued, S8]
Monoon sympetalum
Monoon australe
72/-/1
65/-/.73
Monoon kingii
-/-/.69
Monoon laui
Monoon viride
Monoon membranifolium
Monoon lateriflorum
Monoon paradoxum
Monoon fuscum
Monoon longifolium
Monoon hypogaeum
-/-/.68
Monoon coffeoides
100/100/1
Miliusa fusca
Miliusa amplexicaulis
99/64/.90
100/98/1
Miliusa parviflora
Miliusa horsfieldii
100/99/1
Miliusa macropoda
-/54/.64
Miliusa brahei
100/98/1
Miliusa lanceolata
86/86/1
Miliusa koolsii
100/100/1
51/52/.74
Miliusa traceyi
Miliusa novoguineensis
95/94/1
Miliusa velutina
Miliusa sclerocarpa
100/100/1
100/100/1
Miliusa cuneata
100/100/1
Miliusa campanulata
100/100/1
Miliusa thorelii
Miliusa macrocarpa
85/88/1
100/100/1
Miliusa montana
Miliusa indica
Anaxagoreoideae S1
Ambavioideae S1
Bocageeae S1
Guatterieae S2
Duguetieae S3
Xylopieae S3
Annoneae S4
Monodoreae S5
Uvariaeae S6
Piptostigmateae S7
Malmeeae S7
Maasieae S7
Fenerivieae S7
Phoenicantheae S7
Dendrokingstonieae S7
Monocarpieae S7
Miliuseae S8–S9

Supplementary Appendix I. Voucher information of newly generated accessions in this study.

**Species**	**Voucher**	**Origin**
Bocagea longepedunculata Mart. _Blo181	*R. de Mello-Silva 1181* (NY)	Brazil: Espírito Santo.
Bocagea longepedunculata Mart. _Blo460	*J. R. Pirani 3460* (NY)	Brazil: Espírito Santo.
Bocagea viridis A. St.-Hil.	*R. de Mello-Silva 1226* (NY)	Brazil: Rio de Janeiro.
Boutiquea platypetala (Engl. & Diels) Le Thomas	*M. F. de Carvalho 3931* (L)	Equatorial Guinea
Cardiopetalum calophyllum Schltdl.	*D. Alvarenga 634* (L)	Brazil: Distrito Federal
Cardiopetalum surinamense R. E. Fr.	*G. Deward 206* (L)	French Guiana: Gregoire
Duckeanthus grandiflorus R. E. Fr.	*P. J. M. Maas 6778* (L)	Brazil: Amazonas
Pseudephedranthus fragrans (R. E. Fr.) Aristeg._Pfa683	*N. T. da Silva 60683* (NY)	Brazil: Amazonas
Pseudephedranthus fragrans (R. E. Fr.) Aristeg._Pfa878	*P. J. M. Maas 6878* (NY)	Brazil: Amazonas
Phoenicanthus obliquus (Hook. f. & Thomson) Alston	*H. F. J. Huber 518* (L)	Sri Lanka: Ratnapura

+ Supplementary Appendix II. Species names and GenBank accession numbers of DNA sequences used in this study. -: missing data; *: newly generated sequences.

No.	Voucher information	GenBank accession numbers							
	Taxa name	*atpB-rbcL*	*matK*	*ndhF*	*psbA-trnH*	*rbcL*	*trnL-F*	*trnS-G*	*ycf1*
1	*Afroguatteria bequaertii* (De Wild.) Boutique	-	KX786588	-	-	KX786627	KX786629	-	-
2	*Alphonsea boniana* Finet & Gagnep.	-	AY518809	JQ723785	-	-	AY319077	-	-
3	*Alphonsea elliptica* Hook. f. & Thomson	-	AY518807	JQ690401	JQ690402	-	AY319078	-	JQ690403
4	*Alphonsea javanica* Scheff.	-	AY518810	-	-	-	AY319079	-	-
5	*Alphonsea kinabaluensis* J. Sinclair	-	AY518811	-	-	-	AY319080	-	-
6	*Ambavia gerrardii* (Baill.) Le Thomas	AY578118	AY220435	AY218168	-	JQ513886	JQ513889	-	-
7	*Anaxagorea acuminata* (Dunal) A. DC.	AY578121	AY220436	AY218169	-	-	-	-	-
8	*Anaxagorea crassipetala* Hemsl.	AY578126	JQ586481	-	-	JQ590151	AY580043	-	-
9	*Anaxagorea luzonensis* A. Gray	AY578132	AY518883	-	-	-	AY319188	-	-
10	*Anaxagorea panamensis* Standl.	AY578135	GQ981933	-	GQ982144	GQ981662	AY580052	-	-
11	*Anaxagorea phaeocarpa* Mart.	EF179244	AY238960	EF179279	AY841426	AY238952	EF179316	EF179321	-
12	*Anaxagorea silvatica* R. E. Fr.	EF179245	AY743477	EF179280	AY841427	AY743439	AY743458	EF179322	-
13	*Annickia affinis* (Exell) Versteegh & Sosef	AY841370	AY841393	AY841401	KC667934	AY841594	AY841671	AY841550	-
14	*Annickia kummeriae* (Engl. & Diels) Setten & Maas	-	AY238961	-	AY841443	AY238959	AY319171	-	-
15	*Annickia pilosa* (Exell) Setten & Maas	AY841371	AY743488	AY841402	AY841444	AY743450	AY743469	AY841551	-
16	*Annona acuminata* Saff.	-	GQ981934	-	GQ982146	GQ981664	-	-	-
17	*Annona chrysocarpa* Lepr. ex A. Rich.	-	-	-	-	EU420868	EU420850	-	-
18	*Annona cuspidata* (Mart.) H. Rainer	-	-	-	-	EU420869	EU420851	-	-
19	*Annona dumetorum* R. E. Fr.	-	GQ139704	-	GQ139844	EU420856	EU420838	-	GU937352

No.	Voucher information	GenBank accession numbers							
	Taxa name	*atpB-rbcL*	*matK*	*ndhF*	*psbA-trnH*	*rbcL*	*trnL-F*	*trnS-G*	*ycf1*
20	*Annona edulis* (Triana & Planch.) H. Rainer	-	-	-	-	AY841655	AY841733	-	-
21	*Annona glabra* L.	EF179246	GQ139717	EF179281	DQ125116	AY841596	AY841673	EF179323	GU937365
22	*Annona herzogii* (R. E. Fr.) H. Rainer	EF179273	DQ125062	EF179308	DQ125132	AY841656	AY841734	EF179350	-
23	*Annona membranacea* R. E. Fr.	-	JQ586514	-	-	JQ590184	-	-	-
24	*Annona mucosa* Jacq.	-	GQ139705	-	GQ139845	EU420870	EU420852	-	GU937353
25	*Annona muricata* L.	EF179247	AF543722	EF179282	AY841428	AY743440	AY743459	EF179324	-
26	*Annona reticulata* L.	-	JQ586491	-	JX856821	EU420863	EU420845	-	-
27	*Annona sclerophylla* Saff.	-	GQ139718	-	GQ139858	-	GQ139892	-	GU937366
28	*Annona spraguei* Saff.	-	GQ981935	-	GQ982147	GQ981665	-	-	-
29	*Annona squamosa* L.	-	EU715064	-	EU715086	EU420865	EU420847	-	-
30	*Anonidium mannii* (Oliv.) Engl. & Diels	EF179248	DQ125051	EF179283	DQ125117	AY841598	AY841675	EF179325	-
31	*Artabotrys brachypetalus* Benth.	-	-	-	-	JF265293	-	-	-
32	*Artabotrys hexapetalus* (L. f.) Bhandari	EF179249	AY238962	EF179284	AY841429	AY238953	EF179317	EF179326	-
33	*Artabotrys thomsonii* Oliv.	EF179250	DQ125052	EF179285	DQ125118	AY841599	AY841676	EF179327	-
34	*Asimina incana* (W. Bartram) Exell	-	GQ139730	-	GQ139873	-	GQ139903	-	GU937377
35	*Asimina longifolia* Kral	EF179251	GQ139707	EF179286	DQ125119	DQ124939	GQ139885	EF179328	GU937355
36	*Asimina obovata* (Willd.) Nash	-	GQ139716	-	GQ139855	-	GQ139908	-	GU937363
37	*Asimina parviflora* (Michx.) Dunal	-	GQ139703	-	GQ139843	-	GQ139878	-	GU937351
38	*Asimina pulchellus* (Small) Rehder & Dayton	-	GQ139714	-	GQ139854	-	GQ139889	-	GU937362
39	*Asimina pygmaea* (W. Bartram) Dunal	-	GQ139726	-	GQ139868	-	GQ139899	-	GU937356
40	*Asimina reticulata* Shuttlew. ex Chapm.	-	GQ139713	-	GQ139863	-	GQ139906	-	GU937361
41	*Asimina rugelii* B. L. Rob.	-	GQ139706	-	GQ139846	JQ513887	GQ139881	-	GU937354
42	*Asimina tetramera* Small	-	GQ139702	-	GQ139870	-	GQ139877	-	GU937350

No.	Voucher information	GenBank accession numbers							
	Taxa name	*atpB-rbcL*	*matK*	*ndhF*	*psbA-trnH*	*rbcL*	*trnL-F*	*trnS-G*	*ycf1*
43	*Asimina triloba* (L.) Dunal	EF179252	GQ139711	AY218171	AY841430	AY743441	AY743460	EF179329	GU937349
44	*Asteranthe asterias* (S. Moore) Engl. & Diels	-	-	EU169711	EU169734	EU169757	EU169779	EU169801	-
45	*Bocagea longepedunculata* Mart. Blo181	MF322668*	MF322630*	MF322640*	MF322655*	MF322645*	MF322659*	-	-
46	*Bocagea longepedunculata* Mart. Blo460	MF322669*	MF322631*	MF322641*	-	MF322646*	-	-	-
47	*Bocagea viridis* A. St.-Hil.	MF322670*	MF322632*	MF322642*	-	MF322647*	MF322660*	-	-
48	*Bocageopsis canescens* (Spruce ex Benth.) R. E. Fr.	-	JQ690409	JQ690410	JQ690411	JQ690407	JQ690408	-	JQ690412
49	*Bocageopsis multiflora* (Mart.) R. E. Fr.	-	DQ018262	-	AY841445	AY841600	AY841678	-	-
50	*Bocageopsis pleiosperma* Maas	-	-	-	AY841446	AY841601	AY841679	-	-
51	*Boutiquea platypetala* (Engl. & Diels) Le Thomas	MF322671*	MF322633*	MF322643*	-	MF322648*	MF322661*	-	-
52	*Cananga brandisiana* (Pierre) I. M. Turner	-	HM173744	-	HM173715	HM173801	HM173772	-	-
53	*Cananga odorata* (Lam.) Hook. f. & Thomson	AY841372	AY220438	AY841403	AY841431	AY841602	AY841680	AY841548	-
54	*Cardiopetalum calophyllum* Schltdl.	MF322672*	MF322634*	MF322644*	-	MF322649*	MF322662*	-	-
55	*Cardiopetalum surinamense* R. E. Fr.	MF322673*	MF322635*	-	-	MF322650*	MF322663*	-	-
56	*Cleistochlamys kirkii* (Benth.) Oliv.	-	-	KM924880	KM924981	-	KM924948	-	-
57	*Cleistopholis glauca* Pierre ex Engl. & Diels	AY841373	AY841395	AY841404	AY841432	AY841603	AY841681	AY841549	-
58	*Coelocaryon preussii* Warb.	-	AY743475	JQ437546	-	AY743437	AY743456	-	-
59	*Cremastosperma brevipes* (DC.) R. E. Fr.	AY841374	AY743550	AY841405	AY841447	AY743527	AY743573	AY841552	-
60	*Cremastosperma bullatum* Pirie	-	AY743560	DQ018140	AY841459	DQ018235	DQ018188	-	-
61	*Cremastosperma cauliflorum* R. E. Fr.	AY841375	AY743542	AY841406	DQ018240	AY743519	AY743565	AY841553	-
62	*Cremastosperma cenepense* Pirie & Zapata	-	DQ018277	-	DQ018257	DQ018236	-	-	-
63	*Cremastosperma gracilipes* R. E. Fr.	-	AY743544	-	-	AY743521	AY743567	-	-

No.	Voucher information	GenBank accession numbers							
	Taxa name	*atpB-rbcL*	*matK*	*ndhF*	*psbA-trnH*	*rbcL*	*trnL-F*	*trnS-G*	*ycf1*
64	*Cremastosperma leiophyllum* (Diels) R. E. Fr.	-	AY743546	DQ018123	AY841449	AY743523	AY743569	-	-
65	*Cremastosperma macrocarpum* Maas	-	AY743551	DQ018129	AY841450	AY743528	AY743574	-	-
66	*Cremastosperma magdalenae* Pirie	-	DQ018279	DQ018143	AY841460	AY841521	AY841535	-	-
67	*Cremastosperma megalophyllum* R. E. Fr.	-	AY743545	DQ018121	AY841451	AY743517	AY743563	-	-
68	*Cremastosperma microcarpum* R. E. Fr.	-	AY743539	DQ018120	AY841452	AY743516	AY319172	-	-
69	*Cremastosperma monospermum* (Rusby) R. E. Fr.	-	AY743547	DQ018124	AY841453	AY743524	AY743570	-	-
70	*Cremastosperma napoense* Pirie	-	DQ018265	DQ018127	DQ018242	DQ018224	DQ018177	-	-
71	*Cremastosperma novogranatense* R. E. Fr.	-	AY743552	-	-	AY743529	AY743575	-	-
72	*Cremastosperma oblongum* R. E. Fr.	-	DQ018266	DQ018128	DQ018241	DQ018225	DQ018178	-	-
73	*Cremastosperma panamense* Maas	-	AY743553	DQ018131	DQ018246	AY743530	AY743576	-	-
74	*Cremastosperma pedunculatum* (Diels) R. E. Fr.	-	DQ018269	DQ018135	DQ018248	DQ018228	DQ018181	-	-
75	*Cremastosperma pendulum* (Ruiz & Pav.) R. E. Fr.	-	AY743554	DQ018132	DQ018247	DQ018233	DQ018186	-	-
76	*Cremastosperma peruvianum* R. E. Fr.	-	AY743557	-	-	AY743534	AY743580	-	-
77	*Cremastosperma venezuelanum* Pirie	-	AY743559	DQ018134	AY841457	AY743536	AY743582	-	-
78	*Cremastosperma westrae* Pirie	-	DQ018272	DQ018136	DQ018252	DQ018231	DQ018184	-	-
79	*Cremastosperma yamayakatense* Pirie	-	DQ018267	DQ018139	DQ018244	DQ018226	DQ018179	-	-
80	*Cyathocalyx annamensis* Ast	-	HM173748	-	HM173719	HM173805	HM173776	-	-
81	*Cyathocalyx globosus* Merr.	-	HM173725	-	HM173696	HM173782	HM173754	-	-
82	*Cyathocalyx harmandii* (Finet & Gagnep.) R. J. Wang & R. M. K. Saunders	-	HM173726	-	HM173697	HM173783	HM173755	-	-

No.	Voucher information	GenBank accession numbers							
	Taxa name	*atpB-rbcL*	*matK*	*ndhF*	*psbA-trnH*	*rbcL*	*trnL-F*	*trnS-G*	*ycf1*
83	*Cyathocalyx magnifructus* R. J. Wang & R. M. K. Saunders	-	HM173730	-	HM173701	HM173787	HM173759	-	-
84	*Cyathocalyx martabanicus* Hook. f. & Thomson	EF179253	DQ125054	EF179288	DQ125120	AY841605	AY841683	EF179330	-
85	*Cyathocalyx sumatranus* Scheff.	-	HM173737	-	HM173708	HM173794	HM173766	-	-
86	*Cyathocalyx zeylanicus* Champ. ex Hook. f. & Thomson	-	HM173739	-	HM173710	HM173796	HM173768	-	-
87	*Cymbopetalum brasiliense* (Vell.) Benth. ex Baill.	EF179254	DQ125055	EF179289	DQ125121	AY841608	AY841686	EF179331	-
88	*Cymbopetalum torulosum* G. E. Schatz	-	GQ139720	-	GQ139859	AY743442	GQ139894	-	-
89	*Dasymaschalon acuminatum* Jing Wang & R. M. K. Saunders	-	JQ768546	JQ768587	JQ768625	JQ768666	JQ768706	-	-
90	*Dasymaschalon borneense* Nurmawati	-	JQ768547	-	JQ768626	JQ768667	JQ768707	-	-
91	*Dasymaschalon clusiflorum* (Merr.) Merr.	-	JQ768548	-	JQ768627	JQ768668	JQ768708	-	-
92	*Dasymaschalon dasymaschalum* (Blume) I. M. Turner	-	JQ768549	JQ768588	JQ768628	JQ768669	JQ768709	-	-
93	*Dasymaschalon ellipticum* Nurmawati	-	JQ768550	JQ768589	JQ768629	JQ768670	JQ768710	-	-
94	*Dasymaschalon evrardii* Ast	-	JQ768551	JQ768590	JQ768630	JQ768671	JQ768711	-	-
95	*Dasymaschalon glaucum* Merr. & Chun	-	JQ768553	JQ768592	JQ768632	JQ768673	JQ768713	-	-
96	*Dasymaschalon lomentaceum* Finet & Gagnep.	-	EU715065	JQ768593	EU715087	JQ768674	JQ768714	-	-
97	*Dasymaschalon longiusculum* (Bân) Jing Wang & R. M. K. Saunders	-	JQ768556	-	JQ768635	JQ768676	JQ768716	-	-
98	*Dasymaschalon macrocalyx* Finet & Gagnep.	EF179255	EF179277	EF179290	EF179313	AY841610	AY841688	EF179332	-

No.	Voucher information	GenBank accession numbers							
	Taxa name	*atpB-rbcL*	*matK*	*ndhF*	*psbA-trnH*	*rbcL*	*trnL-F*	*trnS-G*	*ycf1*
99	*Dasymaschalon megalanthum* (Merr.) Jing Wang & R.M.K. Saunders	-	JQ768558	JQ768596	JQ768637	JQ768678	JQ768718	-	-
100	*Dasymaschalon oblongatum* Merr.	-	JQ768559	JQ768597	JQ768638	JQ768679	JQ768719	-	-
101	*Dasymaschalon robinsonii* Ast	-	JQ768561	-	JQ768640	JQ768681	JQ768721	-	-
102	*Dasymaschalon rostratum* Merr. & Chun	-	JQ768562	JQ768599	JQ768641	JQ768682	JQ768722	-	-
103	*Dasymaschalon sootepense* Craib	-	AY743481	JQ768600	JQ768642	AY743443	AY743462	-	-
104	*Dasymaschalon trichophorum* Merr.	-	JQ768565	JQ768602	JQ768644	JQ768685	JQ768725	-	-
105	*Dasymaschalon wallichii* (Hook. f. & Thomson) Jing Wang & R. M. K. Saunders	-	JQ768566	-	JQ768645	JQ768686	JQ768726	-	-
106	*Dasymaschalon yunnanense* (Hu) Bân	-	JQ768560	JQ768598	JQ768639	JQ768680	JQ768720	-	-
107	*Dendrokingstonia gardneri* Chaowasku	-	KJ418391	KJ418385	KJ418399	KJ418381	KJ418406	-	KJ418378
108	*Dendrokingstonia nervosa* (Hook. f. & Thomson) Rauschert	-	KJ418392	KJ418386	KJ418400	KJ418382	KJ418407	-	-
109	*Desmopsis bibracteata* (B. L. Rob.) Saff.	-	JQ586480	-	-	JQ590148	-	-	-
110	*Desmopsis microcarpa* R. E. Fr.	-	AY518804	JX544771	AY841461	-	AY319173	-	JX544758
111	*Desmopsis panamensis* (B. L. Rob.) Saff.	-	GQ981981	-	GQ982207	GQ981723	-	-	-
112	*Desmopsis schippii* Standl.	-	AY518805	JQ723786	-	JQ590166	AY319174	-	-
113	*Desmos chinensis* Lour.	-	JQ768567	JQ768603	JQ768646	JQ762414	JQ762415	-	-
114	*Desmos cochinchinensis* Lour.	-	JQ768568	JQ768604	JQ768647	JQ768688	JQ768728	-	-
115	*Desmos dinhensis* (Finet & Gagnep.) Merr.	-	JQ768569	JQ768605	JQ768648	-	JQ768729	-	-
116	*Desmos dumosus* (Roxb.) Saff.	-	JQ768570	JQ768606	JQ768649	JQ768689	JQ768730	-	-
117	*Desmos elegans* (Thwaites) Saff.	-	JQ768571	-	JQ768650	JQ768690	JQ768731	-	-
118	*Desmos goezeanus* (F. Muell.) Jessup	-	JQ768572	JQ768607	JQ768651	JQ768691	JQ768732	-	-

No.	Voucher information	GenBank accession numbers							
	Taxa name	*atpB-rbcL*	*matK*	*ndhF*	*psbA-trnH*	*rbcL*	*trnL-F*	*trnS-G*	*ycf1*
119	*Desmos wardianus* (F. M. Bailey) Jessup	-	JQ768574	JQ768608	JQ768653	JQ768693	JQ768734	-	-
120	*Diclinanona tessmannii* Diels	-	DQ125056	-	-	AY841611	AY841689	-	-
121	*Dielsiothamnus divaricatus* (Diels) R. E. Fr.	-	-	-	EU169736	EU169759	EU169781	EU169803	-
122	*Disepalum aciculare* D. M. Johnson	-	KT452821	KT452832	-	-	KT452843	-	KT452854
123	*Disepalum anomalum* Hook. f.	-	KT452819	KT452830	-	-	KT452841	-	KT452852
124	*Disepalum coronatum* Becc.	-	KT452820	KT452831	-	-	KT452842	-	KT452853
125	*Disepalum longipes* King	-	KT452822	KT452833	-	-	KT452844	-	KT452855
126	*Disepalum petelotii* (Merr.) D. M. Johnson	-	KT452813	KT452824	-	-	KT452835	-	KT452846
127	*Disepalum plagioneurum* (Diels) D. M. Johnson	-	KT452814	KT452825	-	-	KT452836	-	KT452847
128	*Disepalum platypetalum* Merr.	EF179257	DQ125057	EF179292	DQ125122	-	-	EF179334	-
129	*Disepalum pulchrum* (King) J. Sinclair	-	GQ139736	-	GQ139875	JQ513888	GQ139909	-	GU937383
130	*Drepananthus apoensis* Elmer	-	HM173721	-	HM173692	HM173778	HM173750	-	-
131	*Drepananthus biovulatus* (Boerl.) Survesw. & R. M. K. Saunders	-	HM173722	-	HM173693	AY841604	AY841682	-	-
132	*Drepananthus cauliflorus* (Lauterb. & K. Schum.) Survesw. & R. M. K. Saunders	-	HM173723	-	HM173694	HM173780	HM173752	-	-
133	*Drepananthus deltoideus* (Airy Shaw) Survesw. & R. M. K. Saunders	-	HM173724	-	HM173695	HM173781	HM173753	-	-
134	*Drepananthus havilandii* (Boerl.) Survesw. & R. M. K. Saunde*rs*	-	HM173727	-	HM173698	HM173784	HM173756	-	-
135	*Drepananthus hexagynus* (Miq.) Survesw. & R. M. K. Saunders	-	HM173740	-	HM173711	HM173797	HM173769	-	-

No.	Voucher information	GenBank accession numbers							
	Taxa name	*atpB-rbcL*	*matK*	*ndhF*	*psbA-trnH*	*rbcL*	*trnL-F*	*trnS-G*	*ycf1*
136	*Drepananthus kingii* (Boerl. ex Koord.) Survesw. & R. M. K. Saunders	-	HM173728	-	HM173699	HM173785	HM173757	-	-
137	*Drepananthus magnificus* (Diels) Survesw. & R. M. K. Saunders	-	HM173729	-	HM173700	HM173786	HM173758	-	-
138	*Drepananthus obtusifolius* (Becc. & Scheff.) Survesw. & R. M. K. Saunders	-	HM173732	-	HM173703	HM173789	HM173761	-	-
139	*Drepananthus pahangensis* M. R. Hend.	-	HM173741	-	HM173712	HM173798	-	-	-
140	*Drepananthus petiolatus* (Diels) Survesw. & R. M. K. Saunders	-	HM173743	-	HM173714	HM173800	HM173771	-	-
141	*Drepananthus polycarpus* (C. T. White & W. D. Francis) Survesw. & R. M. K. Saunders	-	HM173733	-	HM173704	HM173790	HM173762	-	-
142	*Drepananthus pruniferus* Maingay ex Hook. f. & Thomson	-	HM173734	-	HM173705	HM173791	HM173763	-	-
143	*Drepananthus pubescens* (Scheff.) Survesw. & R. M. K. Saunders	-	HM173735	-	HM173706	HM173792	HM173764	-	-
144	*Drepananthus ridleyi* (King) Survesw. & R. M. K. Saunders	-	HM173736	-	HM173707	HM173793	HM173765	-	-
145	*Drepananthus vitiensis* (A. C. Sm.) Survesw. & R. M. K. Saunders	-	HM173738	-	HM173709	HM173795	HM173767	-	-
146	*Duckeanthus grandiflorus* R. E. Fr.	MF322674*	MF322636*	-	-	MF322651*	MF322664*	-	-
147	*Duguetia bahiensis* Maas	-	AY740532	-	-	AY738152	AY740564	-	-
148	*Duguetia cadaverica* Huber	-	AY740533	-	-	AY738153	AY740565	-	-
149	*Duguetia calycina* Benoist	-	AY740534	-	-	AY738154	AY740566	-	-
150	*Duguetia cauliflora* R. E. Fr.	-	AY740535	-	-	AY738155	AY740567	-	-

No.	Voucher information	GenBank accession numbers							
	Taxa name	*atpB-rbcL*	*matK*	*ndhF*	*psbA-trnH*	*rbcL*	*trnL-F*	*trnS-G*	*ycf1*
151	*Duguetia chrysea* Maas	-	AY740536	-	AY841435	AY738156	AY841691	-	-
152	*Duguetia confinis* (Engl. & Diels) Chatrou	-	AY740537	-	-	AY738157	AY740569	-	-
153	*Duguetia confusa* Maas	-	AY740538	-	-	AY738158	AY740570	-	-
154	*Duguetia echinophora* R. E. Fr.	-	AY740539	-	-	AY738159	AY740571	-	-
155	*Duguetia guianensis* R. E. Fr.	-	AY740540	-	-	AY738160	AY740572	-	-
156	*Duguetia hadrantha* (Diels) R. E. Fr.	EF179258	AY740541	EF179293	DQ125123	AY738161	AY740573	EF179335	-
157	*Duguetia lucida* Urb.	-	AY740542	-	-	AY738162	AY740574	-	-
158	*Duguetia macrocalyx* R. E. Fr.	-	AY740543	-	-	AY738163	AY740575	-	-
159	*Duguetia macrophylla* R. E. Fr.	-	AY740544	-	-	AY738164	AY740576	-	-
160	*Duguetia marcgraviana* Mart.	-	AY740545	-	-	AY738165	AY740577	-	-
161	*Duguetia megalocarpa* Maas	-	AY740546	-	-	AY738166	AY740578	-	-
162	*Duguetia moricandiana* Mart.	-	AY740547	-	-	AY738167	AY740579	-	-
163	*Duguetia neglecta* Sandwith	-	AY740548	-	-	AY738168	AY740580	-	-
164	*Duguetia odorata* (Diels) J. F. Macbr.	-	AY740549	-	-	AY738169	AY740581	-	-
165	*Duguetia panamensis* Standl.	-	AY740550	-	-	AY738170	AY740582	-	-
166	*Duguetia peruviana* (R. E. Fr.) J. F. Macbr.	-	AY740551	-	-	AY738171	AY740583	-	-
167	*Duguetia pycnastera* Sandwith	-	AY740552	-	-	AY738172	AY740584	-	-
168	*Duguetia quitarensis* Benth.	-	AY740553	-	-	AY738173	AY740585	-	-
169	*Duguetia riedeliana* R. E. Fr.	-	AY740554	-	-	AY738174	AY740586	-	-
170	*Duguetia salicifolia* R. E. Fr.	-	AY740555	-	-	AY738175	AY740587	-	-
171	*Duguetia sessilis* (Vell.) Maas	-	AY740556	-	-	AY738176	AY740588	-	-
172	*Duguetia sooretamae* Maas	-	AY740557	-	DQ861746	AY738177	AY740589	-	-
173	*Duguetia staudtii* (Engl. & Diels) Chatrou	EF179259	AY740558	EF179294	DQ125124	AY738178	AY740590	EF179336	-

No.	Voucher information	GenBank accession numbers							
	Taxa name	*atpB-rbcL*	*matK*	*ndhF*	*psbA-trnH*	*rbcL*	*trnL-F*	*trnS-G*	*ycf1*
174	*Duguetia stelechantha* (Diels) R. E. Fr.	-	AY740559	-	-	AY738179	AY740591	-	-
175	*Duguetia surinamensis* R. E. Fr.	-	AY740560	-	FJ038842	AY738180	AY740592	-	-
176	*Duguetia ulei* (Diels) R. E. Fr.	-	AY740561	-	-	AY738181	AY740593	-	-
177	*Duguetia uniflora* (DC.) Mart.	-	AY740562	-	-	AY738182	AY740594	-	-
178	*Duguetia yeshidan* Sandwith	-	AY740563	-	-	AY738183	AY740595	-	-
179	*Ephedranthus boliviensis* Chatrou & Pirie	-	-	-	-	AY841614	AY841692	-	-
180	*Ephedranthus parviflorus* S. Moore	-	-	-	AY841462	AY841615	AY841693	-	-
181	*Ephedranthus sp Maas et al 8826*	AY841376	AY841396	AY841407	AY841463	AY841616	AY841694	AY841554	-
182	*Eupomatia bennettii* F.Müll.	-	JQ437547	-	-	DQ861790	DQ861842	JQ513885	-
183	*Fenerivia angustielliptica* (G. E. Schatz & Le Thomas) R. M. K. Saunders	-	JF810373	-	-	JF810385	JF810397	-	-
184	*Fenerivia capuronii* (Cavaco & Keraudren) R. M. K. Saunders	-	JF810374	-	-	JF810386	JF810398	-	-
185	*Fenerivia chapelieri* (Baill.) R. M. K. Saunders	-	JF810375	JQ723788	-	JF810387	JF810399	-	-
186	*Fenerivia emarginata* (Diels) R. M. K. Saunders	-	JF810376	-	-	JF810388	JF810400	-	-
187	*Fenerivia ghesquiereana* (Cavaco & Keraudren) R. M. K. Saunders	-	JF810377	-	-	JF810389	JF810401	-	-
188	*Fenerivia heteropetala* Diels	-	JF810378	-	-	JF810390	JF810402	-	-
189	*Fenerivia humbertii* (Diels) R. M. K. Saunders	-	JF810379	-	-	JF810391	JF810403	-	-
190	*Fenerivia madagascariensis* (Cavaco & Keraudren) R. M. K. Saunders	-	JF810380	-	-	JF810392	JF810404	-	-
191	*Fenerivia oligosperma* (Danguy) R. M. K. Saunders	-	JF810381	-	-	JF810393	JF810405	-	-

No.	Voucher information	GenBank accession numbers							
	Taxa name	*atpB-rbcL*	*matK*	*ndhF*	*psbA-trnH*	*rbcL*	*trnL-F*	*trnS-G*	*ycf1*
192	*Fenerivia richardiana* (Baill.) R. M. K. Saunders	-	JF810382	-	-	JF810394	JF810406	-	-
193	*Fissistigma glaucescens* (Hance) Merr.	-	AY743482	-	-	AY743444	AY743463	-	-
194	*Fissistigma polyanthoides* (Aug. DC.) Merr.	-	JQ768575	JQ768609	JQ768654	JQ768694	JQ768735	-	-
195	*Fissistigma uonicum* (Dunn) Merr.	-	-	-	-	AY841617	AY841695	-	-
196	*Friesodielsia biglandulosa* (Blume) Steenis	-	-	JQ768610	JQ768655	-	JQ768736	-	-
197	*Friesodielsia cuneiformis* (Blume) Steenis	-	JQ768576	JQ768611	-	JQ768695	JQ768737	-	-
198	*Friesodielsia desmoides* (Craib) Steenis	-	JQ768577	JQ768612	JQ768656	JQ768696	AY841696	-	-
199	*Fusaea longifolia* (Aubl.) Saff.	-	FJ514750	-	FJ038844	AY841620	AY841698	-	-
200	*Fusaea peruviana* R. E. Fr.	EF179260	AY743483	EF179295	AY841436	AY743445	AY743464	EF179337	-
201	*Goniothalamus amuyon* (Blanco) Merr.	KM818567	KM818518	KM818648	KM818728	KM818839	KM818898	KM81891 6	KM81897 9
202	*Goniothalamus andersonii* J. Sinclair	KM818519	KM818568	-	KM818711	KM818789	KM818867	KM81894 9	-
203	*Goniothalamus angustifolius* (A. C. Sm.) B. Xue & R. M. K. Saunders	-	KM818569	KM818632	KM818732	KM818797	KM818878	KM81893 7	KM81898 3
204	*Goniothalamus aruensis* Scheff.	KM818520	KM818570	KM818640	KM818706	KM818791	KM818868	KM81891 8	-
205	*Goniothalamus australis* Jessup	KM818521	KM818571	KM818638	KM818709	KM818836	KM818887	KM81891 0	KM81897 3
206	*Goniothalamus borneensis* Mat-Salleh	KM818522	KM818572	KM818673	-	KM818826	KM818893	KM81895 2	-
207	*Goniothalamus bracteosus* Bân	-	KM818573	-	KM818730	KM818796	KM818906	KM81896 7	-

No.	Voucher information	GenBank accession numbers							
	Taxa name	*atpB-rbcL*	*matK*	*ndhF*	*psbA-trnH*	*rbcL*	*trnL-F*	*trnS-G*	*ycf1*
208	*Goniothalamus calcareus* Mat-Salleh	-	-	-	KM818717	KM818810	-	KM818927	KM818994
209	*Goniothalamus calvicarpus* Craib	KM818523	KM818574	KM818647	KM818702	KM818809	KM818874	KM818934	KM819005
210	*Goniothalamus cardiopetalus* (Dalzell) Hook. f. & Thomson	KM818524	KM818575	KM818654	KM818692	KM818799	KM818879	KM818912	-
211	*Goniothalamus cauliflorus* K. Schum.	KM818525	KM818576	KM818663	KM818696	KM818807	KM818869	KM818919	-
212	*Goniothalamus cheliensis* Hu	KM818526	KM818577	KM818661	KM818678	KM818831	KM818901	KM818926	KM818992
213	*Goniothalamus clemensii* Bân	-	KM818578	-	KM818736	KM818780	KM818844	KM818915	-
214	*Goniothalamus costulatus* Miq.	-	KM818579	-	KM818737	KM818805	KM818865	KM818945	-
215	*Goniothalamus dumontetii* R. M. K. Saunders & Munzinger	-	KM818580	-	KM818729	KM818840	KM818861	KM818954	-
216	*Goniothalamus elegans* Ast	KM818527	KM818581	KM818676	KM818707	KM818817	KM818850	KM818955	KM818997
217	*Goniothalamus elmeri* Merr.	-	KM818582	KM818639	KM818677	KM818811	KM818882	KM818924	KM819003
218	*Goniothalamus expansus* Craib	-	KM818583	KM818634	KM818714	KM818829	KM818853	KM818931	KM818987
219	*Goniothalamus fasciculatus* Boerl.	KM818528	KM818584	KM818636	-	-	KM818890	KM818950	-

No.	Voucher information	GenBank accession numbers							
	Taxa name	*atpB-rbcL*	*matK*	*ndhF*	*psbA-trnH*	*rbcL*	*trnL-F*	*trnS-G*	*ycf1*
220	*Goniothalamus gardneri* Hook. f. & Thomson	KM818529	KM818585	KM818656	KM818704	KM818784	KM818871	KM818923	KM819001
221	*Goniothalamus giganteus* Wall. ex Hook. f. & Thomson	KM818530	KM818586	KM818655	KM818698	KM818837	KM818892	KM818963	KM818996
222	*Goniothalamus grandiflorus* (Warb.) Boerl.	KM818531	KM818587	KM818637	KM818691	KM818802	KM818851	KM818930	-
223	*Goniothalamus griffithii* Hook. f. & Thomson	KM818532	KM818588	KM818651	KM818701	KM818798	KM818894	KM818939	KM819000
224	*Goniothalamus hookeri* Thwaites	KM818533	KM818589	KM818657	KM818734	KM818814	KM818872	KM818956	-
225	*Goniothalamus howii* Merr. & Chun	KM818534	KM818590	-	KM818689	KM818833	KM818886	KM818938	KM818986
226	*Goniothalamus imbricatus* (Blume) Koord.	KM818535	KM818591	-	KM818722	KM818806	KM818847	KM818946	KM818998
227	*Goniothalamus kinabaluensis* Bân ex Mat-Salleh	KM818536	KM818592	KM818672	KM818684	KM818787	KM818876	KM818935	-
228	*Goniothalamus laoticus* (Fin. & Gagnep.) Tien Ban	KM818537	KM818593	KM818666	KM818699	KM818808	KM818881	KM818959	KM818993
229	*Goniothalamus loerzingii* R. M. K. Saunders	-	KM818594	-	KM818724	KM818782	KM818902	KM818947	-
230	*Goniothalamus macranthus* (Kurz) Boerl.	KM818538	KM818595	KM818643	KM818695	KM818792	KM818873	KM818928	KM818995
231	*Goniothalamus macrophyllus* (Blume) Hook. f. & Thomson	KM818539	KM818596	KM818665	KM818688	KM818843	KM818897	KM818940	KM819002

No.	Voucher information	GenBank accession numbers							
	Taxa name	*atpB-rbcL*	*matK*	*ndhF*	*psbA-trnH*	*rbcL*	*trnL-F*	*trnS-G*	*ycf1*
232	*Goniothalamus maewongensis* R. M. K. Saunders & Chalermglin	KM818540	KM818597	KM818659	KM818725	KM818838	KM818888	KM81896 2	KM81897 7
233	*Goniothalamus majestatis* Keßler	KM818541	KM818598	-	KM818713	KM818788	KM818903	KM81895 8	-
234	*Goniothalamus malayanus* Hook. f. & Thomson	KM818542	KM818599	KM818650	KM818718	KM818835	KM818891	KM81891 4	KM81900 6
235	*Goniothalamus megalocalyx* I. M. Turner & R. M. K. Saunders	KM818543	KM818600	KM818645	KM818726	KM818822	KM818885	KM81896 0	KM81900 7
236	*Goniothalamus monospermus* (A. Gray) R. M. K. Saunders	-	KM818601	-	KM818735	KM818790	-	KM81896 9	-
237	*Goniothalamus montanus* J. Sinclair	KM818544	KM818602	KM818674	KM818710	KM818813	KM818856	KM81893 2	-
238	*Goniothalamus obtusatus* (Baill.) R. M. K. Saunders	KM818545	KM818603	KM818660	KM818687	KM818815	KM818883	KM81891 1	KM81898 1
239	*Goniothalamus palawanensis* C.C. Tang & R. M. K. Saunders	-	KM818604	-	KM818716	KM818793	KM818855	KM81892 5	KM81897 6
240	*Goniothalamus parallelivenius* Ridl.	KM818546	KM818605	KM818635	KM818683	KM818801	KM818880	KM81894 1	-
241	*Goniothalamus repevensis* Pierre ex Finet & Gagnep.	KM818547	KM818606	KM818664	KM818723	KM818795	KM818877	KM81893 6	-
242	*Goniothalamus reticulatus* Thwaites	KM818548	KM818607	-	-	KM818786	-	KM81891 3	-
243	*Goniothalamus ridleyi* King	KM818549	KM818608	-	KM818739	KM818830	KM818860	KM81895 1	KM81898 5

No.	Voucher information	GenBank accession numbers							
	Taxa name	*atpB-rbcL*	*matK*	*ndhF*	*psbA-trnH*	*rbcL*	*trnL-F*	*trnS-G*	*ycf1*
244	*Goniothalamus rotundisepalus* M. R. Hend.	KM818550	KM818609	KM818649	KM818693	KM818794	KM818857	KM818908	-
245	*Goniothalamus rufus* Miq.	KM818551	KM818610	-	KM818727	KM818819	KM818848	KM818943	-
246	*Goniothalamus sawtehii* C. E. C. Fisch.	KM818552	KM818611	KM818646	KM818680	KM818785	KM818895	KM818942	KM819004
247	*Goniothalamus scortechinii* King	KM818553	KM818612	KM818670	KM818712	KM818781	KM818845	KM818929	KM818988
248	*Goniothalamus sesquipedalis* (Wall.) Hook. f. & Thomson	KM818554	KM818613	KM818667	KM818719	KM818825	KM818904	KM818907	KM818984
249	*Goniothalamus* sp. tcc10	-	KM818614	KM818675	KM818715	KM818821	KM818864	KM818944	KM818980
250	*Goniothalamus suaveolens* Becc. 466	-	KM818615	-	KM818681	KM818818	KM818884	KM818968	KM818999
251	*Goniothalamus suaveolens* Becc. tcc32	KM818555	KM818616	-	KM818682	KM818800	KM818858	KM818933	KM818982
252	*Goniothalamus tamirensis* Pierre ex Finet & Gagnep.	KM818556	KM818617	KM818662	KM818700	KM818832	KM818866	KM818917	KM818990
253	*Goniothalamus tapis* Miq.	EF179262	DQ125058	EF179297	AY841622	AY841700	EF179339	-	AY841622
254	*Goniothalamus tapisoides* Mat-Salleh	KM818557	KM818618	KM818641	KM818686	KM818823	KM818899	KM818920	-
255	*Goniothalamus tavoyensis* Chatterjee	KM818558	KM818619	KM818633	KM818690	KM818841	KM818854	KM818961	-

No.	Voucher information	GenBank accession numbers							
	Taxa name	*atpB-rbcL*	*matK*	*ndhF*	*psbA-trnH*	*rbcL*	*trnL-F*	*trnS-G*	*ycf1*
256	*Goniothalamus tenuifolius* King	KM818559	KM818620	KM818669	KM818694	KM818842	KM818889	KM818909	KM818974
257	*Goniothalamus thomsonii* Thwaites	-	KM818621	-	KM818733	KM818834	KM818875	KM818971	-
258	*Goniothalamus thwaitesii* Hook. f. & Thomson	KM818560	KM818622	KM818653	KM818703	-	KM818849	KM818922	-
259	*Goniothalamus tomentosus* R. M. K. Saunders	KM818561	KM818623	-	KM818738	KM818783	KM818846	KM818964	-
260	*Goniothalamus tortilipetalus* M. R. Hend.	-	KM818624	KM818642	KM818708	KM818828	KM818905	KM818948	-
261	*Goniothalamus touranensis* Ast	-	KM818625	-	KM818731	KM818804	KM818870	KM818965	-
262	*Goniothalamus undulatus* Ridl.	KM818562	KM818626	KM818652	KM818679	KM818820	KM818896	KM818921	KM818978
263	*Goniothalamus uvarioides* King	-	KM818627	KM818658	KM818685	KM818827	KM818852	KM818966	KM818975
264	*Goniothalamus velutinus* Airy Shaw	KM818563	KM818628	KM818644	KM818705	KM818812	KM818900	KM818953	KM818989
265	*Goniothalamus woodii* Merr. ex Mat Salleh	KM818564	KM818629	KM818668	KM818720	KM818824	KM818862	KM818972	-
266	*Goniothalamus wrayi* King	KM818565	KM818630	KM818671	KM818721	KM818803	KM818859	KM818957	-
267	*Goniothalamus wynaadensis* (Bedd.) Bedd.	KM818566	KM818631	-	KM818697	KM818816	KM818863	KM818970	KM818991

No.	Voucher information	GenBank accession numbers							
	Taxa name	*atpB-rbcL*	*matK*	*ndhF*	*psbA-trnH*	*rbcL*	*trnL-F*	*trnS-G*	*ycf1*
268	*Greenwayodendron oliveri* (Engl.) Verdc.	AY841377	AY743489	AY841408	AY841465	AY743451	AY743470	AY841555	-
269	*Greenwayodendron suaveolens* (Engl. & Diels) Verdc.	-	-	-	AY841466	AY841524	AY319090	-	-
270	*Guatteria aeruginosa* Standl.	EF179264	AY740909	EF179299	DQ125136	AY740958	AY741007	EF179341	-
271	*Guatteria alata* Maas & van Setten	-	AY740910	-	DQ125137	AY740959	AY741008	-	-
272	*Guatteria allenii* R. E. Fr.	-	AY740911	-	DQ125138	DQ861791	DQ861843	-	-
273	*Guatteria alta* R. E. Fr.	-	DQ125065	-	DQ125139	DQ124941	DQ124999	-	-
274	*Guatteria alutacea* Diels = *Guatteria hirsuta* Ruiz & Pav.	-	AY740912	-	DQ125140	AY740961	AY741010	-	-
275	*Guatteria amplifolia* Triana & Planch.	-	DQ125066	-	DQ125141	DQ124942	DQ125000	-	-
276	*Guatteria anomala* R. E. Fr.	EF179263	AY740913	EF179298	AY841437	AY740962	AY741011	EF179340	-
277	*Guatteria anthracina* Scharf & Maas = *Guatteria liesneri* D. M. Johnson & N. A. Murray	-	DQ861698	-	DQ861748	DQ861792	DQ861844	-	-
278	*Guatteria atra* Sandwith = *Guatteria punctata* (Aubl.) R. A. Howard	-	AY740914	-	DQ125142	AY740963	AY741012	-	-
279	*Guatteria australis* A. St.-Hil.	-	AY740915	-	-	AY740964	AY741013	-	-
280	*Guatteria blainii* (Griseb.) Urb.	-	AY740916	-	DQ861758	DQ861793	DQ861845	-	-
281	*Guatteria blepharophylla* Mart.	-	DQ861738	-	DQ861782	DQ861834	DQ861888	-	-
282	*Guatteria boliviana* H. J. P. Winkl. = *Guatteria ucayalina* Huber	-	DQ125067	-	DQ125144	DQ124943	DQ125001	-	-
283	*Guatteria brevicuspis* R. E. Fr. = *Guatteria blepharophylla* Mart.	-	AY740917	-	DQ125145	AY740966	AY741015	-	-

No.	Voucher information	GenBank accession numbers							
	Taxa name	*atpB-rbcL*	*matK*	*ndhF*	*psbA-trnH*	*rbcL*	*trnL-F*	*trnS-G*	*ycf1*
284	*Guatteria brevipedicellata* R. E. Fr. = *Guatteria hirsuta* Ruiz & Pav.	-	DQ125068	-	DQ125146	DQ124944	DQ125002	-	-
285	*Guatteria burchellii* R. E. Fr. = *Guatteria ferruginea* A. St. -Hil.	-	DQ861701	-	-	DQ861795	DQ861847	-	-
286	*Guatteria caribaea* Urb.	-	AY740918	-	DQ125149	AY740967	AY741016	-	-
287	*Guatteria chiriquiensis* R. E. Fr.	-	AY740919	-	DQ125150	AY740968	AY741017	-	-
288	*Guatteria citriodora* Ducke	-	DQ861702	-	-	DQ861796	DQ861848	-	-
289	*Guatteria decurrens* R. E. Fr.	-	DQ861703	-	-	DQ861798	DQ861850	-	-
290	*Guatteria diospyroides* Baill. = *Guatteria amplifolia* Triana & Planch.	-	AY740920	-	EF179243	AY740969	AY741018	-	-
291	*Guatteria discolor* R. E. Fr.	-	AY740921	-	DQ125153	AY740970	AY741019	-	-
292	*Guatteria dolichopoda* Donn. Sm.	-	DQ861704	-	DQ861752	DQ861800	DQ861852	-	-
293	*Guatteria dumetorum* R. E. Fr. = *Guatteria lucens* Standl.	-	AY740922	-	DQ125154	DQ861799	DQ861851	-	-
294	*Guatteria dusenii* R. E. Fr. = *Guatteria australis* A. St. -Hil.	-	DQ125072	-	DQ125155	DQ124948	DQ125006	-	-
295	*Guatteria ecuadorensis* R. E. Fr. = *Guatteria hirsuta* Ruiz & Pav.	-	DQ861706	-	DQ861753	DQ861802	DQ861854	-	-
296	*Guatteria elata* R. E. Fr.	-	AY740923	-	DQ125156	AY740972	AY741021	-	-
297	*Guatteria elegantissima* R. E. Fr.	-	AY740924	-	DQ125157	AY740973	AY741022	-	-
298	*Guatteria excellens R. E. Fr.* = *Guatteria guianensis* (Aubl.) R. E. Fr.	-	DQ861707	-	DQ861754	DQ861803	DQ861855	-	-
299	*Guatteria ferruginea* A. St.-Hil.	-	DQ125073	-	DQ125158	DQ124949	DQ125007	-	-

No.	Voucher information	GenBank accession numbers							
	Taxa name	*atpB-rbcL*	*matK*	*ndhF*	*psbA-trnH*	*rbcL*	*trnL-F*	*trnS-G*	*ycf1*
300	*Guatteria foliosa* Benth.	-	AY740925	-	DQ125159	AY740974	AY741023	-	-
301	*Guatteria friesiana* (W. A. Rodrigues) Erkens & Maas	-	DQ861740	-	DQ861784	DQ861836	DQ861889	-	-
302	*Guatteria galeottiana* Baill.	-	DQ125074	-	DQ125160	DQ124950	DQ125008	-	-
303	*Guatteria glabrescens* R. E. Fr. = *Guatteria australis* A. St. -Hil.	EF179265	AY740926	EF179300	DQ125161	AY740975	AY741024	EF179342	-
304	*Guatteria gracilipes* R. E. Fr. = *Guatteria punctata* (Aubl.) R. A. Howard	-	DQ125075	-	DQ125162	DQ124951	DQ125009	-	-
305	*Guatteria guianensis* (Aubl.) R. E. Fr.	-	AY740927	-	DQ125163	AY740976	AY741025	-	-
306	*Guatteria heteropetala* Benth. [as *Heteropetalum spruceanum* in GenBank]	-	DQ861745	-	DQ861789	DQ861841	DQ861894	-	-
307	*Guatteria heterotricha* R. E. Fr. = *Guatteria argentea* Erkens & Maas	-	AY740928	-	DQ125164	AY740977	AY741026	-	-
308	*Guatteria hilariana* var. *verruculosa* R. E. Fr. = *Guatteria australis* A.St.-Hil.	-	DQ861710	-	DQ861756	DQ861806	DQ861858	-	-
309	*Guatteria hispida* (R. E. Fr.) Erkens & Maas	-	DQ861741	-	DQ861785	DQ861837	DQ861890	-	-
310	*Guatteria hyposericea* Diels = *Guatteria scytophylla* Diels	-	AY740929	-	DQ125166	AY740978	AY741027	-	-
311	*Guatteria inuncta* R. E. Fr. = *Guatteria amplifolia* Triana & Planch.	-	AY740930	-	DQ125167	AY740979	AY741028	-	-
312	*Guatteria inundata* Mart.	-	AY740931	-	DQ125168	AY740980	AY741029	-	-
313	*Guatteria jefensis* Barringer	-	AY740932	-	DQ125169	DQ861805	DQ861857	-	-
314	*Guatteria juruensis* Diels = *Guatteria hirsuta* Ruiz & Pav.	-	DQ861708	-	-	DQ861804	DQ861856	-	-

No.	Voucher information	GenBank accession numbers							
	Taxa name	*atpB-rbcL*	*matK*	*ndhF*	*psbA-trnH*	*rbcL*	*trnL-F*	*trnS-G*	*ycf1*
315	*Guatteria kuhlmannii* R. E. Fr. = *Guatteria cryandra* Erkens & Maas	-	DQ861742	-	DQ861786	DQ861838	DQ861891	-	-
316	*Guatteria latifolia* (Mart.) R. E. Fr.	-	AY740933	-	DQ125170	AY740982	AY741031	-	-
317	*Guatteria latisepala* R. E. Fr.	-	DQ125077	-	DQ125171	DQ124953	DQ125011	-	-
318	*Guatteria lehmannii* R. E. Fr. = *Guatteria goudotiana* Triana & Planch.	-	DQ861711	-	DQ861757	DQ861807	DQ861859	-	-
319	*Guatteria liesneri* D. M. Johnson & N. A. Murray	-	AY740934	-	DQ125172	AY740983	DQ861860	-	-
320	*Guatteria macropus* Mart.	-	AY740935	-	DQ125174	AY740984	AY741033	-	-
321	*Guatteria maypurensis* Kunth	-	AY740936	-	DQ125175	AY740985	DQ861882	-	-
322	*Guatteria megalophylla* Diels	-	AY740937	-	DQ125176	AY740986	AY741035	-	-
323	*Guatteria mexiae* R. E. Fr. = *Guatteria sellowiana* Schltdl.	-	DQ125080	-	DQ125178	DQ124956	DQ125014	-	-
324	*Guatteria modesta* Diels	-	DQ125081	-	DQ125179	DQ124957	DQ125015	-	-
325	*Guatteria multivenia* Diels = *Guatteria decurrens* R. E. Fr.	-	AY740938	-	DQ125180	AY740987	AY741036	-	-
326	*Guatteria neglecta* (Griseb.) P. Wilson ex Léon & Alain = *Guatteria australis* A.St.-Hil.	-	DQ861713	-	DQ861759	DQ861810	DQ861862	-	-
327	*Guatteria notabilis* Mello-Silva & Pirani	-	DQ125082	-	DQ125181	DQ124958	DQ125016	-	-
328	*Guatteria oligocarpa* Mart.	-	AY740939	-	DQ125182	AY740988	AY741037	-	-
329	*Guatteria olivacea* R. E. Fr. = *Guatteria punctata* (Aubl.) R. A. Howard	-	AY740940	-	DQ125183	AY740989	AY741038	-	-
330	*Guatteria oliviformis* Donn. Sm. = *Guatteria verrucosa* R. E. Fr.	-	AY740941	-	DQ125184	AY740990	AY741039	-	-

No.	Voucher information	GenBank accession numbers							
	Taxa name	*atpB-rbcL*	*matK*	*ndhF*	*psbA-trnH*	*rbcL*	*trnL-F*	*trnS-G*	*ycf1*
331	*Guatteria ouregou* (Aubl.) Dunal	-	AY740942	-	DQ125185	AY740991	AY741040	-	-
332	*Guatteria ovalifolia* R. E. Fr. = *Guatteria punctata* (Aubl.) R. A. Howard	-	DQ861715	-	DQ861761	DQ861812	DQ861864	-	-
333	*Guatteria pacifica* R. E. Fr.	-	DQ125083	-	DQ125186	DQ124960	DQ125018	-	-
334	*Guatteria paraensis* R. E. Fr. = *Guatteria citriodora* Ducke	-	DQ125085	-	DQ125188	DQ124961	DQ125019	-	-
335	*Guatteria parvifolia* R. E. Fr. = *Guatteria australis* A.St.-Hil.	-	AY740943	-	DQ861762	AY740992	AY741041	-	-
336	*Guatteria pittieri* R. E. Fr.	-	AY740944	-	DQ125190	AY740993	AY741042	-	-
337	*Guatteria poeppigiana* Mart. = *Guatteria punctata* (Aubl.) R. A. Howard	-	DQ125086	-	DQ125191	DQ124962	DQ125020	-	-
338	*Guatteria pogonopus* Mart.	-	DQ125087	-	DQ125192	DQ124963	DQ125021	-	-
339	*Guatteria pohliana* Schltdl.	-	DQ125088	-	DQ125193	DQ124964	DQ125022	-	-
340	*Guatteria polyantha* R. E. Fr.	-	DQ125089	-	DQ125194	DQ124965	DQ125023	-	-
341	*Guatteria polycarpa* R. E. Fr. = *Guatteria australis* A.St.-Hil.	-	DQ125090	-	DQ125195	DQ124966	DQ125024	-	-
342	*Guatteria pubens* (Mart.) R. E. Fr.	-	DQ125091	-	DQ125196	DQ124967	DQ125025	-	-
343	*Guatteria pudica* N. Zamora & Maas	JQ513884	AY740945	JQ769093	DQ125197	AY740994	AY741043	-	-
344	*Guatteria punctata* (Aubl.) R. A. Howard	-	AY740946	-	DQ125198	AY740995	AY741044	-	-
345	*Guatteria puncticulata* R. E. Fr. = *Guatteria modesta* Diels	-	AY740947	-	DQ125199	AY740996	AY741045	-	-
346	*Guatteria ramiflora* (D. R. Simpson) Erkens & Maas	-	DQ125064	-	DQ125135	DQ124940	DQ124998	-	-

No.	Voucher information	GenBank accession numbers							
	Taxa name	*atpB-rbcL*	*matK*	*ndhF*	*psbA-trnH*	*rbcL*	*trnL-F*	*trnS-G*	*ycf1*
347	*Guatteria recurvisepala* R. E. Fr. = *Guatteria ucayalina* Huber	-	AY740948	-	DQ125200	AY740997	AY741046	-	-
348	*Guatteria reflexa* R. E. Fr. = *Guatteria australis* A.St.-Hil.	-	DQ125092	-	DQ125201	DQ124968	DQ125026	-	-
349	*Guatteria rigida* R. E. Fr.	-	DQ861718	-	-	DQ861814	DQ861867	-	-
350	*Guatteria rigidipes* R. E. Fr. = *Guatteria dolichopoda* Donn. Sm.	-	DQ125094	-	DQ125203	DQ124970	DQ125028	-	-
351	*Guatteria riparia* R. E. Fr. = *Guatteria inundata* Mart.	-	DQ861719	-	DQ861764	DQ861815	DQ861868	-	-
352	*Guatteria rotundata* Maas & van Setten	-	AY740949	-	DQ125204	AY740998	AY741047	-	-
353	*Guatteria rupestris* Mello-Silva & Pirani	-	AY740950	-	DQ125205	AY740999	AY741048	-	-
354	*Guatteria sabuletorum* R. E. Fr.	-	DQ861721	-	DQ861766	DQ861817	DQ861870	-	-
355	*Guatteria saffordiana* Pittier	-	DQ861720	-	DQ861765	DQ861816	DQ861869	-	-
356	*Guatteria salicifolia* R. E. Fr.	-	AY740951	-	-	AY741000	AY741049	-	-
357	*Guatteria scandens* Ducke	-	DQ125095	-	DQ125207	DQ124971	DQ125029	-	-
358	*Guatteria schlechtendaliana* Mart. = *Guatteria pogonopus* Mart.	-	DQ125096	-	DQ125208	DQ124972	DQ125030	-	-
359	*Guatteria schomburgkiana* Mart. = *Guatteria citriodora* Ducke	-	AY740952	-	DQ125209	AY741001	DQ861871	-	-
360	*Guatteria schunkevigoi* D. R. Simpson = *Guatteria ucayalina* Huber	-	DQ125097	-	DQ125210	DQ124973	DQ125031	-	-
361	*Guatteria scytophylla* Diels	-	AY740953	-	DQ125211	AY741002	AY741051	-	-
362	*Guatteria sellowiana* Schltdl.	-	AY740954	-	DQ125212	AY741003	AY841702	-	-

No.	Voucher information	GenBank accession numbers							
	Taxa name	*atpB-rbcL*	*matK*	*ndhF*	*psbA-trnH*	*rbcL*	*trnL-F*	*trnS-G*	*ycf1*
363	*Guatteria sessilicarpa* Maas & van Setten	-	AY740955	-	DQ125214	DQ124974	DQ125032	-	-
364	*Guatteria sessilis* R. E. Fr. = *Guatteria schomburgkiana* Mart.	-	DQ125099	-	DQ125215	DQ124975	DQ125033	-	-
365	*Guatteria sordida* var. *ovalis* R. E. Fr. = *Guatteria australis* A.St.-Hil.	-	DQ125100	-	DQ125216	DQ124976	DQ125034	-	-
366	*Guatteria sphaerantha* R. E. Fr. = *Guatteria pittieri* R. E. Fr.	-	DQ125101	-	DQ125217	DQ124977	DQ125035	-	-
367	*Guatteria stipitata* R. E. Fr. = *Guatteria ucayalina* Huber.	-	DQ125102	-	DQ125218	DQ124978	DQ125036	-	-
368	*Guatteria subsessilis* Mart.	-	DQ125103	-	DQ125219	DQ124979	DQ125037	-	-
369	*Guatteria talamancana* N. Zamora & Maas	-	DQ861729	-	DQ861773	DQ861825	DQ861878	-	-
370	*Guatteria tomentosa* Rusby 4494 = *Guatteria trichocarpa* Erkens & Maas	-	DQ861737	-	DQ861781	DQ861833	DQ861886	-	-
371	*Guatteria tomentosa* Rusby 9521	-	DQ861730	-	DQ861774	DQ861826	DQ861879	-	-
372	*Guatteria tonduzii* Diels = *Guatteria dolichopoda* Donn. Sm.	-	AY740956	-	DQ125228	AY741005	AY741054	-	-
373	*Guatteria trichoclonia* Diels = *Guatteria tomentosa* Rusby	-	DQ125112	-	DQ125229	DQ861827	DQ125046	-	-
374	*Guatteria ucayaliana* Diels	-	DQ861732	-	DQ861776	DQ861828	DQ861881	-	-
375	*Guatteria venezuelana* R. E. Fr.	-	DQ125113	-	DQ125230	DQ861831	DQ125047	-	-
376	*Guatteria verruculosa* R. E. Fr.	-	DQ125114	-	DQ125231	DQ124990	DQ125048	-	-
377	*Guatteria villosissima* A. St.-Hil.	-	AY740957	-	DQ125232	AY741006	AY741055	-	-
378	*Guatteria wachenheimii* Benoist	-	DQ125115	-	DQ125233	DQ124991	DQ125049	-	-

No.	Voucher information	GenBank accession numbers							
	Taxa name	*atpB-rbcL*	*matK*	*ndhF*	*psbA-trnH*	*rbcL*	*trnL-F*	*trnS-G*	*ycf1*
379	*Guatteria zamorae* Erkens & Maas	-	DQ861736	-	DQ861780	DQ861832	DQ861885	-	-
380	*Hexalobus crispiflorus* A. Rich.	-	-	EU169713	EU169737	EU169760	EU169782	EU169804	-
381	*Hexalobus salicifolius* Engl.	-	-	EU169714	EU169738	EU169761	EU169783	EU169805	-
382	*Hornschuchia citriodora* D. M. Johnson	-	-	-	-	AY841625	AY841703	-	-
383	*Huberantha cerasoides* (Roxb.) Chaowasku	-	AY518854	JQ889985	JQ889980	-	AY319131	-	JQ723950
384	*Huberantha decora* (Diels) Chaowasku	-	-	JX544879	JX544859	-	JX544869	-	JX544849
385	*Huberantha henrici* (Diels) Chaowasku	-	-	JX544880	JX544860	-	JX544870	-	JX544850
386	*Huberantha jenkinsii* (Hook. f. & Thomson) Chaowasku	-	-	JX544842	JX544812	-	JX544803	-	JX544821
387	*Huberantha korinti* (Dunal) Chaowasku	EU522345	EU522234	JX544877	EU522124	EU522289	EU522179	-	JX544847
388	*Huberantha nitidissima* (Dunal) Chaowasku	-	JQ889989	JQ889986	JQ889981	-	JQ889988	-	JQ889976
389	*Huberantha pendula* (Capuron ex G. E. Schatz & Le Thomas) Chaowasku	-	AY518852	JQ889987	JQ889982	-	AY319144	-	JQ889977
390	*Huberantha perrieri* (Cavaco & Keraudren) Chaowasku	-	-	JX544881	JX544861	-	JX544871	-	JX544851
391	*Huberantha rumphii* (Blume ex Hensch.) Chaowasku	-	AY518791	JX544841	JX544811	-	AY319145	-	JX544820
392	*Huberantha stuhlmannii* (Engl.) Chaowasku	-	AY518853	JX544882	JX544862	-	AY319149	-	JX544852
393	*Huberantha tanganyikensis* (Vollesen) Chaowasku	-	-	JX544883	JX544863	-	JX544872	-	JX544853
394	*Isolona campanulata* Engl. & Diels	EF179266	AY238963	EF179301	DQ125127	AY238954	EF179318	EF179343	-
395	*Isolona capuronii* Cavaco & Keraudren	-	-	-	EU216662	-	EU216708	EU216617	-
396	*Isolona cauliflora* Verdc.	-	-	EU169716	EU169739	EU169762	EU169784	EU169807	-

No.	Voucher information	GenBank accession numbers							
	Taxa name	*atpB-rbcL*	*matK*	*ndhF*	*psbA-trnH*	*rbcL*	*trnL-F*	*trnS-G*	*ycf1*
397	*Isolona congolana* (De Wild. & T. Durand) Engl. & Diels 3852	-	-	EU216637	EU216658	-	EU216704	EU216613	-
398	*Isolona congolana* (De Wild. & T. Durand) Engl. & Diels 9550	-	-	EU216644	EU216668	-	EU216714	EU216623	-
399	*Isolona cooperi* Hutch. & Dalziel ex G. P. Cooper & Record	-	-	EU216636	EU216657	AY841626	AY841704	EU216612	-
400	*Isolona dewevrei* (De Wild. & T. Durand) Engl. & Diels	-	-	EU216645	EU216669	-	EU216715	EU216624	-
401	*Isolona ghesquierei* Cavaco & Keraudren	-	-	-	EU216663	-	EU216709	EU216618	-
402	*Isolona heinsenii* Engl. & Diels	-	-	EU216640	EU216664	-	EU216710	EU216619	-
403	*Isolona hexaloba* Pierre ex Engl. & Diels	-	-	EU169717	EU169740	EU169763	EU169785	EU169808	-
404	*Isolona linearis* Couvreur	-	-	EU216641	EU216665	-	EU216711	EU216620	-
405	*Isolona perrieri* Diels	-	-	EU216639	EU216661	-	EU216707	EU216616	-
406	*Isolona pleurocarpa* Diels	-	-	EU216642	EU216666	-	EU216712	EU216621	-
407	*Isolona thonneri* (De Wild. & T. Durand) Engl. & Diels	-	-	EU216643	EU216667	-	EU216713	EU216622	-
408	*Isolona zenkeri* Engl.	-	-	EU216638	EU216660	-	EU216705	EU216614	-
409	*Klarobelia candida* Chatrou	-	-	-	AY841467	AY841525	AY841539	-	-
410	*Klarobelia cauliflora* Chatrou	-	-	-	AY841468	AY841627	AY841705	-	-
411	*Klarobelia inundata* Chatrou	AY841378	AY743490	AY841409	AY841469	AY743452	AY743471	AY841556	-
412	*Klarobelia megalocarpa* Chatrou	-	AY518866	-	AY841470	-	AY319176	-	-
413	*Klarobelia stipitata* Chatrou	-	-	-	AY841472	AY841628	AY841706	-	-
414	*Klarobelia* sp.	-	-	-	AY841471	AY841526	AY841540	-	-

No.	Voucher information	GenBank accession numbers							
	Taxa name	*atpB-rbcL*	*matK*	*ndhF*	*psbA-trnH*	*rbcL*	*trnL-F*	*trnS-G*	*ycf1*
415	*Letestudoxa bella* Pellegr.	EF179267	DQ125059	EF179302	DQ125128	AY841629	AY841707	EF179344	-
416	*Letestudoxa glabrifolia* Chatrou & Repetur	-	-	-	-	AY841630	AY841708	-	-
417	*Lettowianthus stellatus* Diels	-	-	-	EU169730	EU169753	EU169775	EU169797	-
418	*Liriodendron chinense* (Hemsl.) Sarg.	-	-	-	AY841424	AY841593	AY841670	-	-
419	*Maasia discolor* (Diels) Mols, Keßler & Rogstad	AY841385	AY518872	AY841416	AY841500	-	AY319135	AY841563	-
420	*Maasia glauca* (Hassk.) Mols, Keßler & Rogstad	-	AY518871	-	AY841501	GQ248676	AY319137	-	-
421	*Maasia multinervis* (Diels) Mols, Keßler & Rogstad	-	JF810383	-	-	JF810395	JF810407	-	-
422	*Maasia ovalifolia* (Rogstad) Mols, Keßler & Rogstad	-	JF810384	-	-	JF810396	JF810408	-	-
423	*Maasia sumatrana* (Miq.) Mols, Keßler & Rogstad	AY841387	AY518873	AY841418	AY841503	-	AY319153	AY841565	-
424	*Magnolia kobus* DC.	-	AY743476	-	AY841425	AY743438	AY743457	-	-
425	*Malmea dielsiana* R. E. Fr.	AY841379	AY238964	AY841410	AY841473	AY238955	AY319177	AY841557	-
426	*Malmea dimera* Chatrou	-	-	-	AY841474	AY841631	AY841709	-	-
427	*Malmea* sp. Chatrou et al 8	AY841380	AY841397	AY841411	AY841475	AY841527	AY841541	AY841558	-
428	*Malmea surinamensis* Chatrou	-	AY743491	-	AY841476	AY743453	AY743472	-	-
429	*Marsypopetalum crassum* (R. Parker) B. Xue & R. M. K. Saunders	-	HQ286571	JQ723792	-	HQ286577	HQ286583	-	JQ723929
430	*Marsypopetalum littorale* (Blume) B. Xue & R. M. K. Saunders	-	AY518835	JX544827	JX544804	-	AY319140	-	JX544813

No.	Voucher information	GenBank accession numbers							
	Taxa name	*atpB-rbcL*	*matK*	*ndhF*	*psbA-trnH*	*rbcL*	*trnL-F*	*trnS-G*	*ycf1*
431	*Marsypopetalum lucidum* (Merr.) B. Xue & R. M. K. Saunders	-	HQ286572	-	-	HQ286578	HQ286584	-	-
432	*Marsypopetalum triste* (Pierre) B. Xue & R. M. K. Saunders	-	HQ286573	-	-	HQ286579	HQ286585	-	-
433	*Meiocarpidium lepidotum* (Oliv.) Engl. & Diels	-	-	-	EU169731	EU169754	EU169776	EU169798	-
434	*Meiogyne baillonii* (Guillaumin) Heusden	-	JQ723768	JQ723793	-	JQ723855	JQ723908	-	JQ723930
435	*Meiogyne bidwillii* (Benth.) D. C. Thomas, Chaowasku & R. M. K. Saunders	-	AY518795	JQ723789	-	JQ723851	AY319089	-	JQ723925
436	*Meiogyne glabra* Heusden	-	JQ723772	-	-	JQ723859	JQ723912	-	JQ723935
437	*Meiogyne hainanensis* (Merr.) Bân	-	JQ723773	-	-	JQ723860	-	-	JQ723936
438	*Meiogyne heteropetala* (F. Muell.) D. C. Thomas, Chaowasku & R. M. K. Saunders	-	JQ723766	JQ723790	-	JQ723853	JQ723906	-	JQ723927
439	*Meiogyne hirsuta* (Jessup) Jessup	-	JQ723774	JQ723798	-	JQ723861	JQ723914	-	JQ723937
440	*Meiogyne lecardii* (Guillaumin) Heusden	-	JQ723775	JQ723799	-	JQ723862	JQ723915	-	JQ723938
441	*Meiogyne mindorensis* (Merr.) Heusden	-	JQ723776	JQ723800	-	JQ723863	JQ723916	-	JQ723939
442	*Meiogyne monosperma* (Hook. f. & Thomson) Heusden	-	JQ723777	-	-	JQ723864	-	-	JQ723940
443	*Meiogyne pannosa* (Dalzell) J. Sinclair	-	JQ723778	JQ723801	-	JQ723865	JQ723918	-	JQ723941
444	*Meiogyne stenopetala* (F. Muell.) Heusden	-	JQ723779	JQ723803	-	JQ723866	AY319083	-	JQ723943
445	*Meiogyne verrucosa* Jessup	-	JQ723780	JQ723804	-	JQ723867	JQ723920	-	JQ723944
446	*Meiogyne virgata* (Blume) Miq.	-	AY518798	JX544769	JX544784	-	AY319094	-	JQ723945
447	*Mezzettia parviflora* Becc.	-	AY518881	-	-	-	AY319095	-	-
448	*Miliusa amplexicaulis* Ridl.	-	-	JQ690479	JQ690480	-	JQ690478	-	JQ690481

No.	Voucher information	GenBank accession numbers							
	Taxa name	*atpB-rbcL*	*matK*	*ndhF*	*psbA-trnH*	*rbcL*	*trnL-F*	*trnS-G*	*ycf1*
449	*Miliusa brahei* (F. Muell.) Jessup	-	-	JQ690431	JQ690432	-	JQ690430	-	JQ690433
450	*Miliusa campanulata* Pierre	-	AY518842	JQ690487	JQ690488	-	AY319096	-	JQ690489
451	*Miliusa cuneata* Craib	-	AY518844	JQ690491	JQ690492	-	AY319097	-	JQ690493
452	*Miliusa fusca* Pierre	-	-	JQ690443	JQ690444	-	JQ690442	-	JQ690445
453	*Miliusa horsfieldii* (Benn.) Pierre	-	AY518849	JQ690447	JQ690448	-	AY319098	-	JQ690449
454	*Miliusa indica* Lesch. ex A. DC.	-	JQ723781	JQ723806	-	JQ723868	JQ723921	-	JQ723946
455	*Miliusa koolsii* (Kosterm.) J. Sinclair	-	-	JQ690455	JQ690456	-	JQ690454	-	JQ690457
456	*Miliusa lanceolata* Chaowasku & Keßler	-	-	JQ690459	JQ690460	-	JQ690458	-	JQ690461
457	*Miliusa macrocarpa* Hook. f. & Thomson	-	JQ690499	JQ690500	JQ690501	-	JQ690498	-	JQ690502
458	*Miliusa macropoda* Miq.	-	-	JQ690463	JQ690464	-	JQ690462	-	JQ690465
459	*Miliusa montana* Gardner ex Hook. f. & Thomson	-	JQ690507	JQ690508	JQ690509	-	JQ690506	-	JQ690510
460	*Miliusa novoguineensis* Mols & Keßler	-	-	JQ690467	JQ690468	-	JQ690466	-	JQ690469
461	*Miliusa parviflora* Ridl.	-	-	JQ690471	JQ690472	-	JQ690470	-	JQ690473
462	*Miliusa sclerocarpa* (A. DC.) Kurz	-	-	JQ690475	JQ690476	-	JQ690474	-	JQ690477
463	*Miliusa thorelii* Finet & Gagnep.	-	AY518846	JQ690519	JQ690520	-	AY319104	-	JQ690521
464	*Miliusa traceyi* Jessup	-	JQ690532	JQ690533	JQ690534	-	JQ690531	-	JQ690535
465	*Miliusa velutina* (Dunal) Hook. f. & Thomson	-	AY518847	JQ690536	JQ690537	-	AY319105	-	JQ690538
466	*Mischogyne michelioides* Exell	-	-	EU169718	EU169741	EU169764	EU169786	EU169809	-
467	*Mitrella kentii* (Blume) Miq.	-	FJ743751	JQ768616	FJ743789	AY841633	AY841711	-	-
468	*Mitrephora alba* Ridl.	-	AY518855	JQ889983	JQ889978	-	AY319106	-	JQ723947
469	*Mitrephora celebica* Scheff.	-	AY518859	-	-	-	AY319107	-	-
470	*Mitrephora keithii* Ridl.	EU522343	AY518857	-	EU522122	-	AY319108	-	-

No.	Voucher information	GenBank accession numbers							
	Taxa name	*atpB-rbcL*	*matK*	*ndhF*	*psbA-trnH*	*rbcL*	*trnL-F*	*trnS-G*	*ycf1*
471	*Mitrephora maingayi* Hook. f. & Thomson	-	AY518856	-	-	-	AY319109	-	-
472	*Mitrephora polypyrena* (Blume) Miq.	-	AY518858	-	-	-	AY319110	-	-
473	*Mitrephora vittata* Weeras. & R. M. K. Saunders	-	FJ463223	-	-	-	FJ463224	-	-
474	*Mkilua fragrans* Verdc.	EF179268	-	EF179303	DQ861696	AY841634	AY841712	EF179345	-
475	*Monanthotaxis buchananii* (Engl.) Verdc.	-	JQ768581	JQ768617	JQ768660	JQ768700	JQ768742	-	-
476	*Monanthotaxis congoensis* Baill.	-	JQ768582	JQ768618	JQ768661	JQ768701	JQ768743	-	-
477	*Monanthotaxis diclina* (Sprague) Verdc.	-	JQ768584	JQ768620	JQ768663	JQ768703	JQ768745	-	-
478	*Monanthotaxis enghiana* (Diels) P. H. Hoekstra	-	JQ768578	JQ768613	JQ768657	JQ768697	JQ768739	-	-
479	*Monanthotaxis fornicata* (Baill.) Verdc.	-	JQ768583	JQ768619	JQ768662	JQ768702	JQ768744	-	-
480	*Monanthotaxis obovata* (Benth.) P. H. Hoekstra	-	JQ768579	JQ768614	JQ768658	JQ768698	JQ768740	-	-
481	*Monanthotaxis schweinfurthii* (Engl. & Diels) Verdc.	-	JQ768585	JQ768621	JQ768664	JQ768704	JQ768746	-	-
482	*Monanthotaxis trichocarpa* (Engl. & Diels) Verdc.	-	JQ768586	JQ768622	JQ768665	JQ768705	JQ768747	-	-
483	*Monanthotaxis whytei* (Stapf) Verdc.	EF179269	EF179278	EF179304	EF179315	AY841635	AY841713	EF179346	-
484	*Monocarpia euneura* Miq.	AY841381	AY518865	AY841412	AY841477	-	AY319111	AY841559	-
485	*Monocarpia marginalis* (Scheff.) J. Sinclair	-	JQ690397	JQ690398	JQ690399	JQ690395	JQ690396	-	JQ690400
486	*Monocyclanthus vignei* Keay	-	-	EU169719	EU169742	EU169765	EU169787	EU169810	-
487	*Monodora angolensis* Welw.	-	-	EU216648	EU216672	-	EU216718	EU216627	-
488	*Monodora carolinae* Couvreur	-	-	EU216653	EU216677	-	EU216723	EU216632	-
489	*Monodora crispata* Engl.	-	-	EU169720	EU169743	AY841637	AY841715	EU169811	-
490	*Monodora globiflora* Couvreur	-	-	EU216654	EU216678	-	EU216724	EU216633	-

No.	Voucher information	GenBank accession numbers							
	Taxa name	*atpB-rbcL*	*matK*	*ndhF*	*psbA-trnH*	*rbcL*	*trnL-F*	*trnS-G*	*ycf1*
491	*Monodora grandidieri* Baill.	-	-	EU216649	EU216673	-	EU216719	EU216628	-
492	*Monodora hastipetala* Couvreur	-	-	EU216655	EU216679	-	EU216725	EU216634	-
493	*Monodora junodii* Engl. & Diels	-	-	EU216651	EU216675	-	EU216721	EU216630	-
494	*Monodora laurentii* De Wild.	-	-	EU216650	EU216674	-	EU216720	EU216629	-
495	*Monodora minor* Engl. & Diels	-	-	EU216656	EU216680	-	EU216726	EU216635	-
496	*Monodora myristica* (Gaertn.) Dunal	EF179270	AY743485	EF179305	DQ125129	AY743447	EU216716	EF179347	-
497	*Monodora stenopetala* Oliv.	-	-	EU216652	EU216676	-	EU216722	EU216631	-
498	*Monodora tenuifolia* Benth.	-	-	EU216647	EU216671	AY841638	AY841716	EU216626	-
499	*Monodora undulate* (P. Beauv.) Couvreur	-	-	EU169722	EU169744	EU169766	EU169788	EU169813	-
500	*Monoon australe* (Benth.) B. Xue & R. M. K. Saunders	-	JX227872	-	-	JX227897	JX227849	-	-
501	*Monoon erianthoides* (Airy Shaw) B. Xue & R. M. K. Saunders	-	JX227875	-	-	JX227900	JX227851	-	-
502	*Monoon fuscum* (King) B. Xue & R. M. K. Saunders	-	AY518787	JX544779	JX544792	-	AY319085	-	JX544767
503	*Monoon hypogaeum* (King) B. Xue & R. M. K. Saunders	-	JX227876	-	-	JX227901	JX227852	-	-
504	*Monoon kingii* (Baker f.) B. Xue & R. M. K. Saunders	-	JX227877	-	-	JX227902	JX227853	-	-
505	*Monoon klemmei* (Elmer) B. Xue & R. M. K. Saunders	-	JX227878	-	-	JX227903	JX227854	-	-
506	*Monoon laui* (Merr.) B. Xue & R. M. K. Saunders	-	JX227879	-	-	JX227904	JX227855	-	-

No.	Voucher information	GenBank accession numbers							
	Taxa name	*atpB-rbcL*	*matK*	*ndhF*	*psbA-trnH*	*rbcL*	*trnL-F*	*trnS-G*	*ycf1*
507	*Monoon membranifolium* (J. Sinclair) B. Xue & R. M. K. Saunders	-	AY518788	-	-	-	AY319086	-	-
508	*Monoon michaelii* (C.T. White) B. Xue & R. M. K. Saunders	-	JX227880	-	-	JX227905	JX227856	-	-
509	*Monoon paradoxum* (Becc.) B. Xue & R. M. K. Saunders	-	AY518789	-	-	-	AY319087	-	-
510	*Monoon sympetalum* (Merr.) B. Xue & R. M. K. Saunders	-	JX227881	-	-	JX227906	JX227857	-	-
511	*Moonon coffeoides* (Thwaites ex Hook. f. & Thomson) B. Xue & R. M. K. Saunders	EU522344	EU522233	-	EU522123	EU522288	EU522178	-	-
512	*Moonon lateriflorum* (Blume) B. Xue & R. M. K. Saunders	-	AY518781	JQ723811	-	JQ723870	AY319138	-	JQ723951
513	*Moonon longifolium* (Sonn.) B. Xue & R. M. K. Saunders	EU522346	AY518786	-	EU522125	EU522290	EU522180	-	-
514	*Moonon obtusum* (Craib) B. Xue & R. M. K. Saunders	-	AY518784	JX544780	JX544793	-	AY319154	-	JX544768
515	*Mosannona costaricensis* (R. E. Fr.) Chatrou	AY841382	AY743503	AY841413	AY841479	AY743510	AY743496	AY841560	-
516	*Mosannona discolor* (R. E. Fr.) Chatrou	-	AY743504	-	AY841480	AY743511	AY743497	-	-
517	*Mosannona garwoodii* Chatrou & Welzenis	-	AY743505	-	AY841481	AY743512	AY743498	-	-
518	*Mosannona pacifica* Chatrou	-	AY743506	-	AY841482	AY743513	AY743499	-	-
519	*Mosannona papillosa* Chatrou	-	AY743507	-	AY841483	AY743514	AY743500	-	-
520	*Mosannona vasquezii* Chatrou	-	AY743508	-	AY841484	AY743515	AY319178	-	-
521	*Mwasumbia alba* Couvreur & D. M. Johnson	-	-	-	-	EU747680	EU747674	-	-
522	*Myristica fragrans* Houtt.	-	AJ966803	AY218188	-	AF206798	-	-	-

No.	Voucher information	GenBank accession numbers							
	Taxa name	*atpB-rbcL*	*matK*	*ndhF*	*psbA-trnH*	*rbcL*	*trnL-F*	*trnS-G*	*ycf1*
523	*Neostenanthera myristicifolia* (Oliv.) Exell	EF179271	AY743486	EF179306	DQ125130	AY743448	AY743467	EF179348	-
524	*Neo-uvaria acuminatissima* (Miq.) Airy Shaw	-	AY518793	-	-	-	AY319112	-	-
525	*Neo-uvaria parallelivenia* (Boerl.) H. Okada & K. Ueda	-	AY518794	-	-	-	AY319113	-	-
526	*Neo-uvaria telopea* Chaowasku	-	JX544751	JX544778	JX544791	JX544755	JX544783	-	JX544766
527	*Onychopetalum periquino* (Rusby) D. M. Johnson & N. A. Murray	AY841383	AY518876	AY841414	AY841485	-	AY319179	AY841561	-
528	*Ophrypetalum odoratum* Diels	-	-	EU169723	EU169745	EU169767	EU169789	EU169814	-
529	*Orophea brandisii* Hook. f. & Thomson	-	AY518813	-	-	-	AY319116	-	-
530	*Orophea celebica* (Blume) Miq.	-	AY518814	-	-	-	AY319117	-	-
531	*Orophea creaghii* (Ridl.) Leonardía & Keßler	-	AY518817	-	-	-	AY319118	-	-
532	*Orophea enneandra* Blume	-	AY518816	-	-	-	AY319120	-	-
533	*Orophea enterocarpa* Maingay ex Hook. f. & Thomson	-	AY518815	JQ690416	JQ690417	-	AY319119	-	JQ690418
534	*Orophea kerrii* Keßler	-	AY518818	JQ690419	JQ690420	-	AY319121	-	JQ690421
535	*Orophea polycarpa* A. DC.	-	AY518819	-	-	-	AY319123	-	-
536	*Oxandra asbeckii* (Pulle) R. E. Fr.	-	-	-	AY841486	AY841639	AY841717	-	-
537	*Oxandra espintana* (Spruce ex Benth.) Baill.	-	DQ018260	-	AY841487	-	AY319180	-	-
538	*Oxandra euneura* Diels	-	-	-	AY841488	AY841640	AY841718	-	-
539	*Oxandra lanceolata* (Sw.) Baill.	-	KJ012702	-	KJ426857	KJ082471	-	-	-
540	*Oxandra laurifolia* (Sw.) A. Rich.	-	HM446726	-	AY841489	AY841528	AY841542	-	-
541	*Oxandra longipetala* R. E. Fr.	-	-	-	AY841490	AY841641	AY841719	-	-
542	*Oxandra macrophylla* R. E. Fr.	-	-	-	AY841491	AY841642	AY841720	-	-

No.	Voucher information	GenBank accession numbers							
	Taxa name	*atpB-rbcL*	*matK*	*ndhF*	*psbA-trnH*	*rbcL*	*trnL-F*	*trnS-G*	*ycf1*
543	*Oxandra nitida* R. E. Fr.	-	-	-	AY841492	AY841529	AY841543	-	-
544	*Oxandra polyantha* R. E. Fr.	-	-	-	AY841493	AY841643	AY841721	-	-
545	*Oxandra riedeliana* R. E. Fr.	-	KP859350	-	-	-	KP859336	-	-
546	*Oxandra sphaerocarpa* R. E. Fr.	-	-	-	AY841494	AY841644	AY841722	-	-
547	*Oxandra venezuelana* R. E. Fr.	-	JQ690413	JQ690414	AY841495	AY841645	AY841723	-	JQ690415
548	*Oxandra xylopioides* Diels	-	-	-	AY841496	AY841646	AY841724	-	-
549	*Phaeanthus ebracteolatus* (C. Presl) Merr.	-	AY518863	-	-	JF738713	AY319125	-	-
550	*Phaeanthus* sp. WP2A0446	-	GQ248183	-	GQ248373	GQ248677	-	-	-
551	*Phaeanthus splendens* Miq.	-	AY518864	JX544777	JX544790	JX544754	AY319126	-	JX544765
552	*Phoenicanthus obliquus* (Hook. f. & Thomson) Alston	MF322677*	MF322639*	-	MF322658*	MF322654*	MF322667*	-	-
553	*Piptostigma fasciculatum* (De Wild.) Boutique ex R. E. Fr.	-	-	-	AY841497	AY841647	AY841725	-	-
554	*Piptostigma mortehani* De Wild.	AY841384	AY743492	AY841415	AY841498	AY743454	AY743473	AY841562	-
555	*Piptostigma oyemense* Pellegr.	-	KC627444	-	KC667604	KC627994	-	-	-
556	*Piptostigma pilosum* Oliv.	-	-	-	AY841499	AY841648	AY319181	-	-
557	*Platymitra macrocarpa* Boerl.	-	AY518812	JQ723809	JQ690423	-	AY319127	-	JQ690424
558	*Platymitra* sp. 1 TC-2012	-	JQ690426	JQ690427	JQ690428	-	JQ690425	-	JQ690429
559	*Polyalthia angustissima* Ridl.	-	JX227882	JX544831	JX544807	JX227907	JX544795	-	-
560	*Polyalthia bullata* King	-	JX544825	JX544839	JX544809	JX227908	JX544800	-	JX544818
561	*Polyalthia cauliflora* Hook. f. & Thomson	-	AY518823	JX544837	-	-	AY319129	-	-
562	*Polyalthia celebica* Miq.	-	AY518827	JX544838	JX544808	-	AY319130	-	-
563	*Polyalthia evecta* (Pierre) Finet & Gagnep.	-	JX227885	-	-	JX227910	JX227861	-	-

No.	Voucher information	GenBank accession numbers							
	Taxa name	*atpB-rbcL*	*matK*	*ndhF*	*psbA-trnH*	*rbcL*	*trnL-F*	*trnS-G*	*ycf1*
564	*Polyalthia hispida* B. Xue & R. M. K. Saunders	-	JX227886	-	-	JX227911	JX227862	-	-
565	*Polyalthia insignis* (Hook. f.) Airy Shaw	-	JX227887	-	-	JX227912	JX227863	-	-
566	*Polyalthia johnsonii* (F. Muell.) B. Xue & R. M. K. Saunders	-	JQ723767	JQ723791	JX544810	JQ723854	JX544801	-	JQ723928
567	*Polyalthia kanchanaburiana* Khumch. & Thongp.	-	JX227888	-	-	JX227913	JX227864	-	-
568	*Polyalthia lanceolata* S. Vidal	-	JX227889	-	-	JX227914	JX227865	-	-
569	*Polyalthia lateritia* J. Sinclair	-	JX227890	-	-	JX227915	JX227866	-	-
570	*Polyalthia longirostris* (Scheff.) B. Xue & R. M. K. Saunders	-	AY518826	-	-	-	AY319091	-	-
571	*Polyalthia motleyana* (Hook. f.) Airy Shaw	-	JX227891	-	-	JX227916	JX227867	-	-
572	*Polyalthia parviflora* Ridl.	-	JX227892	JX544836	-	JX227917	JX544799	-	-
573	*Polyalthia stenopetala* (Hook. f. & Thomson) Finet & Gagnep.	-	JX227896	JX544832	-	JX544823	AY319148	-	-
574	*Polyalthia suberosa* (Roxb.) Thwaites	AY841386	AY220439	AY841417	AY841502	AY238956	AY319152	AY841564	JQ723952
575	*Polyalthia submontana* (Jessup) B. Xue & R. M. K. Saunders	-	JX227893	-	-	JX227918	JX227869	-	-
576	*Polyalthia trochilia* I. M. Turner	-	JX227894	-	-	JX227919	JX227870	-	-
577	*Polyalthia xanthocarpa* B. Xue & R. M. K. Saunders	-	JX227895	-	-	JX227920	JX227871	-	-
578	*Polyceratocarpus askhambryan-iringae* A. R. Marshall & D. M. Johnson	-	-	-	-	EU747681	EU747675	-	-
579	*Polyceratocarpus microtrichus* (Engl. & Diels) Ghesq. ex Pellegr.	-	-	-	-	EU747683	EU747677	-	-

No.	Voucher information	GenBank accession numbers							
	Taxa name	*atpB-rbcL*	*matK*	*ndhF*	*psbA-trnH*	*rbcL*	*trnL-F*	*trnS-G*	*ycf1*
580	*Polyceratocarpus parviflorus* (Baker f.) Ghesq.	-	KC627837	-	KC688815	EU747682	EU747676	-	-
581	*Polyceratocarpus pellegrinii* Le Thomas	-	-	-	-	EU747684	EU747678	-	-
582	*Popowia hirta* Miq.	-	AY518860	JX544830	JX544806	-	AY319156	-	JX544816
583	*Popowia odoardi* Diels	-	AY518861	-	-	-	AY319157	-	-
584	*Popowia pisocarpa* (Blume) Endl.	-	AY518862	JQ723812	-	-	AY319158	-	JQ723953
585	*Porcelia steinbachii* (Diels) R. E. Fr.	-	-	-	-	AY841649	AY841727	-	-
586	*Pseudartabotrys letestui* Pellegr.	EF179272	DQ125061	EF179307	DQ125131	AY841650	AY841728	EF179349	-
587	*Pseudephedranthus fragrans* (R. E. Fr.) Aristeg.	-	-	-	AY841504	AY841651	AY841729	-	-
588	*Pseudephedranthus fragrans* (R. E. Fr.) Aristeg. *Pfa683*	MF322675*	MF322637*	-	MF322656*	MF322652*	MF322665*	-	-
589	*Pseudephedranthus fragrans* (R. E. Fr.) Aristeg. *Pfa878*	MF322676*	MF322638*	-	MF322657*	MF322653*	MF322666*	-	-
590	*Pseudomalmea diclina* (R. E. Fr.) Chatrou	AY841388	AY841398	AY841419	AY841506	AY841530	AY319182	AY841566	-
591	*Pseudomalmea* sp. *Idarraga 13*	-	-	-	AY841507	AY841652	AY841730	-	-
592	*Pseudoxandra longipes* Maas	-	-	-	AY841509	AY841532	AY841546	-	-
593	*Pseudoxandra lucida* R. E. Fr.	AY841389	AY518870	AY841420	AY841510	-	AY319190	AY841567	-
594	*Pseudoxandra polyphleba* (Diels) R. E. Fr.	-	JQ769091	JQ769092	AY841512	AY841654	AY841732	-	-
595	*Pseudoxandra spiritus-sancti* Maas	AY841390	AY841399	AY841421	AY841513	AY841533	AY841547	AY841568	-
596	*Pseuduvaria acerosa* Y. C. F. Su & R. M. K. Saunders	EU522347	EU522236	-	EU522126	EU522291	EU522181	-	-
597	*Pseuduvaria aurantiaca* (Miq.) Merr.	EU522348	EU522237	-	EU522127	EU522292	EU522182	-	-
598	*Pseuduvaria beccarii* (Scheff.) J. Sinclair	EU522349	EU522238	-	EU522128	EU522293	EU522183	-	-

No.	Voucher information	GenBank accession numbers							
	Taxa name	*atpB-rbcL*	*matK*	*ndhF*	*psbA-trnH*	*rbcL*	*trnL-F*	*trnS-G*	*ycf1*
599	*Pseuduvaria borneensis* Y. C. F. Su & R. M. K. Saunders	EU522350	EU522239	-	EU522129	EU522294	EU522184	-	-
600	*Pseuduvaria brachyantha* Y. C. F. Su & R. M. K. Saunders	EU522351	AY518837	-	EU522130	EU522295	AY319160	-	-
601	*Pseuduvaria bruneiensis Y.C.F. Su & R.M.K. Saunders*	EU522352	EU522241	-	EU522131	EU522296	EU522186	-	-
602	*Pseuduvaria calliura* Airy Shaw	EU522353	EU522242	-	EU522132	EU522297	EU522187	-	-
603	*Pseuduvaria cerina* J. Sinclair	EU522354	EU522243	-	EU522133	EU522298	EU522188	-	-
604	*Pseuduvaria clemensiae* Y. C. F. Su & R. M. K. Saunders	EU522355	EU522244	-	EU522134	EU522299	EU522189	-	-
605	*Pseuduvaria coriacea* Y. C. F. Su & R. M. K. Saunders	EU522356	AY518838	-	EU522135	EU522300	AY319161	-	-
606	*Pseuduvaria costata* (Scheff.) J. Sinclair	EU522357	EU522246	-	EU522136	EU522301	EU522191	-	-
607	*Pseuduvaria cymosa* (J. Sinclair) Y. C. F. Su & R. M. K. Saunders	EU522358	EU522247	-	EU522137	EU522302	EU522192	-	-
608	*Pseuduvaria dielsiana* (Lauterb.) J. Sinclair	EU522359	EU522248	-	EU522138	EU522303	EU522193	-	-
609	*Pseuduvaria dolichonema* (Diels) J. Sinclair	EU522360	EU522249	-	EU522139	EU522304	EU522194	-	-
610	*Pseuduvaria filipes* (Lauterb. & K. Schum.) J. Sinclair	EU522361	EU522250	-	EU522140	EU522305	EU522195	-	-
611	*Pseuduvaria fragrans* Y. C. F. Su, Chaowasku & R. M. K. Saunders	-	JQ723784	JX544829	-	JQ723871	JQ723924	-	JQ723954
612	*Pseuduvaria froggattii* (F. Muell.) Jessup	EU522362	EU522251	-	EU522141	EU522306	EU522196	-	-
613	*Pseuduvaria galeata* J. Sinclair	EU522363	EU522252	-	EU522142	EU522307	EU522197	-	-

No.	Voucher information	GenBank accession numbers							
	Taxa name	*atpB-rbcL*	*matK*	*ndhF*	*psbA-trnH*	*rbcL*	*trnL-F*	*trnS-G*	*ycf1*
614	*Pseuduvaria glabrescens* (Jessup) Y. C. F. Su & R. M. K. Saunders	EU522364	EU522253	-	EU522143	EU522308	EU522198	-	-
615	*Pseuduvaria grandifolia* (Warb.) J. Sinclair	EU522365	EU522254	-	EU522144	EU522309	EU522199	-	-
616	*Pseuduvaria hylandii* Jessup	EU522366	EU522255	-	EU522145	EU522310	EU522200	-	-
617	*Pseuduvaria kingiana* Y. C. F. Su & R. M. K. Saunders	EU522367	EU522256	-	EU522146	EU522311	EU522201	-	-
618	*Pseuduvaria latifolia* (Blume) Bakh. f.	EU522368	EU522257	-	EU522147	EU522312	EU522202	-	-
619	*Pseuduvaria lignocarpa* J. Sinclair	EU522369	EU522258	-	EU522148	EU522313	EU522203	-	-
620	*Pseuduvaria luzonensis* (Merr.) Y. C. F. Su & R. M. K. Saunders	EU522370	EU522259	-	EU522149	EU522314	EU522204	-	-
621	*Pseuduvaria macgregorii* Merr.	EU522371	EU522260	-	EU522150	EU522315	EU522205	-	-
622	*Pseuduvaria macrocarpa* (Burck) Y. C. F. Su & R. M. K. Saunders	EU522372	EU522261	-	EU522151	EU522316	EU522206	-	-
623	*Pseuduvaria macrophylla* (Oliv.) Merr.	EU522373	EU522262	-	EU522152	EU522317	EU522207	-	-
624	*Pseuduvaria megalopus* (K. Schum.) Y. C. F. Su & Mols 15599	-	AY518836	-	-	-	AY319124	-	-
625	*Pseuduvaria megalopus (*K. Schum.) Y. C. F. Su & Mols 16235	EU522374	EU522263	-	EU522153	EU522318	EU522208	-	-
626	*Pseuduvaria mindorensis* Y. C. F. Su & R. M. K. Saunders	EU522375	EU522264	-	EU522154	EU522319	EU522209	-	-
627	*Pseuduvaria mollis* (Warb.) J. Sinclair	EU522376	EU522265	-	EU522155	EU522320	EU522210	-	-
628	*Pseuduvaria monticola* J. Sinclair	EU522377	EU522266	-	EU522156	EU522321	EU522211	-	-
629	*Pseuduvaria mulgraveana* Jessup	EU522378	EU522267	-	EU522157	EU522322	EU522212	-	-

No.	Voucher information	GenBank accession numbers							
	Taxa name	*atpB-rbcL*	*matK*	*ndhF*	*psbA-trnH*	*rbcL*	*trnL-F*	*trnS-G*	*ycf1*
630	*Pseuduvaria multiovulata* (C. E. C. Fisch.) J. Sinclair	EU522379	EU522268	-	EU522158	EU522323	EU522213	-	-
631	*Pseuduvaria nova-guineensis* J. Sinclair	EU522380	EU522269	-	EU522159	EU522324	EU522214	-	-
632	*Pseuduvaria obliqua* Y. C. F. Su & R. M. K. Saunders	EU522381	EU522270	-	EU522160	EU522325	EU522215	-	-
633	*Pseuduvaria oxycarpa* (Boerl. ex Koord.) Y. C. F. Su & R. M. K. Saunders	EU522382	EU522271	-	EU522161	EU522326	EU522216	-	-
634	*Pseuduvaria pamattonis* (Miq.) Y. C. F. Su & R. M. K. Saunders	EU522383	AY518840	-	EU522162	EU522327	AY319163	-	-
635	*Pseuduvaria parvipetala* Y. C. F. Su & R. M. K. Saunders	EU522384	EU522273	-	EU522163	EU522328	EU522218	-	-
636	*Pseuduvaria philippinensis* Merr.	EU522385	EU522274	-	EU522164	EU522329	EU522219	-	-
637	*Pseuduvaria phuyensis* (R. M. K. Saunders, Y. C. F. Su & Chalermglin) Y. C. F. Su & R. M. K. Saunders	EU522342	AY518841	-	EU522121	EU522287	AY319114	-	-
638	*Pseuduvaria reticulata* (Blume) Miq.	EU522386	EU522275	-	EU522165	EU522330	EU522220	-	-
639	*Pseuduvaria rugosa* (Blume) Merr.	EU522387	AY518839	-	EU522166	EU522331	AY319162	-	-
640	*Pseuduvaria sessilicarpa* (J. Sinclair) Y. C. F. Su & R. M. K. Saunders	EU522388	EU522277	-	EU522167	EU522332	EU522222	-	-
641	*Pseuduvaria sessilifolia* J. Sinclair	EU522389	EU522278	-	EU522168	EU522333	EU522223	-	-
642	*Pseuduvaria setosa* (King) J. Sinclair	EU522390	EU522279	-	EU522169	EU522334	EU522224	-	-
643	*Pseuduvaria silvestris* (Diels) J. Sinclair	EU522391	EU522280	-	EU522170	EU522335	EU522225	-	-
644	*Pseuduvaria subcordata* Y. C. F. Su & R. M. K. Saunders	EU522392	EU522281	-	EU522171	EU522336	EU522226	-	-

No.	Voucher information	GenBank accession numbers							
	Taxa name	*atpB-rbcL*	*matK*	*ndhF*	*psbA-trnH*	*rbcL*	*trnL-F*	*trnS-G*	*ycf1*
645	*Pseuduvaria taipingensis* J. Sinclair	EU522393	EU522282	-	EU522172	EU522337	EU522227	-	-
646	*Pseuduvaria trimera* (Craib) Y. C. F. Su & R. M. K. Saunders	EU522394	EU522283	-	EU522173	EU522338	EU522228	-	-
647	*Pseuduvaria unguiculata* (Elmer) Y. C. F. Su & R. M. K. Saunders	EU522395	EU522284	-	EU522174	EU522339	EU522229	-	-
648	*Pseuduvaria villosa* Jessup	EU522396	EU522285	-	EU522175	EU522340	EU522230	-	-
649	*Pyramidanthe prismatica* (Hook. f. & Thomson) J. Sinclair	-	JN175163	-	JN175178	JN175193	JN175208	-	-
650	*Ruizodendron ovale* (Ruiz & Pav.) R. E. Fr.	-	-	-	AY841514	AY841657	AY841735	-	-
651	*Sageraea lanceolata* Miq.	-	AY518799	JX544774	JX544787	-	AY319164	-	JX544762
652	*Sanrafaelia ruffonammari* Verdc.	-	-	EU169724	EU169746	EU169768	EU169790	EU169815	-
653	*Sapranthus microcarpus* (Donn. Sm.) R. E. Fr.	-	AY518806	-	-	-	AY319166	-	-
654	*Sapranthus palanga* R. E. Fr.	-	JQ586518	-	-	JQ590191	-	-	-
655	*Sapranthus viridiflorus* G. E. Schatz	AY841391	AY743493	AY841422	AY841515	JQ590194	AY319165	AY841569	JQ723955
656	*Schefferomitra subaequalis* (Scheff.) Diels	-	KX786606	-	KX786626	KX786628	KX786645	-	-
657	*Sirdavidia solannona* Couvreur & Sauquet	-	-	-	-	KP144082	KP144080	-	-
658	*Sphaerocoryne gracilis* (Oliv. ex Engl. & Diels) Verdc.	-	-	JQ768623	EU169732	EU169755	EU169777	EU169799	-
659	*Sphaerocoryne* sp. 07/4	-	AY518878	JQ768624	FJ743788	AY319071	AY319185	-	-
660	*Stelechocarpus burahol* (Blume) Hook. f. & Thomson	-	AY518803	JX544775	JX544788	-	AY319167	-	JQ723956
661	*Stenanona costaricensis* R. E. Fr.	-	AY518801	JX544772	AY841516	JQ590198	AY319183	-	JX544759
662	*Stenanona panamensis* Standl.	-	AY518802	-	-	-	AY319184	-	-

No.	Voucher information	GenBank accession numbers							
	Taxa name	*atpB-rbcL*	*matK*	*ndhF*	*psbA-trnH*	*rbcL*	*trnL-F*	*trnS-G*	*ycf1*
663	*Tetrameranthus duckei* R. E. Fr.	-	-	-	AY841439	AY841658	AY841736	-	-
664	*Tetrameranthus laomae* D. R. Simpson	-	-	-	-	AY841659	AY841737	-	-
665	*Toussaintia orientalis* Verdc.	-	-	EU169710	EU169733	EU169756	EU169778	EU169800	-
666	*Tridimeris hahniana* (Baill.) Baill.	-	-	-	-	-	AY319169	-	-
667	*Tridimeris* sp. TC-2012	-	JX544750	JX544773	JX544786	JX544753	JX544782	-	JX544761
668	*Trigynaea duckei* (R. E. Fr.) R. E. Fr.	-	-	-	-	AY841660	AY841738	-	-
669	*Trigynaea lanceipetala* D. M. Johnson & N. A. Murray	EF179274	AY743487	EF179309	-	AY743449	AY743468	EF179351	-
670	*Trivalvaria costata* (Hook. f. & Thomson) I. M. Turner	-	-	JQ723815	-	-	-	-	JQ723957
671	*Trivalvaria dubia* (Kurz) J. Sinclair	-	HQ286574	-	-	HQ286580	HQ286587	-	-
672	*Trivalvaria macrophylla* (Blume) Miq.	-	HQ286576	-	-	HQ286582	HQ286588	-	-
673	*Trivalvaria* sp. 1 TC-2012	-	JX544824	JX544828	JX544805	JX544822	JX544794	-	JX544814
674	*Unonopsis pittieri* Saff.	-	GQ982122	-	AY841517	AY841661	DQ018201	-	-
675	*Unonopsis stipitata* Diels	AY841392	AY841400	AY841423	AY841519	AY841662	DQ018202	AY841570	-
676	*Uvaria acuminata* Oliv.	-	FJ743753	-	FJ743791	FJ743825	FJ743860	-	-
677	*Uvaria afzelii* G. Elliot	-	JN175164	-	JN175179	JN175194	JN175209	-	-
678	*Uvaria angolensis* Welw. ex Oliv.	-	FJ743754	-	FJ743792	FJ743826	FJ743861	-	-
679	*Uvaria anonoides* Baker f.	-	FJ743755	-	FJ743793	FJ743827	FJ743862	-	-
680	*Uvaria antsiranensis* Le Thomas	-	JN175165	-	JN175180	JN175195	JN175210	-	-
681	*Uvaria argenteum* Blume	-	FJ743741	-	FJ743778	FJ743816	FJ743850	-	-
682	*Uvaria baumannii* Engl. & Diels	-	FJ743756	-	FJ743794	FJ743828	FJ743863	-	-
683	*Uvaria boniana* Finet & Gagnep.	-	FJ743757	-	FJ743795	FJ743829	FJ743864	-	-

No.	Voucher information	GenBank accession numbers							
	Taxa name	*atpB-rbcL*	*matK*	*ndhF*	*psbA-trnH*	*rbcL*	*trnL-F*	*trnS-G*	*ycf1*
684	*Uvaria borneensis* (Merr.) Utteridge	-	FJ743758	-	FJ743796	FJ743830	FJ743865	-	-
685	*Uvaria buchholzii* Engl. & Diels	-	GU951715	-	GU951724	GU951733	GU951742	-	-
686	*Uvaria calamistrata* Hance	-	FJ743759	-	FJ743797	FJ743831	FJ743866	-	-
687	*Uvaria chamae* P. Beauv.	-	FJ743760	-	FJ743798	AY841663	AY841741	-	-
688	*Uvaria cherrevensis* (Pierre ex Finet & Gagnep.) L. L. Zhou, Y. C. F. Su & R. M. K. Saunders	-	FJ743750	-	FJ743787	FJ743823	FJ743858	-	-
689	*Uvaria clavata* Pierre ex Engl. & Diels	-	FJ743761	-	FJ743799	FJ743833	FJ743867	-	-
690	*Uvaria commersoniana* Baill.	-	JN175166	-	JN175181	JN175196	JN175211	-	-
691	*Uvaria concava* Teijsm. & Binn.	-	JN175167	-	JN175182	JN175197	JN175212	-	-
692	*Uvaria cordata* (Dunal) Alston	-	JN175168	-	JN175183	JN175198	JN175213	-	-
693	*Uvaria cuneifolia* (Hook.f. & Thomson) L. L. Zhou, Y. C. F. Su & R. M. K. Saunders	-	FJ743749	-	FJ743786	FJ743822	FJ743857	-	-
694	*Uvaria dasoclema* L. L. Zhou, Y. C. F. Su & R. M. K. Saunders	-	GU951716	-	GU951725	GU951734	GU951743	-	-
695	*Uvaria dependens* Engl. & Diels	-	JN175169	-	JN175184	JN175199	JN175214	-	-
696	*Uvaria dulcis* Dunal	-	FJ743740	-	FJ743777	FJ743815	FJ743849	-	-
697	*Uvaria elmeri* Merr.	-	JN175170	-	JN175185	JN175200	JN175215	-	-
698	*Uvaria excelsum* (Hook.f. & Thomson) King	-	FJ743742	-	FJ743779	FJ743817	FJ743851	-	-
699	*Uvaria faulknerae* Verdc.	-	FJ743762	-	FJ743800	FJ743834	FJ743868	-	-
700	*Uvaria flava* Teijsm. & Binn.	-	FJ743763	-	FJ743801	FJ743835	FJ743869	-	-
701	*Uvaria glabra* Span	-	FJ743743	-	FJ743780	FJ743818	FJ743852	-	-
702	*Uvaria grandiflora* Roxb. ex Hornem.	-	FJ743764	-	FJ743802	FJ743836	FJ743870	-	-

No.	Voucher information	GenBank accession numbers							
	Taxa name	*atpB-rbcL*	*matK*	*ndhF*	*psbA-trnH*	*rbcL*	*trnL-F*	*trnS-G*	*ycf1*
703	*Uvaria griffithii L. L. Zhou, Y. C. F. Su & R. M. K. Saunders*	-	FJ743746	-	FJ743783	AY841607	AY841685	-	-
704	*Uvaria hahnii* (Finet & Gagnep.) J. Sinclair	-	JN175171	-	JN175186	JN175201	JN175216	-	-
705	*Uvaria hamiltonii* Hook. f. & Thomson	-	FJ743765	-	FJ743803	FJ743837	FJ743871	-	-
706	*Uvaria hirsuta* Jack	-	FJ743766	-	FJ743804	FJ743838	FJ743872	-	-
707	*Uvaria hookeri* King	-	FJ743744	-	FJ743781	AY841606	AY841684	-	-
708	*Uvaria laha* Miq.	-	JN175172	-	JN175187	JN175202	JN175217	-	-
709	*Uvaria leichhardtii* (F. Muell.) L. L. Zhou, Y. C. F. Su & R. M. K. Saunders	-	GU951717	-	GU951726	GU951735	GU951744	-	-
710	*Uvaria leptocladon* Oliv.	-	FJ743767	-	FJ743805	FJ743839	FJ743873	-	-
711	*Uvaria lobbiana* Hook. f. & Thomson	-	FJ743768	-	FJ743806	FJ743840	FJ743874	-	-
712	*Uvaria lucida* Benth.	EF179275	AY238966	EF179310	AY841440	AY238957	EF179319	EF179352	-
713	*Uvaria lurida* Hook. f. & Thomson	-	FJ743769	-	FJ743807	FJ743841	FJ743875	-	-
714	*Uvaria macropoda* Hook. f. & Thomson	-	FJ743770	-	FJ743808	FJ743842	FJ743876	-	-
715	*Uvaria manjensis* Cavaco & Keraudren	-	JN175173	-	JN175188	JN175203	JN175218	-	-
716	*Uvaria marenteria* (DC.) Baill.	-	JN175174	-	JN175189	JN175204	JN175219	-	-
717	*Uvaria micrantha* (A.DC.) Hook. f. & Thomson	-	FJ743745	-	FJ743782	FJ743819	FJ743854	-	-
718	*Uvaria pandensis* Verdc.	-	FJ743771	-	FJ743809	FJ743843	FJ743877	-	-
719	*Uvaria pauciovulata* Hook. f. & Thomson	-	JN175175	-	JN175190	JN175205	JN175220	-	-
720	*Uvaria rufa* Blume	-	FJ743772	-	FJ743810	FJ743844	FJ743878	-	-
721	*Uvaria rupestris* (Jessup) L. L. Zhou, Y. C. F. Su & R. M. K. Saunders	-	GU951718	-	GU951727	GU951736	GU951745	-	-
722	*Uvaria sambiranensis* Deroin & L. Gaut.	-	JN175176	-	JN175191	JN175206	JN175221	-	-

No.	Voucher information	GenBank accession numbers							
	Taxa name	*atpB-rbcL*	*matK*	*ndhF*	*psbA-trnH*	*rbcL*	*trnL-F*	*trnS-G*	*ycf1*
723	*Uvaria sankowskyi* L. L. Zhou, Y. C. F. Su & R. M. K. Saunders	-	GU951719	-	GU951728	GU951737	GU951746	-	-
724	*Uvaria scabridula* (Jessup) L. L. Zhou, Y. C. F. Su & R. M. K. Saunders	-	GU951720	-	GU951729	GU951738	GU951747	-	-
725	*Uvaria semecarpifolia* Hook. f. & Thomson	-	FJ743773	-	FJ743811	FJ743845	FJ743879	-	-
726	*Uvaria siamensis* (Scheff.) L. L. Zhou, Y. C. F. Su & R. M. K. Saunders	-	FJ743752	-	FJ743790	FJ743824	FJ743859	-	-
727	*Uvaria sphenocarpa* Hook. f. & Thomson	-	FJ743774	-	FJ743812	FJ743846	FJ743880	-	-
728	*Uvaria topazensis* (Jessup) L. L. Zhou, Y. C. F. Su & R. M. K. Saunders	-	GU951721	-	GU951730	GU951739	GU951748	-	-
729	*Uvaria uhrii* (F. Muell.) L. L. Zhou, Y. C. F. Su & R. M. K. Saunders	-	GU951722	-	GU951731	GU951740	GU951749	-	-
730	*Uvaria unguiculata* (Jessup) L. L. Zhou, Y. C. F. Su & R. M. K. Saunders	-	GU951723	-	GU951732	GU951741	GU951750	-	-
731	*Uvaria versicolor* Pierre ex Engl. & Diels	-	FJ743775	-	FJ743813	FJ743847	FJ743881	-	-
732	*Uvaria welwitschii* (Hiern) Engl. & Diels	-	JN175177	-	JN175192	JN175207	JN175222	-	-
733	*Uvaria wrayi* (King) L. L. Zhou, Y. C. F. Su & R. M. K. Saunders	-	FJ743747	-	FJ743784	FJ743821	FJ743856	-	-
734	*Uvaria zeylanica* Aubl. ex Willd.	-	FJ743776	-	FJ743814	FJ743848	FJ743882	-	-
735	*Uvariastrum insculptum* (Engl. & Diels) Sprague & Hutch.	-	-	EU169725	EU169747	EU169769	EU169791	-	-
736	*Uvariastrum pierreanum* Engl.	-	-	-	EU169748	EU169770	EU169792	EU169816	-
737	*Uvariastrum pynaertii* De Wild.	-	KC627652	-	KC667802	KC628249	-	-	-
738	*Uvariodendron connivens* (Benth.) R. E. Fr.	-	-	-	KC688780	-	-	-	-

No.	Voucher information	GenBank accession numbers							
	Taxa name	*atpB-rbcL*	*matK*	*ndhF*	*psbA-trnH*	*rbcL*	*trnL-F*	*trnS-G*	*ycf1*
739	*Uvariodendron kirkii* Verdc.	-	-	EU169726	EU169749	EU169771	EU169793	EU169817	-
740	*Uvariodendron molundense* (Diels) R. E. Fr.	-	-	EU169727	EU169750	EU169772	EU169794	EU169818	-
741	*Uvariodendron* sp. PM4812	-	KC627429	-	KC667591	KC627977	-	-	-
742	*Uvariopsis bakeriana* (Hutch. & Dalziel) Robyns & Ghesq.	-	KC627573	-	KC667743	KC628178	-	-	-
743	*Uvariopsis korupensis* Gereau & Kenfack	-	KC627757	EU169729	EU169752	EU169774	EU169796	EU169820	-
744	*Uvariopsis tripetala* (Baker f.) G. E. Schatz	-	-	EU169712	EU169735	EU169758	EU169780	EU169802	-
745	*Uvariopsis vanderystii* Robyns & Ghesq.	-	-	EU169728	EU169751	EU169773	EU169795	EU169819	-
746	*Wangia saccopetaloides* (W. T. Wang) X. Guo & R. M. K. Saunders	-	KF680920	KF680923	KF680924	KF680926	KF680930	-	KF680932
747	*Winitia cauliflora* (Scheff.) Chaowasku	-	AY518800	JX544776	JX544789	-	AY319168	-	JX544764
748	*Winitia expansa* Chaowasku	-	KC857617	KC857618	KC857619	-	KC857616	-	KC857620
749	*Xylopia acutiflora* (Dunal) A. Rich.	-	KC627830	-	KC668016	KC627989	-	-	-
750	*Xylopia aethiopica* (Dunal) A. Rich.	-	KC627474	-	KC667638	KC628042	-	-	-
751	*Xylopia ferruginea* (Hook. f. & Thomson) Hook. f. & Thomson	-	DQ125063	EF179311	DQ125133	AY841666	AY841744	-	-
752	*Xylopia frutescens* Aubl.	-	JQ586527	-	AY841441	AY841667	AY841745	-	-
753	*Xylopia nitida* Ast	-	FJ514650	-	GQ428667	FJ037960	-	-	-
754	*Xylopia peruviana* R. E. Fr.	EF179276	AY238967	EF179312	DQ125134	AY238958	EF179320	EF179353	-
